# A Positive Feedback Loop of E2F4-Mediated Activation of MNX1 Regulates Tumour Progression in Colorectal Cancer

**DOI:** 10.7150/jca.86718

**Published:** 2023-09-04

**Authors:** Jia-Ke Li, Hai Liu, Hui-Wen Zhang, Jun Li, Zhuo-Tao Liang

**Affiliations:** 1 Department of General Surgery, The Third Xiangya Hospital of Central South University, Changsha, 410013, China.; 2 National Clinical Research Center for Geriatric Disorders, Xiangya Hospital, Central South University, Changsha, China.

**Keywords:** *MNX1*, * E2F4*, Colorectal Cancer (CRC), diagnostic biomarker

## Abstract

**Purpose:** Colorectal cancer (CRC) is the 3rd most prevalent malignant tumour globally. Although significant strides have been made in diagnosis and treatment, its prognosis at the moment remains unpromising. Therefore, there is an urgent and desperate need to identify novel biomarkers of CRC and evaluate its mechanism of tumourigenesis and development.

**Methods:** JASPAR and RNAinter databases are used to analyze target genes associated with colorectal cancer. Western blotting, q-PCR and immunohistochemistry et, al. were used to detect the level of MNX1 in patients with colorectal cancer, and Chip-PCR was used to detect the targeted binding ability of E2F4 and MNX1. The cells and animal models overexpressed MNX1 and E2F4 were constructed by shRNA transfection.

**Results:** Herein, MNX1 was highly expressed and linked to favourable overall survival curves in colorectal cancer. The functional assay revealed that MNX1 overexpression could promote proliferation, migration, and invasion of CRC cells. Based on the prediction of the JASPAR and RNAinter databases, the transcription factor, E2F4, was bound to the MNX1 promoter region. The Chromatin Immunoprecipitation (ChIP) assay verified the interactions between MNX1 and E2F4 in CRC. Additionally, we found that sh-E2F4 markedly downregulated the MNX1 levels and reduced CRC progression *in vivo* and *in vitro*, which reversed MNX1 overexpression.

**Conclusion:** Therefore, our research discovered that E2F4-mediated abnormal MNX1 expression promotes CRC progression and could become a novel diagnostic or therapeutic target of CRC.

## Introduction

Colorectal cancer (CRC) is the 3^rd^ most common malignant tumour, with more than 881000 annual fatalities across the globe 1. It is the 5th leading cancer in China, with the highest number of cancer-related deaths in both sexes 2. Whilst researchers have made the exciting progress in diagnosis and treatment, such as more precise personalized treatments 3 microbial-based treatments and diagnostic tools 4, CRC prognosis is currently not promising. Therefore, it is necessary to identify novel CRC biomarkers and investigate their mechanism of tumorigenesis and development to provide effective treatments.

Motor neuron and pancreas homeobox 1 (*MNX1*), also known as *HLXB9*, *SCRA1,* and *HB9,* is a member of the *EHG* homeobox gene family and located on chromosome 7q36.3 5. MNX1 is a transcription factor that regulates the development and differentiation of cells and tissues 6. Additional studies revealed that *MNX1* expression is strongly upregulated in different cancers, including breast 7, colon 8, prostate 5, and liver cancers 9. Furthermore, recent research on CRC confirmed that MNX1-AS1 (MNX1 antisense RNA1), A LncRNA with partial exon overlap with the MNX1 gene, promotes colon adenocarcinoma progression 10. But the mechanism by which MNX1 is improved remains unclear.

*MNX1* expression in CRC was first detected using The Cancer Genome Atlas (TCGA) database and quantitative reverse transcription-polymerase chain reaction (qRT‐PCR). Based on the RNAinter database, we speculated that *E2F4* could bind to the *MNX1* promoter region as a potential upstream regulator. Current studies demonstrate that the *E2F* family comprising *E2F1* and *E2F4* regulates the cell cycle tumour development and progression 11. Recent research discovered that *E2F4* is a potential novel biomarker for breast cancer prognosis 12. In colon cancer, E2F4 was found upregulated and its level was correlated with the clinical stage of colon cancer 13, 14. Nonetheless, the regulatory role of the *E2F4/MNX1* pathway in tumorigenesis and progression still needs to be explored.

This study explores the expression level and interaction of *MNX1* and *E2F4* in CRC using bioinformatics analyses and functional assays. Meanwhile, *MNX1* and *E2F4* effects on cell migration, invasion and tumour growth were examined through *in vivo* and *in vitro* assays. Our findings revealed that the *E2F4/MNX1* pathway could be a promising biomarker and therapeutic target for CRC.

## Methods

### Bioinformatics

GSE41258 and GDS4382 datasets were downloaded from the National Centre for Biotechnology Information (https://www.ncbi.nlm.nih.gov/). Visual analysis was performed using the NetworkAnalyst software (https://www.networkanalyst.ca). GEPIA (http://gepia.cancer-pku.cn/index.html) and StarBase (http://starbase.sysu.edu.cn) were used to analyze *MNX1* and *E2F4* expressions in colorectal cancer and adjacent normal tissues as well as the disease-free survival (DFS) of *MNX1* and *E2F4* in colorectal cancer patients. The target relationship between *E2F4* and *MNX1* was predicted using the RNAinter website (http://www.rna-society.org/). Moreover, the ChIPBase website (http://rna.sysu.edu.cn/chipbase/) discovered a positive correlation between the two genes. The potential binding sites for *MNX1* were predicated using the JASPAR website (http://jaspardev.genereg.net/).

### Clinical Samples and Cell Lines

All studies were approved by the Ethics Committee of the Third Xiangya Hospital of Central South University (approval number: No. KUAI I 21007), and informed consent was obtained from all subjects. Clinical colorectal cancer and other normal tissue samples were selected from 20 patients (12 tumour patients and 8 normal patients) at the Third Xiangya Hospital of Central South University (Table S1-S4). Normal colorectal epithelial cells (NCM460) and colorectal cancer cell lines (SW480, HCT116 and SW620) were purchased from the Cell Bank of the Chinese Academy of Science (Shanghai, China). All cells were grown in RPMI-1640 medium supplemented with 10% FBS.

Inclusion criteria were as follows: (1) Patients diagnosed with colorectal cancer by histopathological examination after tumour resection; (2) Matched healthy controls were selected from asymptomatic patients with negative colonoscopy results; (3) Patients aged between 18 and 80 years, regardless of sex. The following conditions were excluded: (1) Psychiatric disorders, pregnant or lactating women; (2) Suffering from other primary digestive diseases or tumour lesions related to hereditary diseases; (3) Preoperative radiotherapy or chemotherapy; (4) Currently suffering from inflammation, autoimmune diseases, etc.

### Plasmid constructs and transfections

The full-length cDNA of human *MNX1* was PCR-amplified and cloned into the pcDNA 3.1 vectors to construct the overexpression cell lines. Meanwhile, human *MNX1*- and *E2F4*-targeting shRNA sequences were cloned into the pcDNA 3.1 vectors to construct knockdown cell lines. The shRNA sequences we selected were: *MNX1*:sh#1:GCAGGAAGCGGAGAAACAGAA; *E2F4*:sh#1:CGGCGGATTTACGACATT. According to the manufacturer's instructions, HCT116, and SW620 cells were seeded in 6-well plates (Eppendorf, Hamburg, Germany), then Lipofectamine™ 3000 (Invitrogen, Carlsbad CA, USA) was utilized to transfect the plasmid. The cells were collected for subsequent experiments after transfection for 48 h.

### Real-time quantitative PCR

Total RNA was isolated from the above cells or frozen tissues using TRIzol reagent. Subsequently, cDNA was synthesized from 1 μg of total RNA by reverse transcription using the PrimeScript RT reagent Kit. cDNA was amplified using SYBR Green Premix following the manufacturer's instructions. PCR was performed under the following cycling conditions: 10 min at 95°C, followed by 40 cycles at 95°C for 10 s and 60°C for 30 s. Relative quantification analysis was performed using the comparative CT (2 ^-∆∆Ct^) method. The primer sequences used in the experiment are presented in Table 1.

### Immunohistochemistry assay

After paraffin sectioning, four groups of paraffin-embedded specimens were sliced, deparaffinized, rehydrated, and treated with 3% hydrogen peroxide for 15 min to block endogenous peroxidase activity. Thereafter, the sections were overnight incubated with the primary antibodies at 4°C. The sections were then washed in PBS and treated with biotinylated secondary antibodies. Eventually, the sections were counterstained with hematoxylin, dehydrated, mounted, and observed under a light microscope.

### Western blotting

Proteins were isolated from CRC cells and tissues using RIPA lysis buffer (Thermo Scientific, USA) for Western blot, following the manufacturer's instructions. Protein concentrations were determined using the bicinchoninic acid assay (Beyotime Biotechnology, China). Equal amounts of protein were separated using SDS-PAGE on a 10% gel, then transferred onto polyvinylidene difluoride membranes, followed by blocking with 5% skim milk for 2 h. After incubation with primary antibodies (including *MNX1* antibody: 1:1000, Abcam, ab92606, USA; *E2F4* antibody: 1:500, Proteintech, 10923-1-AP, China; β-actin: 1:2000, Proteintech, 66009-1-Ig, China), the samples were washed 4 times (10 min for each wash), then, the membranes were incubated with secondary antibodies (Millipore, Bedford, USA) for 2 h at room temperature. We wrapped the PVDF membrane in the film. Under the condition of red light in the darkroom, the film is processed by developer and fixative. Once dried, the protein bands can be seen directly. Then the film image is converted into a grayscale image by Photoshop software. In the process, the background of the image is converted to gray from blue.

### Proliferation assay

Cell growth was assessed using an MTT assay. The transfected cells were seeded in 96-well plates at 37°C in 5% CO_2_ for 24 h. Each well was incubated with 50 μl of MTT solution at 37°C for 4 h. Thus, the growth medium was removed, and 150 µL of DMSO was added to each well. Finally, the optical density of each well was recorded at 570 nm using a microplate reader.

### Scratch assay

HCT116 and SW620 cells were seeded at a density of 1x10^5^ cells/well in 24-well plates until they were 90% confluent. Scrape wounds were generated using a 20-µl pipette tip; then, cells were cultured with a serum-free medium for 48 h. Wound closure was monitored and photographed at 0, 24, and 48 h using microscope.

### Migration and Invasion Experiments

Cell migration and invasion assays were conducted in 6-well Transwell chambers (8 μm filter pore, Corning) pre-coated or not precoated with Matrigel basement membrane gel. HCT116 and SW620 cells were seeded in the upper chambers, and a culture medium supplemented with 10% was added to the lower chambers. After 24 h of incubation, cells were fixed with 95% ethanol and then stained with hematoxylin. Finally, the cells were observed under a light microscope and quantified by manual counting.

### Chromatin Immunoprecipitation (ChIP)-PCR

HCT116 and SW620 cells were cultured in 150 mm dishes, then 1% formaldehyde was added to the cultures and incubated at room temperature for 10 min. Thereafter, glycine was added to neutralize the remaining formaldehyde. The cells were washed using cold PBS and added to PBS containing protease inhibitor mixture. After centrifugation, the supernatant was obtained as the cell lysate. The proteins were sonicated to shear chromosomal DNA after successfully crosslinking with chromatin. The supernatants were overnight incubated with anti-E2F4 antibody and normal rabbit IgG at 4°C to couple antigen to antibody, then immunoprecipitated by protein A/G to precipitate the immune complexes. After eluting the protein-DNA complexes and reversing the crosslinks, the DNA was purified before analyzing using real-time PCR. Table 2 shows the primer sequences used in the experiment.

### Xenograft assay

The Animal Ethics Committee approved the animal experiments of Central South University. A total of 18 BALB/c-nu mice (4-8 weeks old, half males and half females) were purchased from the SLAC Experimental Animal Limited Company (Shanghai, China). The mice were randomly divided into three groups using a random number generator. HCT116 cells (1 × 106 cells) were subcutaneously injected into the right flank of each mouse. After 20 days, tumour cells were collected, weighed, and paraffin-embedded. Eventually, fluorescence microscopy detected Ki-67-positive cells (1:800, Proteintech, 27309-1-AP, Wuhan, China).

### Statistical analysis

All statistical analyses were conducted using the GraphPad Prism 7 (GraphPad Software, Inc., La Jolla, CA, USA), and the experiments were independently repeated three times. The student's t-test (unpaired or paired) was used to determine the differences between the two groups. In contrast, the ANOVA test was used to determine the differences between the multiple groups. The GEPIA site examined the relationship between the expressions of two genes and the overall survival rates of colorectal cancer patients using the log-rank test. The expression correlations were analyzed through the Spearman correlation analysis from the ChipBase. Fisher's exact test was used to evaluate clinical characteristics in relation to MNX1 and E2F4. A difference with a P value of less than 0.05 (P <0.05) was considered statistically significant.

## Results

### *MNX1* overexpression in CRC tissues and correlation with clinical stage

**Figure 1:**
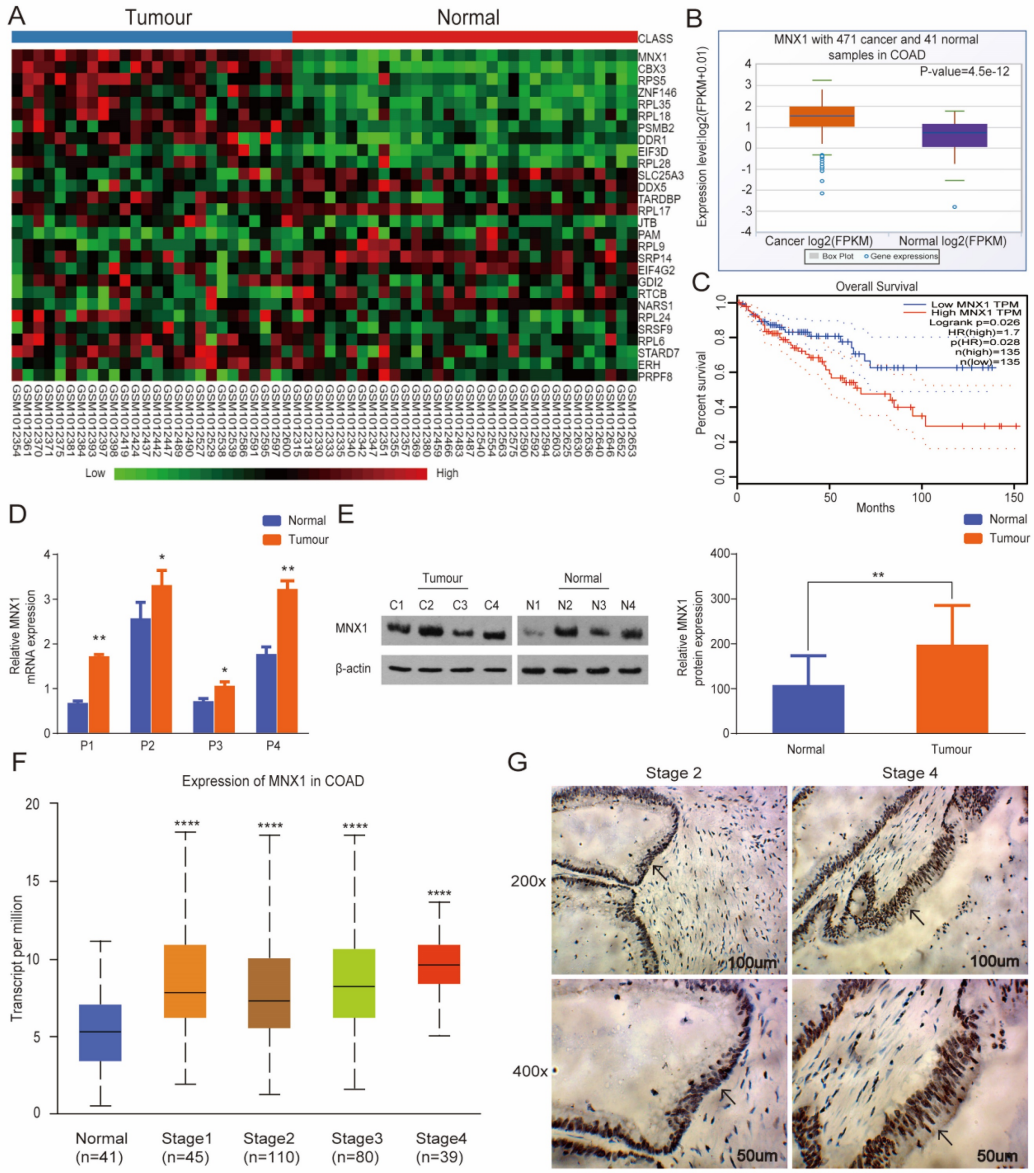
*MNX1* is highly expressed in colorectal cancer tissues. (A) Data from GSE41258: The differentially expressed mRNAs are illustrated in a heatmap. *MNX1* was included in the overexpression category; (B) *MNX1* was upregulated in the tumour tissues compared to the adjacent normal tissues in the StarBase database (P=4.5e-12); (C) Patients with high *MNX1* expression had poor disease-free survival (P=0.026); (D, E) *MNX1* expression determined by qRT-PCR and Western blot assays; (F) *MNX1* expression in different stages; (G) Immunohistochemistry assay was used to detect the expression of *MNX1* in colorectal tumours. Mean ± SD (n = 3 independent experiments). *P < 0.05, **P < 0.01, ****P < 0.0001.

Bioinformatics assays were performed based on GSE41258 and found its overexpression in CRC tissues (Fig. 1A). At the same time, analysis from GDS4382 confirmed similar results (Fig. S1). Additionally, we analyzed the GEPIA and StarBase databases and discovered significant *MNX1* upregulation in colorectal cancer. Also, low *MNX1* expression was linked to a relatively longer overall survival duration (Fig. 1B, C). To further examine this issue, we first collected CRC, and normal tissues were extracted, then *MNX1* expression was detected via qRT‐PCR and Western blot.

Consequently, MNX1 expression was higher in the CRC tissues than in the normal tissues (Fig. 1D, E). In contrast with that in the human colorectal epithelial mucosa cell line NCM460, the mRNA and protein expression of *MNX1* was upregulated in the human CRC cell lines LOVO, HCT116, and SW620 (Fig. S2). Further, *MNX1* expression correlated with the clinical stage as per the UALCAN database (Fig. 1F). Therefore, immunohistochemistry assays were used to detect MNX1 levels in tissues from different clinical stage CRC patients; consequently, *MNX1* levels correlated with clinical stage in CRC patients (Fig. 1G).

### *MNX1* knockdown inhibits the migration and invasion of CRC cells

**Figure 2:**
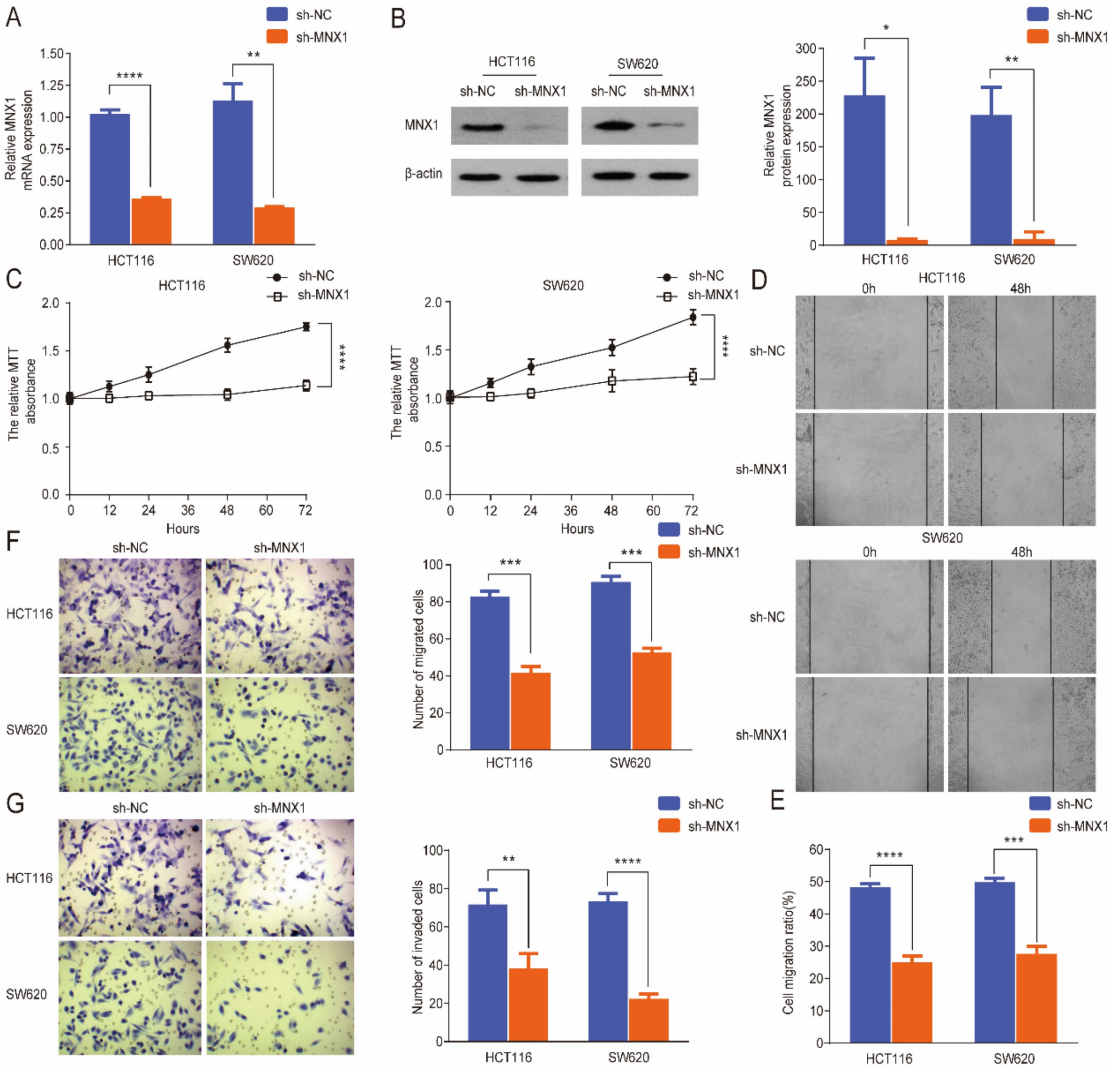
MNX1 knockdown affects the migration and invasion of colorectal cancer cells. (A, B) MNX1 mRNA and protein levels are downregulated in cell lines; (C) MTT analysis of the HCT116 and SW620 cells for the different groups; (D, E) Wound healing assays showed that MNX1 downregulation significantly reduced cell migration; (F, G) The results of the transwell assay revealed that MNX1 downregulation in HCT116 and SW620 cells decreased cell invasion and migration. Mean ± SD (n = 3 independent experiments). *P < 0.05, **P < 0.01, ***P < 0.001, ****P < 0.0001.

To further evaluate the effect of *MNX1* in colorectal cancer progression, a small hairpin RNA (shRNA) approach was used to knockdown *MNX1* in SW620 and HCT116 cell lines. qRT-PCR and Western blot results revealed that *MNX1* expression was significantly downregulated in the transfected cells after treatment (Fig. 2A, B). First, MTT assays showed that *MNX1* knockdown inhibited cancer cell growth (Fig. 2C). *MNX1* knockdown in HCT116 and SW620 cells substantially suppressed migration of CRC cells (Fig. 2D, E). Subsequently, we further analyzed the effects of *MNX1* on cell migration and invasion through Transwell analysis. As a result, HCT116 and SW620 cell migration and invasion were significantly lower than control groups (Fig. 2F, G).

### *MNX1* overexpression promotes the migration and invasion of CRC cells

**Figure 3:**
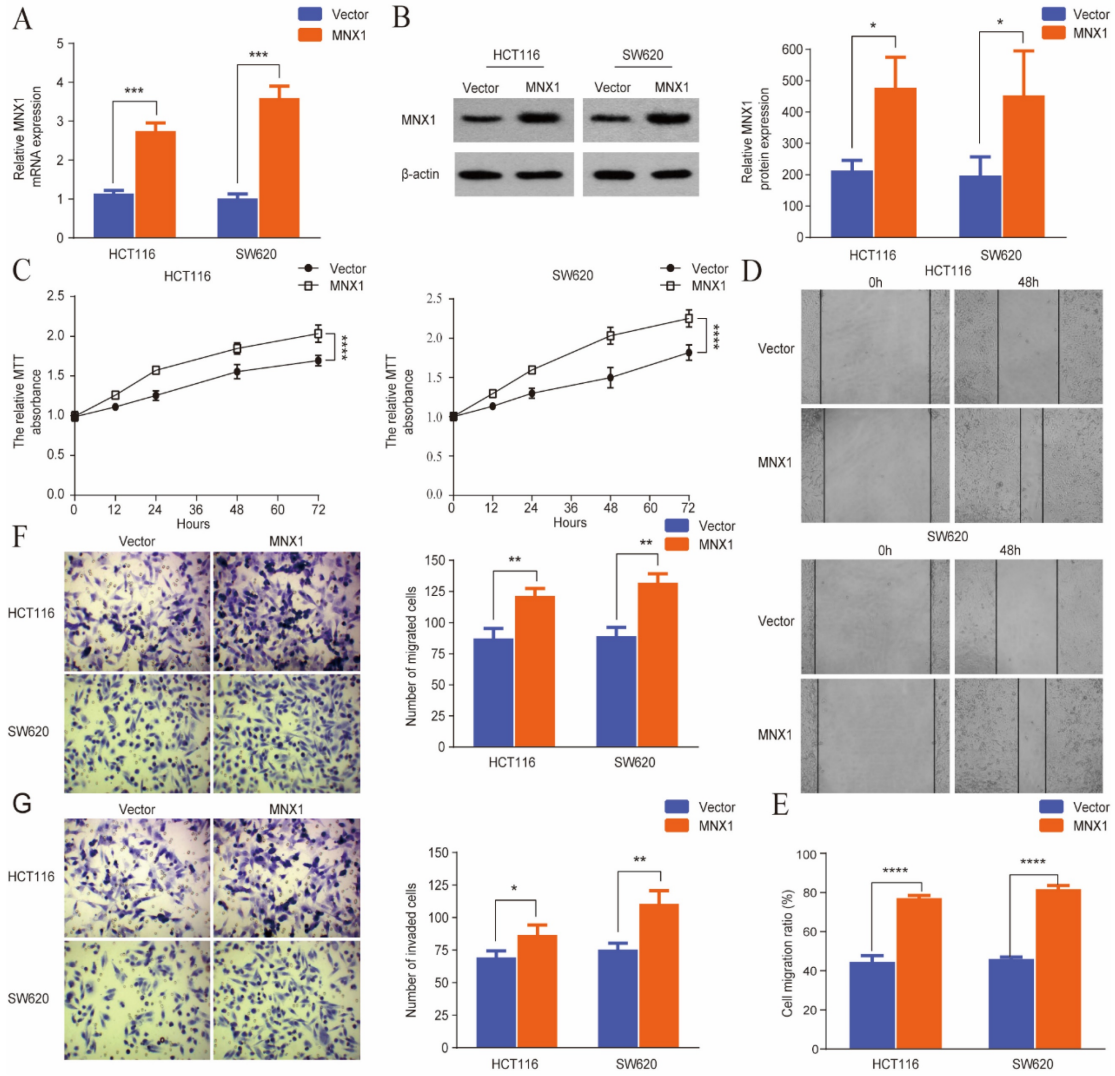
*MNX1* overexpression affects the migration and invasion of colorectal cancer cells. (A, B) Upregulated *MNX1* mRNA and protein levels in cell lines; (C) The MTT assay results showed the proliferation of the HCT116 and SW620 cells for different groups; (D, E) Wound healing assays revealed that* MNX1* upregulation substantially promoted cell migration; (F, G) The results of the transwell assay showed that *MNX1* upregulation in HCT116 and SW620 cells increased cell invasion and migration. Mean ± SD (n = 3 independent experiments). *P < 0.05, **P < 0.01, ***P < 0.001, ****P < 0.0001.

Stable *MNX1* overexpression models were constructed using HCT116 and SW620 cells to confirm the biological role of *MNX1* in CRC. The plasmid effectively upregulated *MNX1* expression, as confirmed by the qRT-PCR and Western blot results (Fig. 3A, B). First, in the MTT assay, *MNX1* overexpression increased HCT116 and SW620 cells (Fig. 3C). Through scratch wound assays, we noted that *MNX1* overexpression promoted cancer cell migration (Fig. 3D, E). Moreover, Transwell assays revealed that *MNX1* overexpression promoted cell migration and invasion compared to the control group (Fig. 3F, G). Summarily *MNX1* could influence the migratory and invasive capacity of colorectal cancer cells.

### *E2F4* is an upstream gene of *MNX1* in colorectal cancer

**Figure 4:**
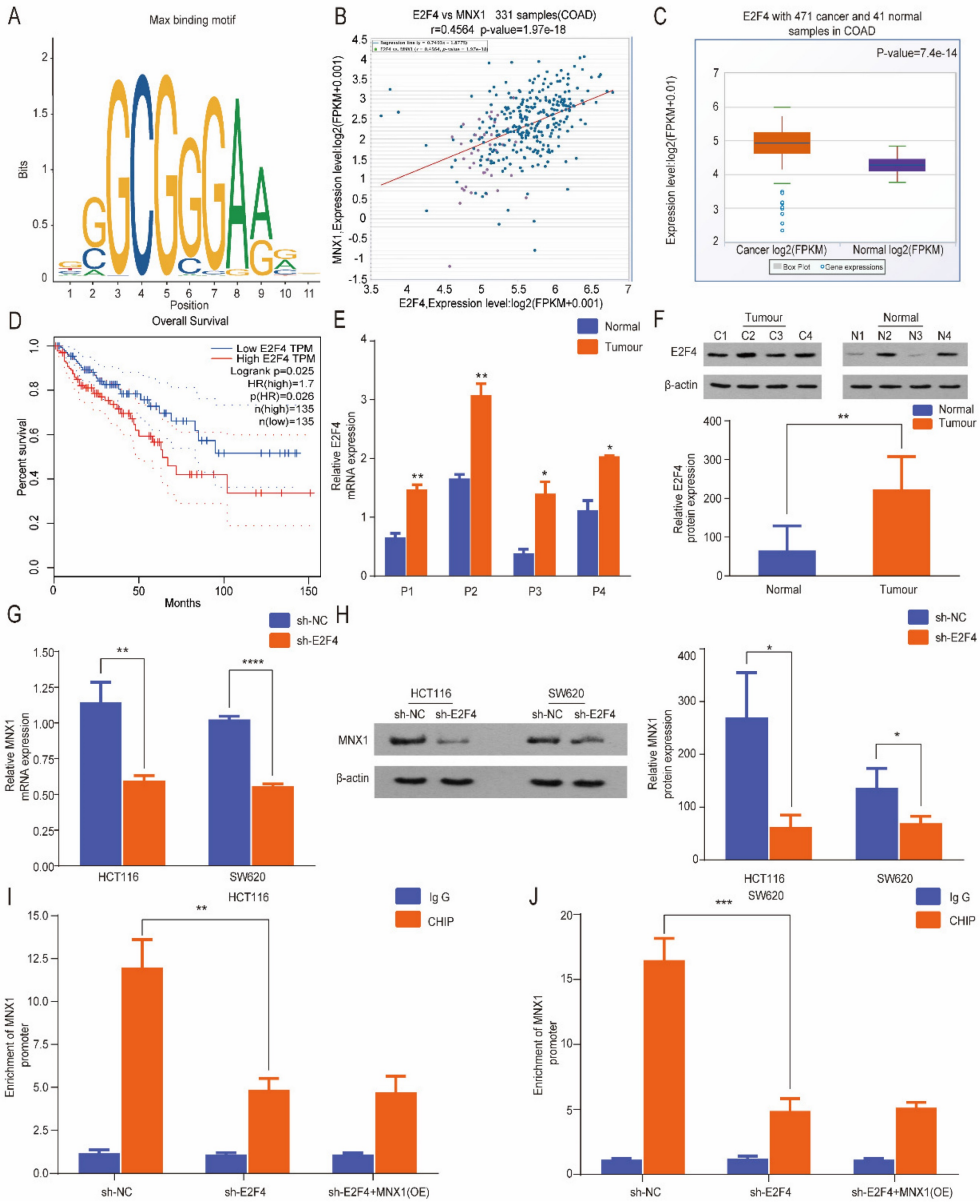
*MNX1* is a direct target of *E2F4*. (A) Predicted *E2F4*: MAX DNA-binding motif in the human *MNX1* promoter region; (B) The ChIPBase site indicated a positive correlation between *E2F4* and *MNX1*; (C, D) *E2F4* is upregulated in CC tissues compared to adjacent normal tissues in StarBase database (P=7.4e-14). Patients with high *E2F4* expression had poor disease-free survival (P=0.025); (E, F) *E2F4* expression determined by qRT-PCR and Western blot assays; (G, H) *E2F4* knockdown possibly promoted *MNX1* downregulation at the mRNA and protein levels; (I, J) ChIP assays revealed that *MNX1* promoter enrichment in the precipitates of *E2F4* antibody was lower than that in the control group. After *MNX1* delivery, *MNX1* promoter enrichment was not improved. Mean ± SD (n = 3 independent experiments). *P < 0.05, **P < 0.01, ***P < 0.001, ****P < 0.0001.

Based on the above experimental results, *MNX1* was implicated in CRC cell migration and invasion. Therefore, we further evaluated the underlying regulatory mechanisms of CRC. Furthermore, we investigated the upstream regulatory gene of *MNX1*. Based on the prediction of the RNAinter database, *MNX1* was a predicted target of *E2F4*, and transcription factors identify specific RNA sequences regulating gene expression and function. According to the JASPAR database, we predicted that *E2F4* could bind to the promoter regions of *MNX1* (Fig. 4A). Through ChIPBase database analysis, a positive correlation was found between *E2F4* and MNX1 (Fig. 4B). Meanwhile, the GEPIA and StarBase databases were analyzed and found predominant upregulation of *E2F4* in CRC, linked to a favorable overall survival curve (Fig. 4C, D). Therefore, we found *E2F4* upregulation in colorectal cancer tissues (Fig. 4E, F) and CRC cell lines (Fig. S3A, B). Furthermore, *MNX1* was dramatically reduced after the *E2F4* knockdown (Fig. 4G, H). ChIP assays revealed that *MNX1* promoter enrichment in the precipitates of the *E2F4* antibody was lower than that in the control group. After delivery of *MNX1*, the *MNX1* promoter enrichment was not improved (Fig. 4I, J). Based on the above results, we speculated that *E2F4* could effectively bind to the *MNX1* promoter.

### *E2F4* promotes the development of CRC by activating *MNX1*

**Figure 5:**
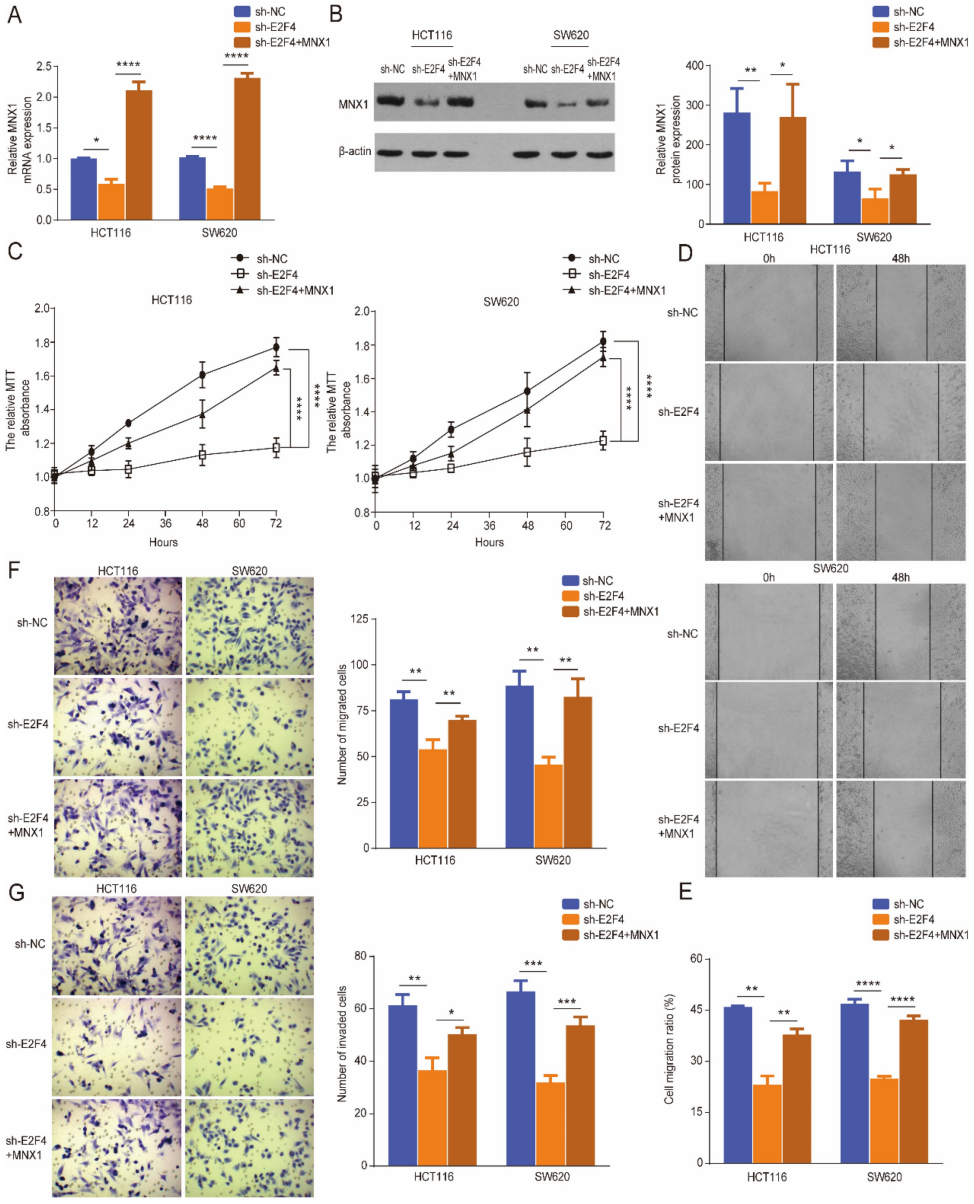
*MNX1* upregulation partially recovered the malignant phenotypes of sh-*E2F4* cells. (A, B) The mRNA and protein levels of *MNX1* were partially reversed when *MNX1* was upregulated in the sh-*E2F4* cells compared to the sh-*E2F4* cells alone; (C) The proliferative capacities were partially rescued after *MNX1* upregulation in sh-*E2F4* HCT116, and SW620 cells by MTT analysis; (D-G) The invasion and migration capacities have been improved after *MNX1* upregulation in sh-*E2F4* cells compared to sh-*E2F4* cells alone. Error bars represent the mean ± SD values of three independent experiments. *P < 0.05, **P < 0.01, ***P < 0.001, ****P < 0.0001, NS, No significance.

Rescue experiments were performed further to verify the regulatory role of *E2F4* in CRC. Western blotting and qRT-PCR results showed that *E2F4* knockdown downregulated *MNX1* expression in HCT116 and SW620 cell lines; however, these effects were reversed through *MNX1* overexpression (Fig. 5A, B). The MTT wound healing and Transwell assays revealed that *MNX1* overexpression partially reversed the decreased migration and invasion of HCT116 and SW620 cells caused by *E2F4* knockdown (Fig. 5B-G).

**Figure 6:**
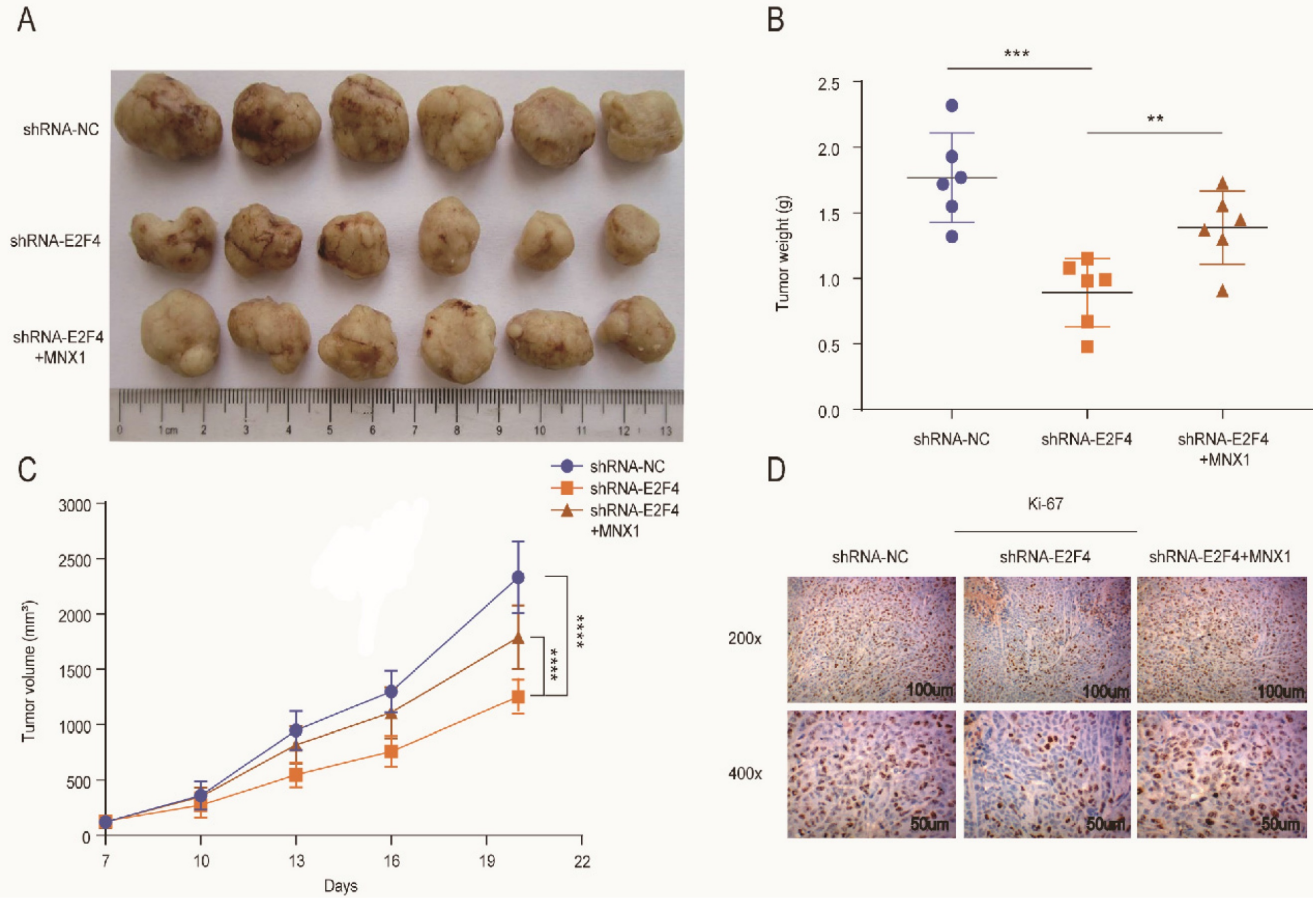
*E2F4* activates *MNX1* to facilitate the proliferation of CRC cells *in vivo*. (A) The xenograft tumours extracted from the nude mice; (B, C) The tumour weight and volumes of the mice in different groups are illustrated in the diagram; (D) Ki-67 expression in different groups is shown by Immunohistochemistry assay. Error bars represent the mean ± SD values of three independent experiments. **P < 0.01, ***P < 0.001, ****P < 0.0001.

**Figure 7:**
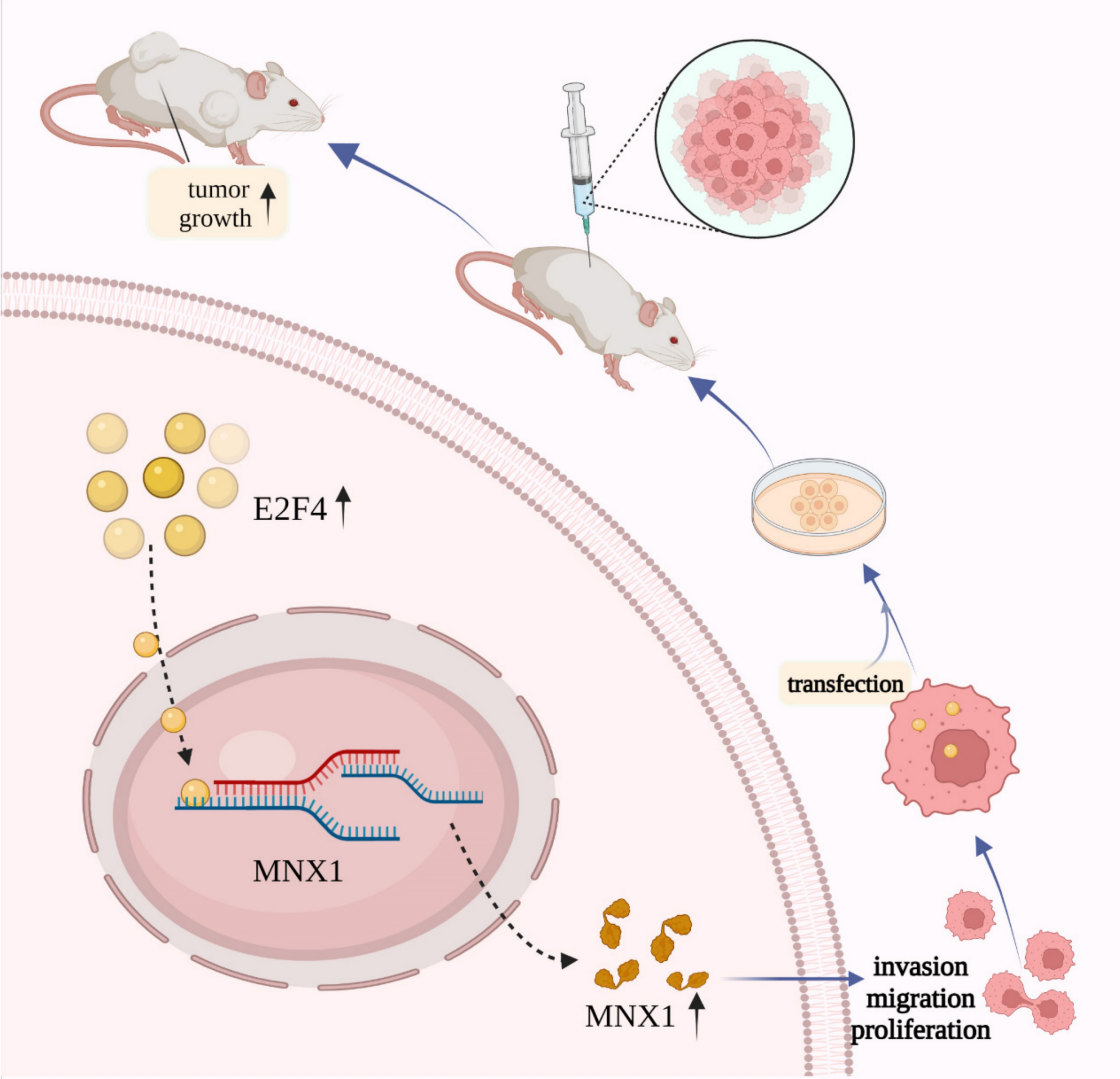
Schematic diagram of *E2F4*/*MNX1* pathway in colorectal cancer progression.

The xenograft mice models were used to explore *E2F4* and *MNX1* roles *in vivo*. shRNA-*E2F4*, shRNA-*E2F4*+*MNX1*, and shRNA-NC were successfully transfected using the HCT116 cells (Fig. 6A). The tumour weight and volume in the shRNA-*E2F4* group were the lowest, whereas the continuous delivery of *MNX1* overexpressed plasmids increased the tumour weight and volume (Fig. 6B, C). Furthermore, immunohistochemistry revealed that the number of positive cells for Ki-67 staining in the shRNA-*E2F4* group was substantially lower than in the other two groups (Fig. 6D). Therefore, we believe that *E2F4* activates *MNX1* transcription to promote the development of CRC (Fig 7).

## Discussion

Colorectal cancer has one of the highest cancer-related mortality rates in both sexes 15, 16. Although significant steps have been made in diagnosis and treatment, CRC prognosis is not promising. Therefore, there is a need for urgent research on the pathogenesis of this malignancy and the identification of novel indices for early diagnosis, therapy, and dynamic monitoring of colorectal cancer progression. Many genes are responsible for the occurrence and development of CRC, including NCOA5 17, FOXC2 18, and MNX1 19. Wu et al. reported that *MNX1* overexpression induces the proliferation of CRC cells. Nonetheless, the mechanism of *MNX1* upregulation and the comprehensive roles of *MNX1 in vivo* remain unclear 19.

*MNX1* is a member of the homeobox gene (*HOX*) *family*, and several homeobox genes are involved in the development of various malignant tumours 20. For instance, *SIX1* is upregulated in the cervical 21 and pancreatic cancers 22; *MNX1* correlates with multiple tumours 23-25. This work confirmed overexpressed* MNX1* in CRC tissues and cell lines by qRT-PCR and Western blotting, which corroborates with the bioinformatics analysis results in CRC.

Further, we analyzed the mechanism of *MNX1* upregulation and the roles of *MNX1* in CRC. In a bladder cancer study, *MNX1* regulated cell proliferation by targeting the G1-S transition *in vitro* and *in vivo* 26. In cervical cancer, *MNX1* promotes cancer cell proliferation, invasion, migration, and the progression of the cell cycle by regulating the transcription of *p21cip1* 27, 28. Here, overexpressed *MNX1* promoted proliferation, invasion, and migration of colorectal cancer cells, and downregulated levels yielded the opposite result. Based on the JASPAR and RNAinter databases, we speculated that as an essential upstream regulator, *E2F4* stimulates *MNX1* to promote CRC invasion and migration. We further confirmed this conclusion through chip assay. Current research indicates that the *E2F* family, including *E2F1* and *E2F4*, regulates cell cycle, tumorigenesis and progression of multiple tumours. 29, 30. As a transcription factor, *E2F* proteins regulate the expression of target genes by binding to specific sequences in the promoter of target genes. For instance, *E2F* often binds to the 5 '-TTTSSCGC-3' (S=C or G) sequence of the target gene promoter, expressed in several genes 31, 32. Ye et al. reported that *E2F1*-mediated abnormal expression of LncRNA *MNX1‐AS1* promotes colon adenocarcinoma progression 13. Moreover, recent research indicates that the transcription factor *E2F4* activates downstream genes in many cancers 33, 34. However, for the first time, we report that *E2F4* is involved in tumour progression by regulating downstream *MNX1* expression, providing new insight into the mechanistic research and treatment of CRC.

Western blot and qRT‐PCR showed *E2F4* upregulation in CRC tissues and cell lines. Functional assays revealed that *E2F4* downregulation correspondingly downregulated *MNX1* expression in CRC cells. The function of the *E2F4-MNX1* axis *in vivo* was further explored, and we constructed the shRNA-E2F4, shRNA-E2F4+MNX1, and shRNA-NC mice models, respectively. The tumour weight and volume in the shRNA-E2F4 group were the lowest, whereas the continuous delivery of overexpressed *MNX1* plasmids increased the tumour weight and volume. Therefore, we confirmed that *E2F4* has a strong correlation with *MNX1*.

In conclusion, we proposed that *E2F4* may act as a transcriptional regulator, activating *MNX1* to induce colorectal cancer development. These findings consider the *E2F4/MNX1* feedback loop a novel potential diagnostic biomarker of CRC. However, additional research on this perspective is essential in the future.

## Supplementary Material

Supplementary figures and tables.

## Figures and Tables

**Table 1:** Primers for quantitative real-time PCR (qRT-PCR).

Homo/Gene	Primer sequences 5′-3′	
E2F4	F	*CACCACCAAGTTCGTGTCCC*
	R	*GCGTACAGCTAGGGTGTCA*
MNX1	F	*GAGTGCGTGTGAGAAGAACC*
	R	*CAGTTTGAACGCTCGTGACA*
β-actin	F	*ACCCTGAAGTACCCCATCGAG*
	R	*AGCACAGCCTGGATAGCAAC*

**Table 2:** Primers for Chromatin Immunoprecipitation (ChIP)-PCR.

Homo/Gene	Primer sequences 5′-3′	
MNX1	F	CTCCAGGGACCAACCAAGT
	R	CAACGGGGAGTGGATACTC
GAPDH	F	TACTAGCGGTTTTACGGGCG
	R	TCGAACAGGAGGAGCAGAGAGCGA
