# Supplementary material for: A Positive Feedback Loop of E2F4-Mediated Activation of MNX1 Regulates Tumour Progression in Colorectal Cancer
Source: J Cancer. 2023 Sep 4;14(14):2739–50. doi: 10.7150/jca.86718 (PMC10539396; doi:10.7150/jca.86718)
Supplement: Supplementary file 1 — Supplementary figures and tables. [file jcav14p2739s1.zip › supplementary/raw data/Figure 4/Predicted binding sites.docx]

Predicted binding sites from JASPAR

| Model ID | Model name | Score | Start | End | Strand | predicted site sequence |
| --- | --- | --- | --- | --- | --- | --- |
| MA0470.1 | E2F4 | 15.123 | 2778 | 2788 | 1 | GGGCGGGAGGG |
| MA0470.1 | E2F4 | 7.678 | 2786 | 2796 | 1 | GGGCAGGAGGG |
