# Supplementary material for: A Positive Feedback Loop of E2F4-Mediated Activation of MNX1 Regulates Tumour Progression in Colorectal Cancer
Source: J Cancer. 2023 Sep 4;14(14):2739–50. doi: 10.7150/jca.86718 (PMC10539396; doi:10.7150/jca.86718)

**Supplementary Figure S1:**（a）Data from GDS4382: the expression level of MNX1 in tumour tissues and corresponding normal tissues (n=17). Data are shown as the mean ± SD. **P* < 0.05.


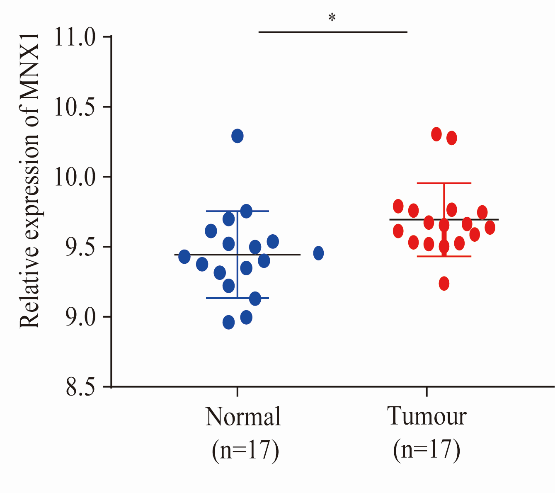


**Supplementary Figure S2:****Expression of MNX1 in cell lines:** (a) Relative expression of MNX1 in cell lines by qRT-PCR.(b) Relative expression of MNX1 in cell lines by Western blot assays. Data are shown as the mean ± SD. **P* < 0.05, ***P* < 0.01, ****P* < 0.001.


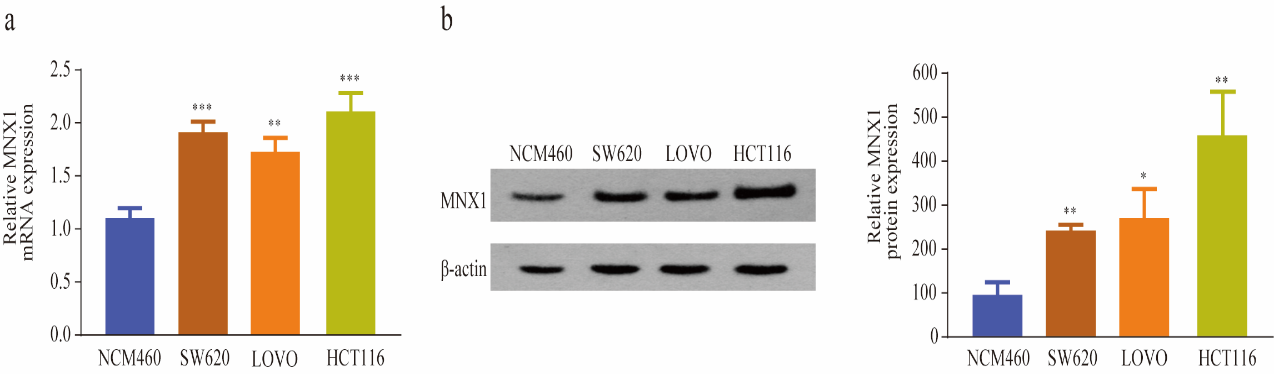


**Supplementary Figure S3: Expression of E2F4 in cell lines:** (a) Relative expression of E2F4 in cell lines by qRT-PCR.(b) Relative expression of E2F4 in cell lines by Western blot assays. Data are shown as the mean ± SD. **P* < 0.05, ***P* < 0.01, ****P* < 0.001.


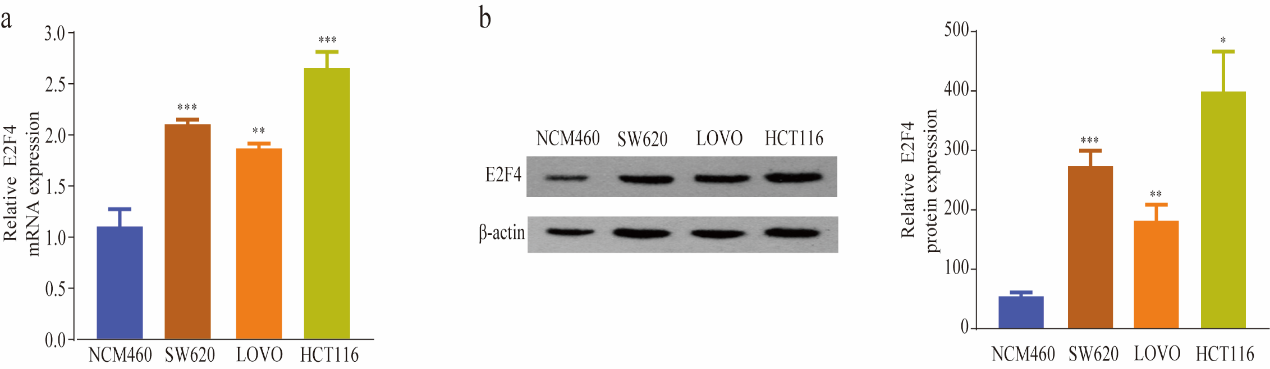

Supplement: Supplementary file 1 — Supplementary figures and tables. [file jcav14p2739s1.zip › supplementary/Supplementary material(F).docx]
