# Supplementary material for: A Positive Feedback Loop of E2F4-Mediated Activation of MNX1 Regulates Tumour Progression in Colorectal Cancer
Source: J Cancer. 2023 Sep 4;14(14):2739–50. doi: 10.7150/jca.86718 (PMC10539396; doi:10.7150/jca.86718)
Supplement: Supplementary file 1 — Supplementary figures and tables. [file jcav14p2739s1.zip › supplementary/supplementary table .docx]

Supplementary table 1: The relationship between the comorbidities and MNX1

| **Complications** | **Number of cases** | **MNX1 Expression** | | ***P*-value** |
| --- | --- | --- | --- | --- |
|  |  | **Low** | **High** |  |
| **Hypertension**  Female  Male |  |  |  |  |
|  | 2 | 1 | 1 | 1.000 |
|  | 4 | 1 | 3 |  |
| **Type 2 Diabetes Mellitus**  Female  Male |  |  |  |  |
|  | 1 | 0 | 1 | 1.000 |
|  | 3 | 1 | 2 |  |
| **Coronary Heart Disease**  Female  Male |  |  |  |  |
|  | 1 | 0 | 1 | 1.000 |
|  | 2 | 1 | 1 |  |

Supplementary table 2: The relationship between the comorbidities and E2F4

| **Complications** | **Number of cases** | **E2F4 Expression** | | ***P*-value** |
| --- | --- | --- | --- | --- |
|  |  | **Low** | **High** |  |
| **Hypertension**  Female  Male |  |  |  |  |
|  | 2 | 1 | 1 | 1.000 |
|  | 3 | 1 | 2 |  |
| **Type 2 Diabetes Mellitus**  Female  Male |  |  |  |  |
|  | 1 | 0 | 1 | 1.000 |
|  | 3 | 1 | 2 |  |
| **Coronary Heart Disease**  Female  Male |  |  |  |  |
|  | 1 | 0 | 1 | 1.000 |
|  | 3 | 1 | 2 |  |

Supplementary table 3: The relationship between the clinical parameters and MNX1

| **Characteristics** | **Number of cases** | **MNX1 Expression** | | ***P*-value** |
| --- | --- | --- | --- | --- |
|  |  | **Low** | **High** |  |
| **Gender**  Female  Male |  |  |  |  |
|  | 3 | 2 | 1 | 0.523 |
|  | 9 | 3 | 6 |  |
| **Clinical Stage**  Ⅰ-Ⅱ  Ⅲ-Ⅳ |  |  |  |  |
|  | 8 | 5 | 3 | 0.081 |
|  | 4 | 0 | 4 |  |
| **T classification**  T1-T2  T3-T4 |  |  |  |  |
|  | 1 | 0 | 1 | 1.000 |
|  | 11 | 5 | 6 |  |
| **N classification** |  |  |  |  |
| N0 | 8 | 5 | 3 | 0.081 |
| N1-N2 | 4 | 0 | 4 |  |
| **M classification** |  |  |  |  |
| M0 | 11 | 5 | 6 | 1.000 |
| M1 | 1 | 0 | 1 |  |

Supplementary table 4: The relationship between the clinical parameters and E2F4

| **Characteristics** | **Number of cases** | **E2F4 Expression** | | ***P*-value** |
| --- | --- | --- | --- | --- |
|  |  | **Low** | **High** |  |
| **Gender**  Female  Male |  |  |  |  |
|  | 3 | 2 | 1 | 0.236 |
|  | 9 | 2 | 7 |  |
| **Clinical Stage**  Ⅰ-Ⅱ  Ⅲ-Ⅳ |  |  |  |  |
|  | 8 | 4 | 4 | 0.208 |
|  | 4 | 0 | 4 |  |
| **T classification**  T1-T2  T3-T4 |  |  |  |  |
|  | 1 | 0 | 1 | 1.000 |
|  | 11 | 4 | 7 |  |
| **N classification** |  |  |  |  |
| N0 | 8 | 4 | 4 | 0.208 |
| N1-N2 | 4 | 0 | 4 |  |
| **M classification** |  |  |  |  |
| M0 | 11 | 4 | 7 | 1.000 |
| M1 | 1 | 0 | 1 |  |
