# Supplementary figures and images for: A Positive Feedback Loop of E2F4-Mediated Activation of MNX1 Regulates Tumour Progression in Colorectal Cancer (part 2 of 2)
Source: J Cancer. 2023 Sep 4;14(14):2739–50. doi: 10.7150/jca.86718 (PMC10539396; doi:10.7150/jca.86718)

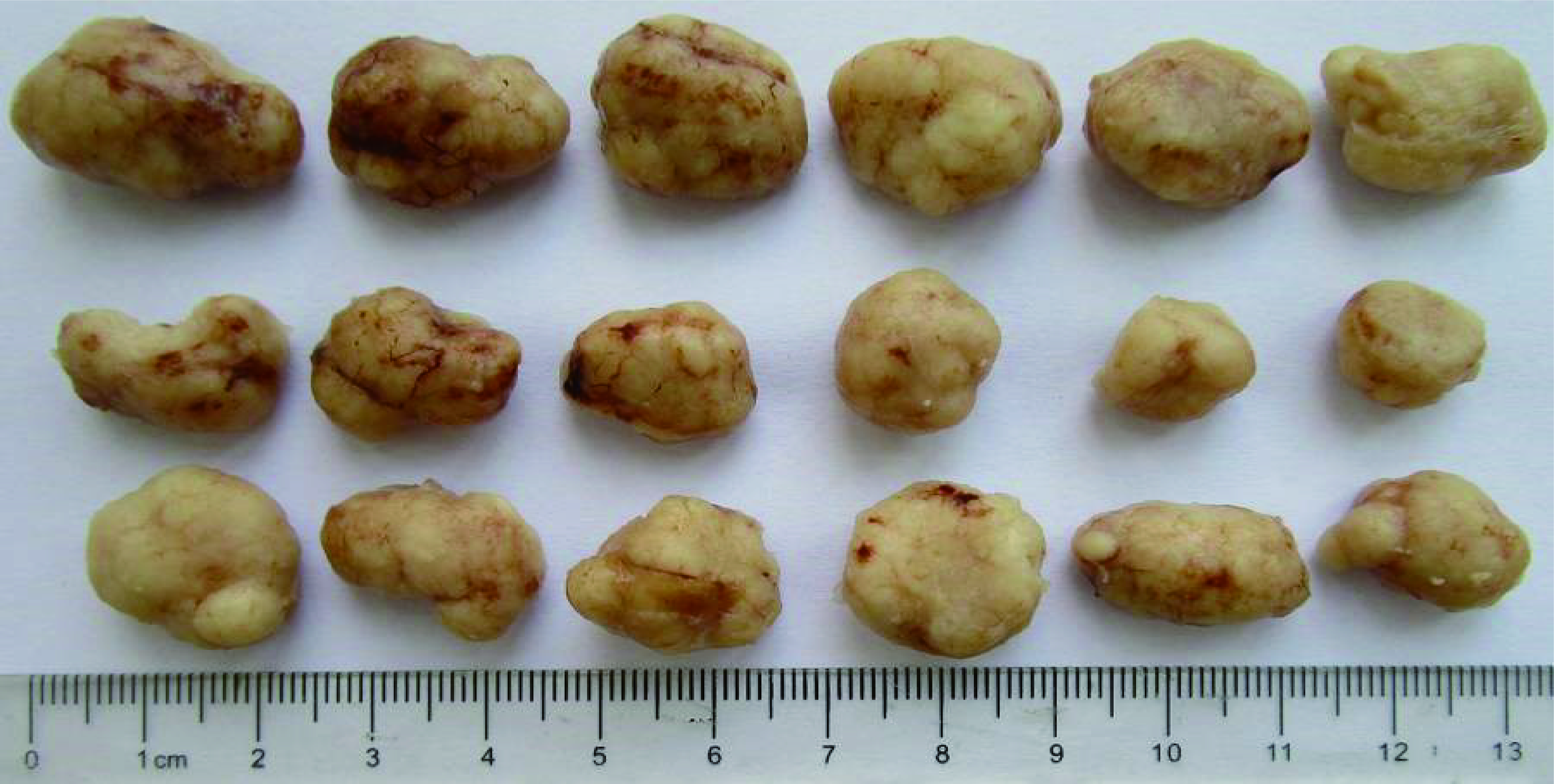

Supplement: Supplementary file 1 — Supplementary figures and tables. [file jcav14p2739s1.zip › supplementary/raw data/Figure 6/Mouse.tif]

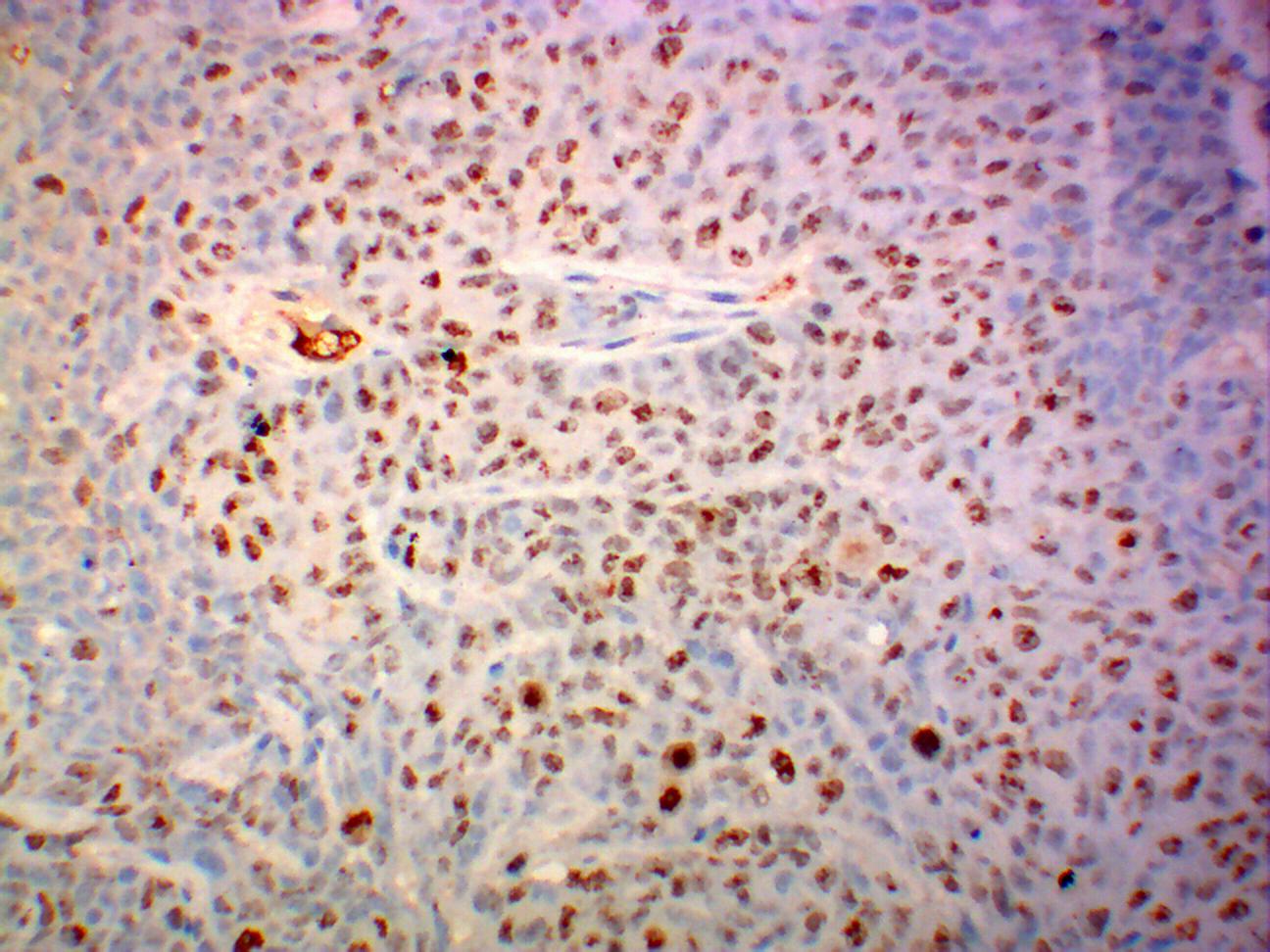

Supplement: Supplementary file 1 — Supplementary figures and tables. [file jcav14p2739s1.zip › supplementary/raw data/Figure 6/NC (1-1).JPG]

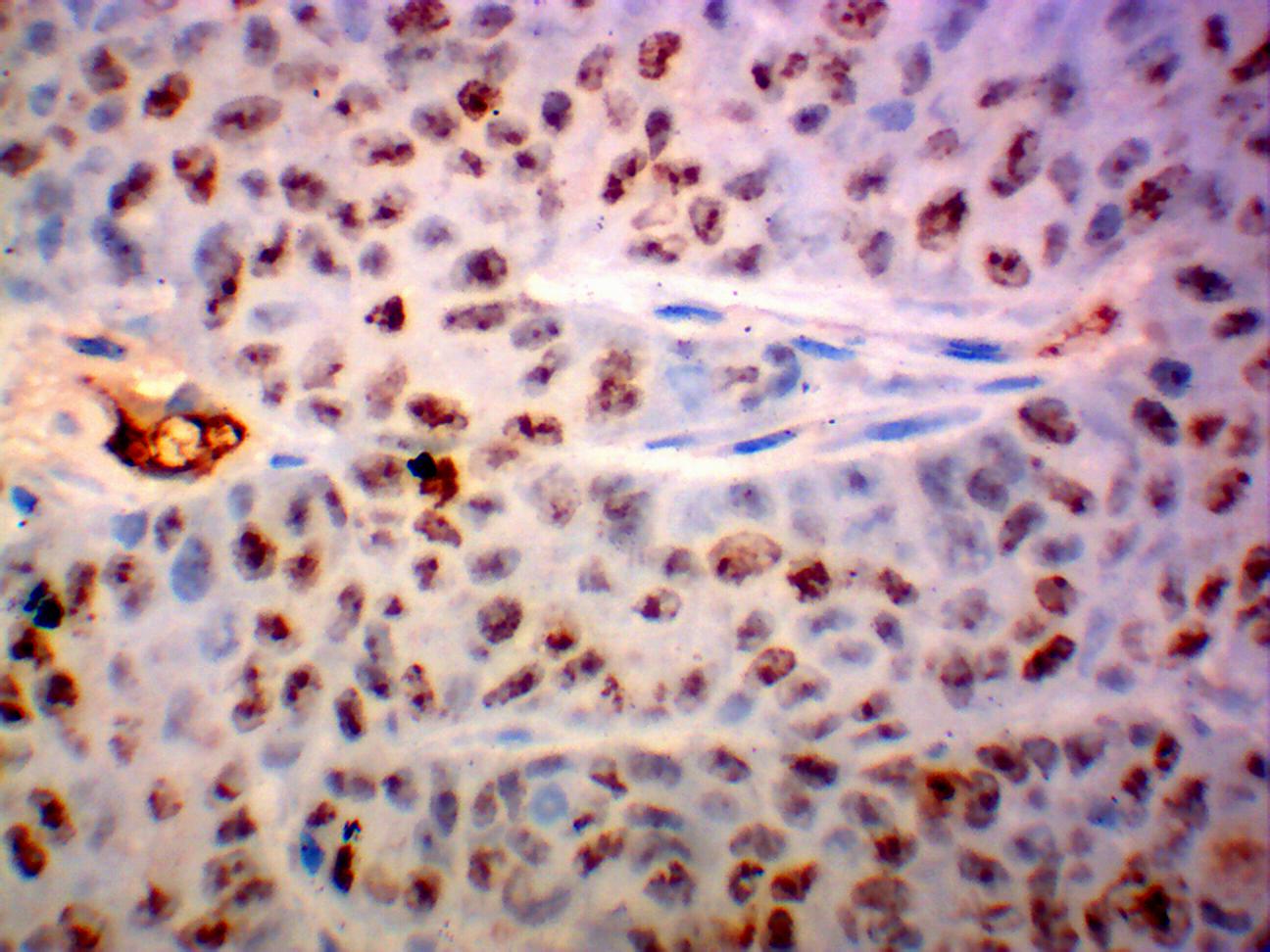

Supplement: Supplementary file 1 — Supplementary figures and tables. [file jcav14p2739s1.zip › supplementary/raw data/Figure 6/NC(1-2).JPG]

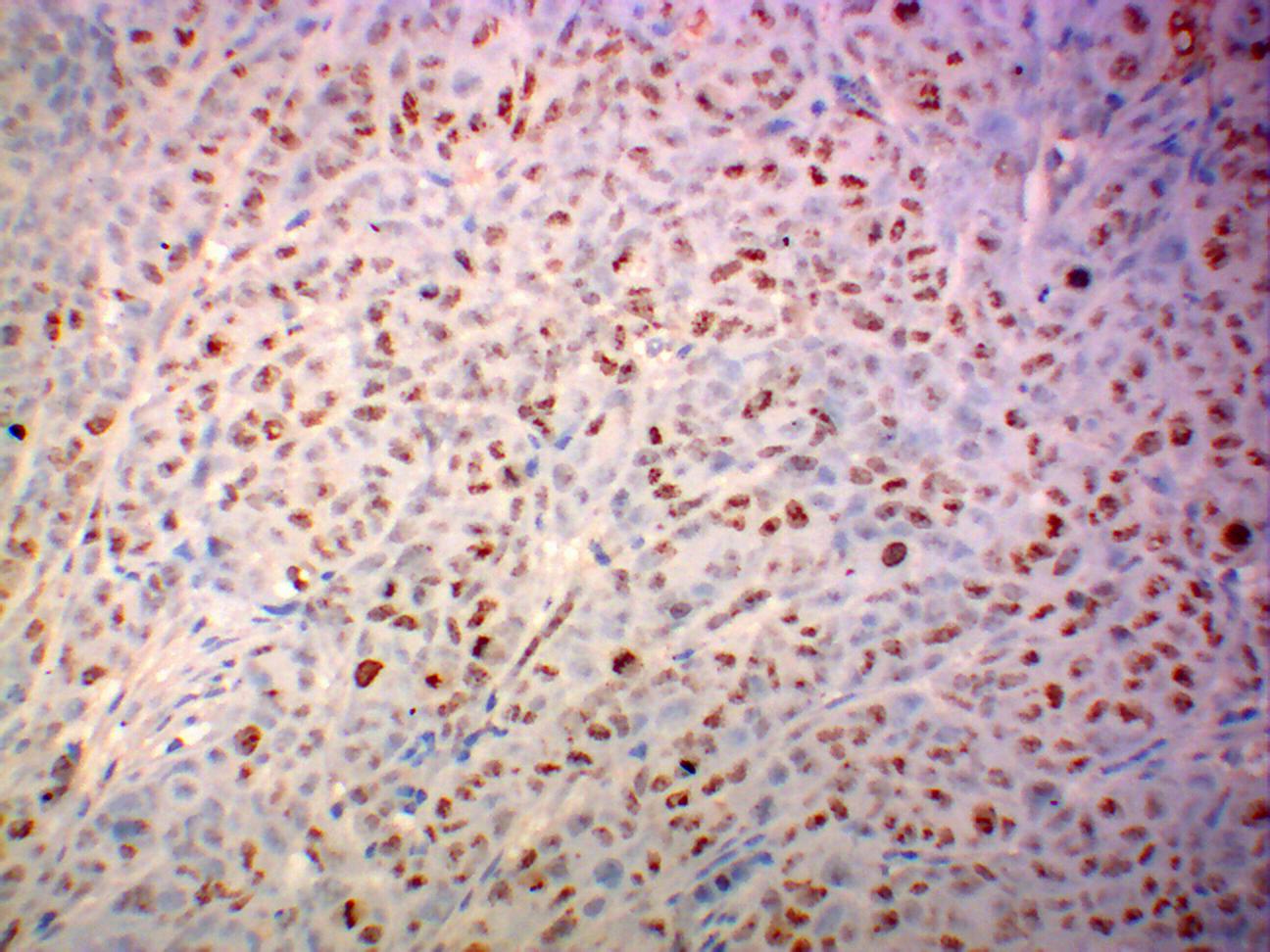

Supplement: Supplementary file 1 — Supplementary figures and tables. [file jcav14p2739s1.zip › supplementary/raw data/Figure 6/NC(2-1).JPG]

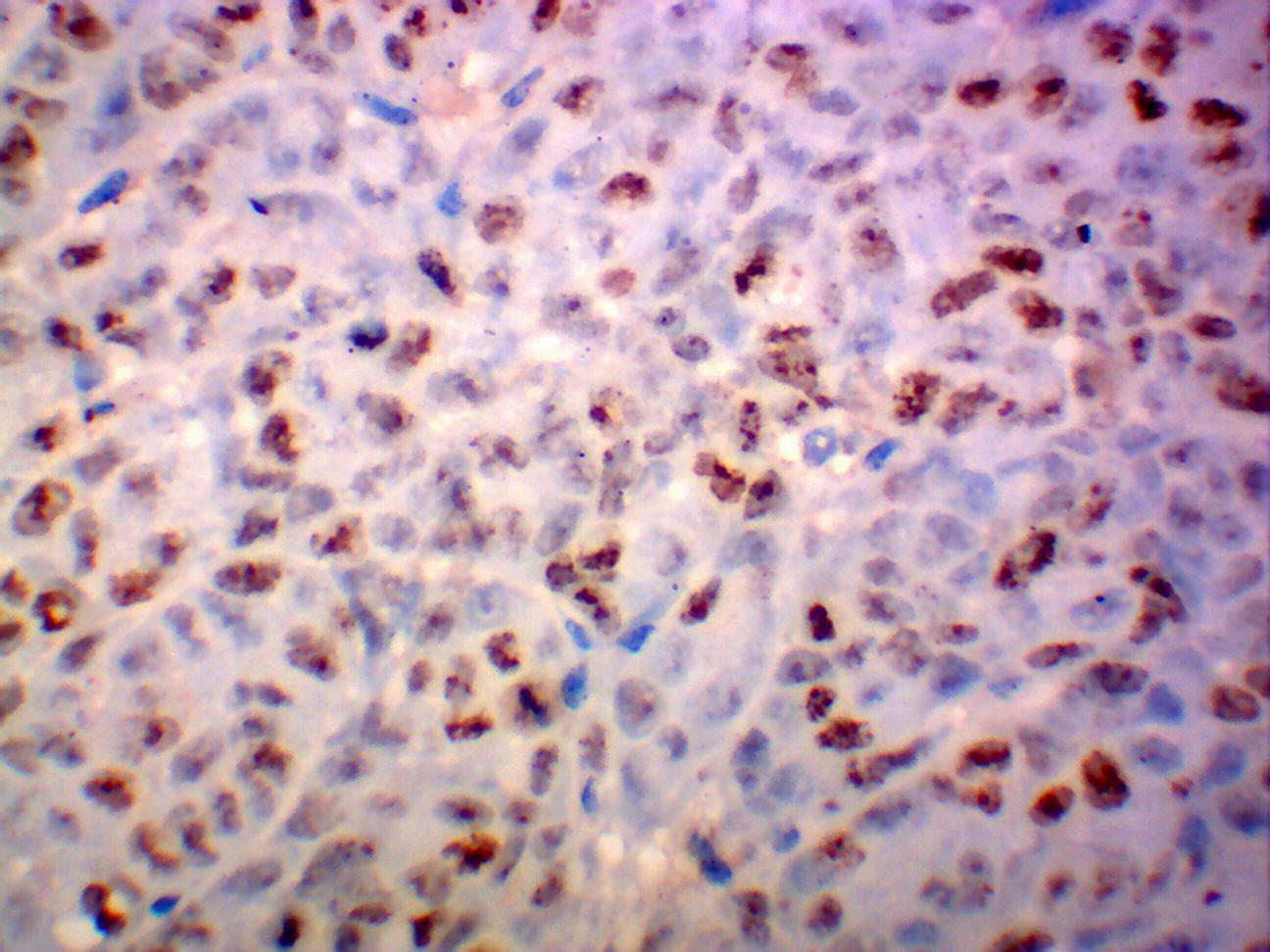

Supplement: Supplementary file 1 — Supplementary figures and tables. [file jcav14p2739s1.zip › supplementary/raw data/Figure 6/NC(2-2).JPG]

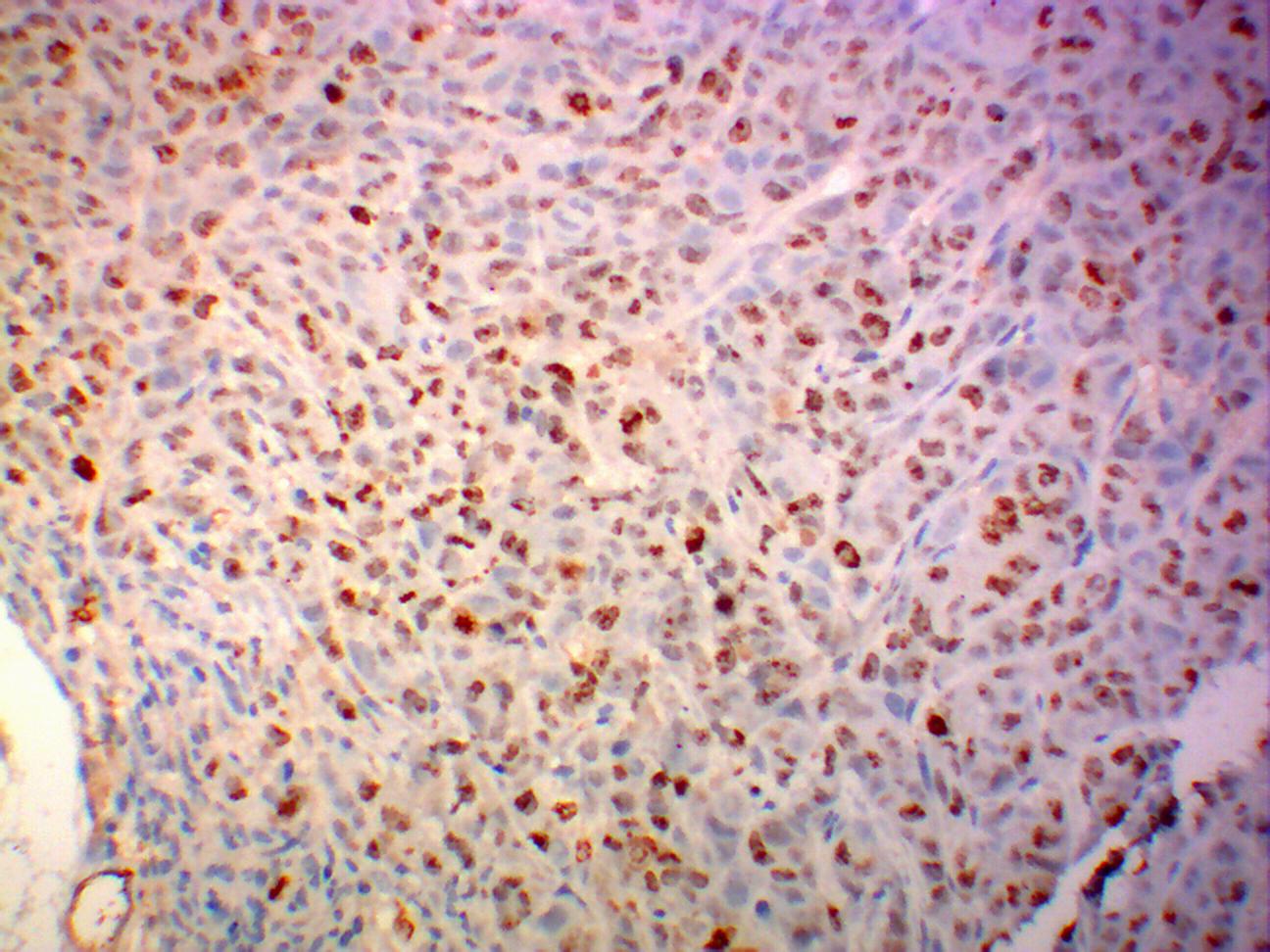

Supplement: Supplementary file 1 — Supplementary figures and tables. [file jcav14p2739s1.zip › supplementary/raw data/Figure 6/NC(3-1).JPG]

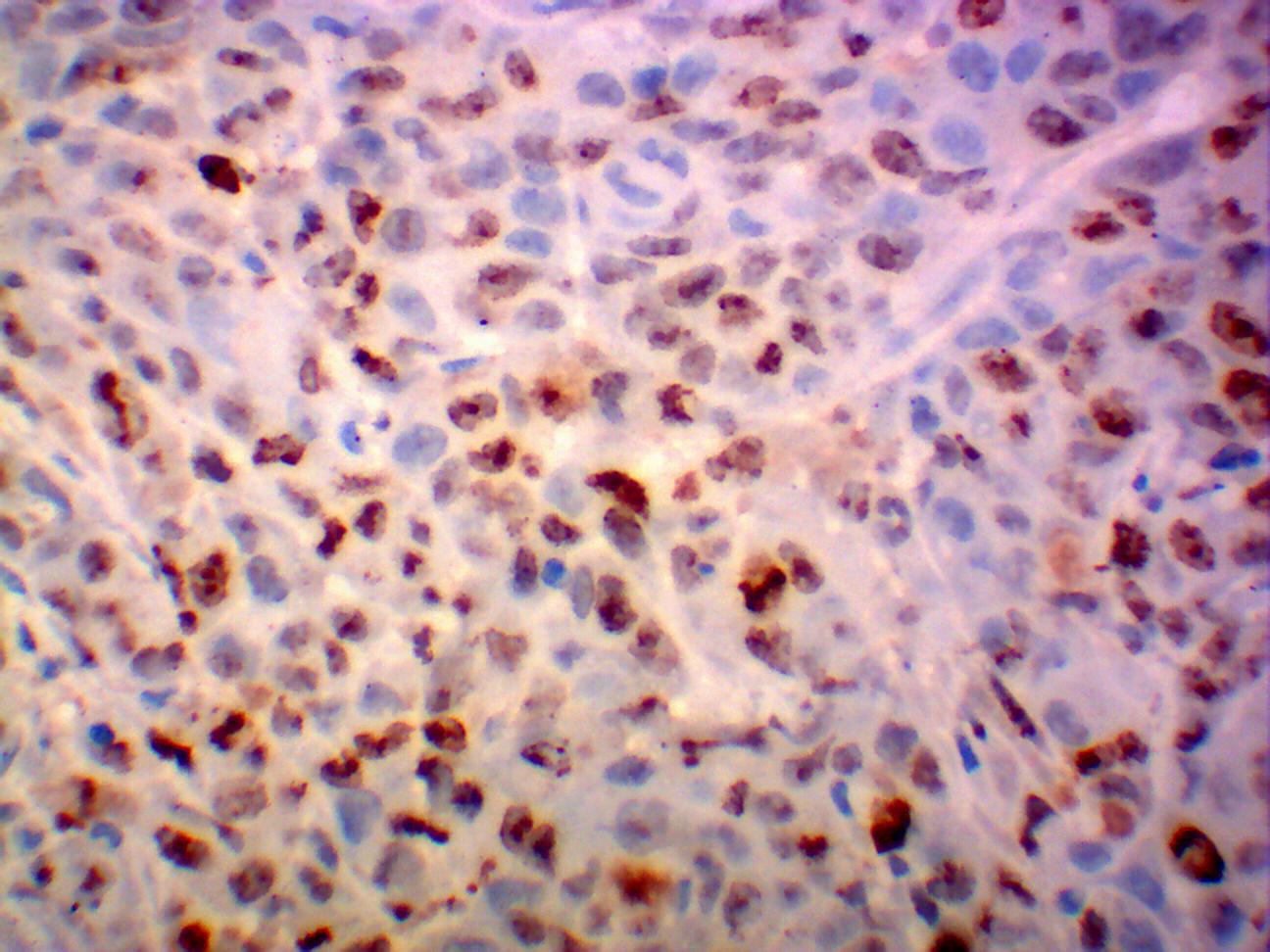

Supplement: Supplementary file 1 — Supplementary figures and tables. [file jcav14p2739s1.zip › supplementary/raw data/Figure 6/NC(3-2).JPG]
