# Supplementary figures and images for: A Positive Feedback Loop of E2F4-Mediated Activation of MNX1 Regulates Tumour Progression in Colorectal Cancer (part 1 of 2)
Source: J Cancer. 2023 Sep 4;14(14):2739–50. doi: 10.7150/jca.86718 (PMC10539396; doi:10.7150/jca.86718)

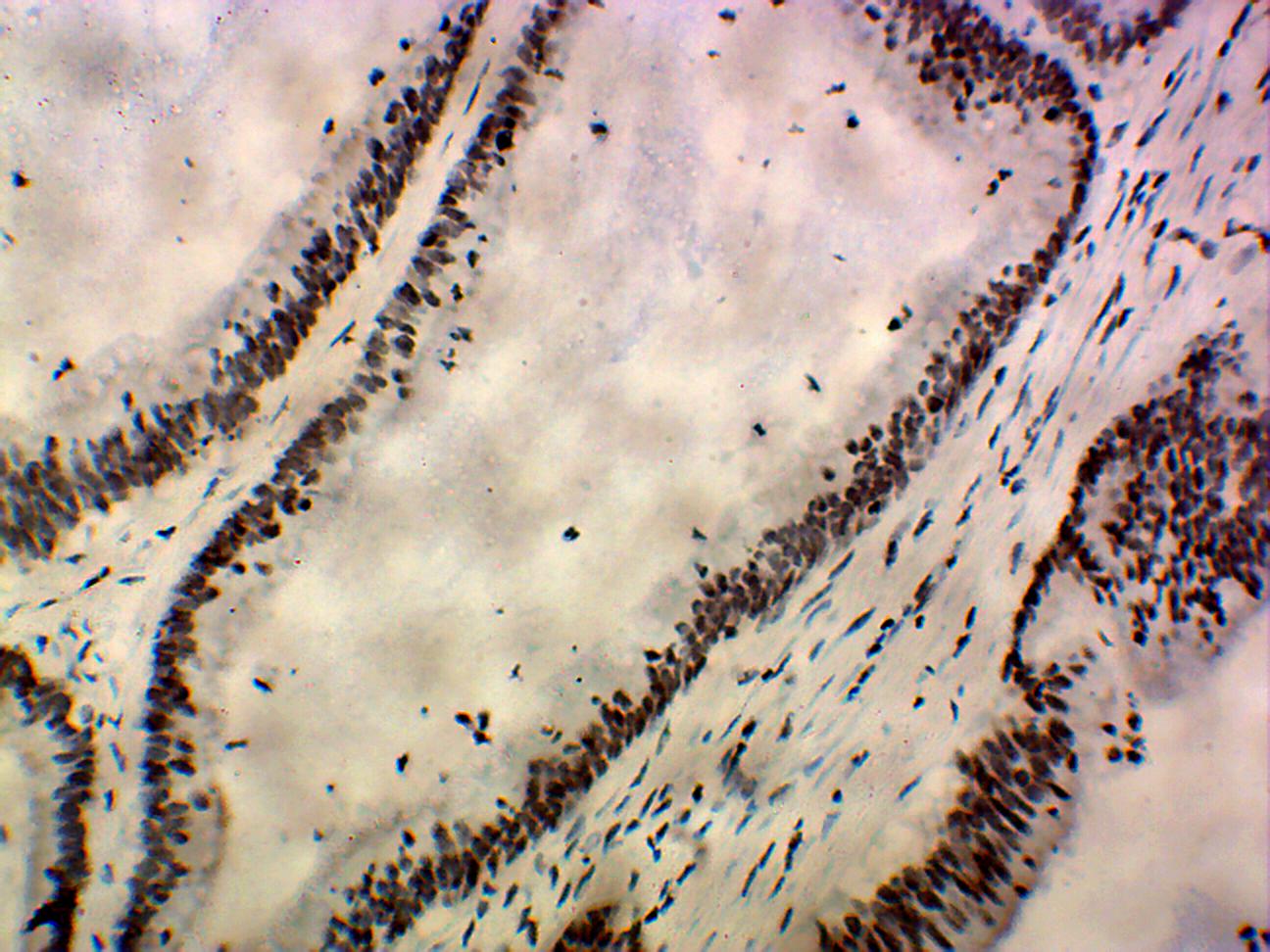

Supplement: Supplementary file 1 — Supplementary figures and tables. [file jcav14p2739s1.zip › supplementary/raw data/Figure 1/ca-3(1).JPG]

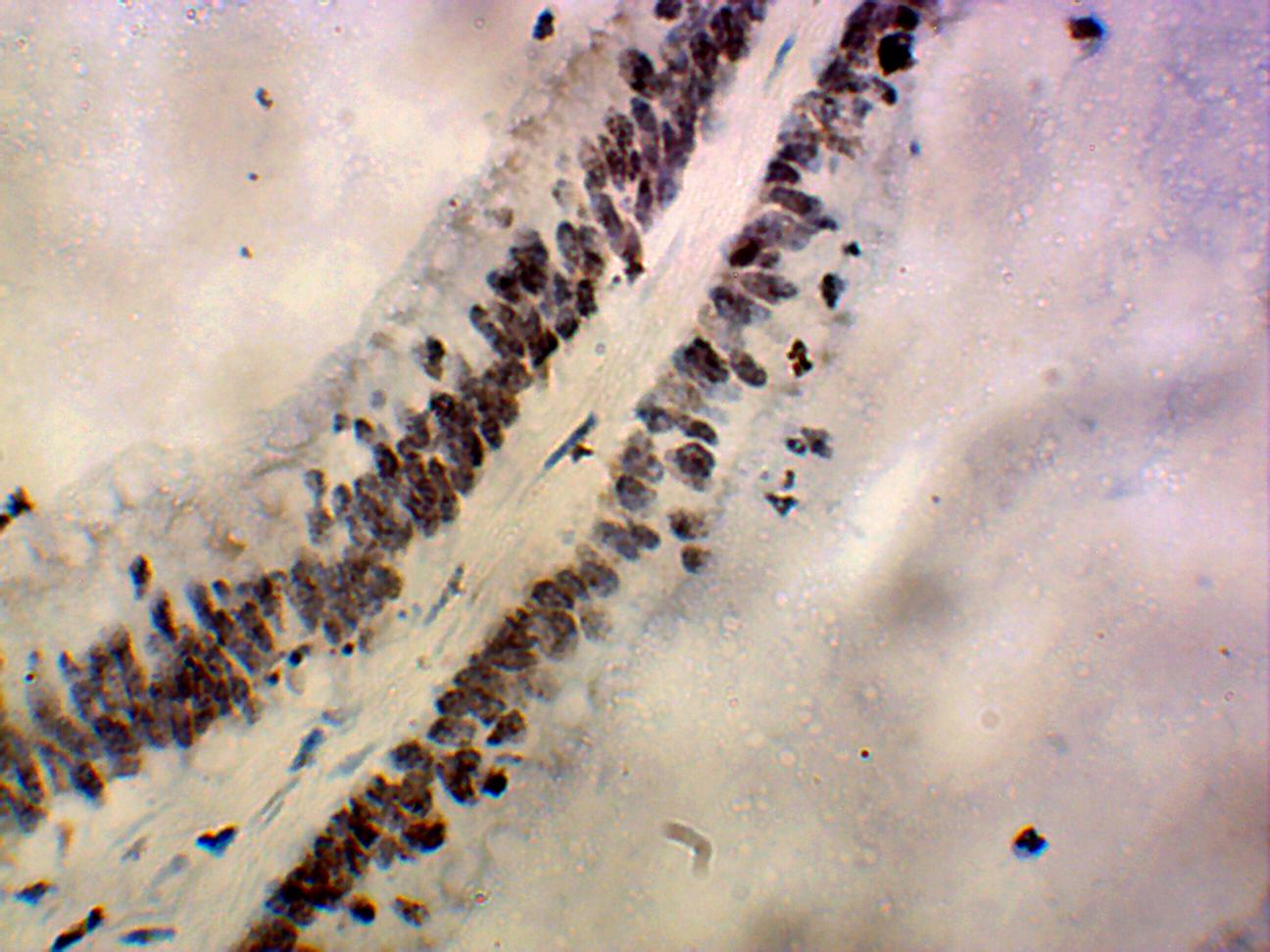

Supplement: Supplementary file 1 — Supplementary figures and tables. [file jcav14p2739s1.zip › supplementary/raw data/Figure 1/ca-3(2).JPG]

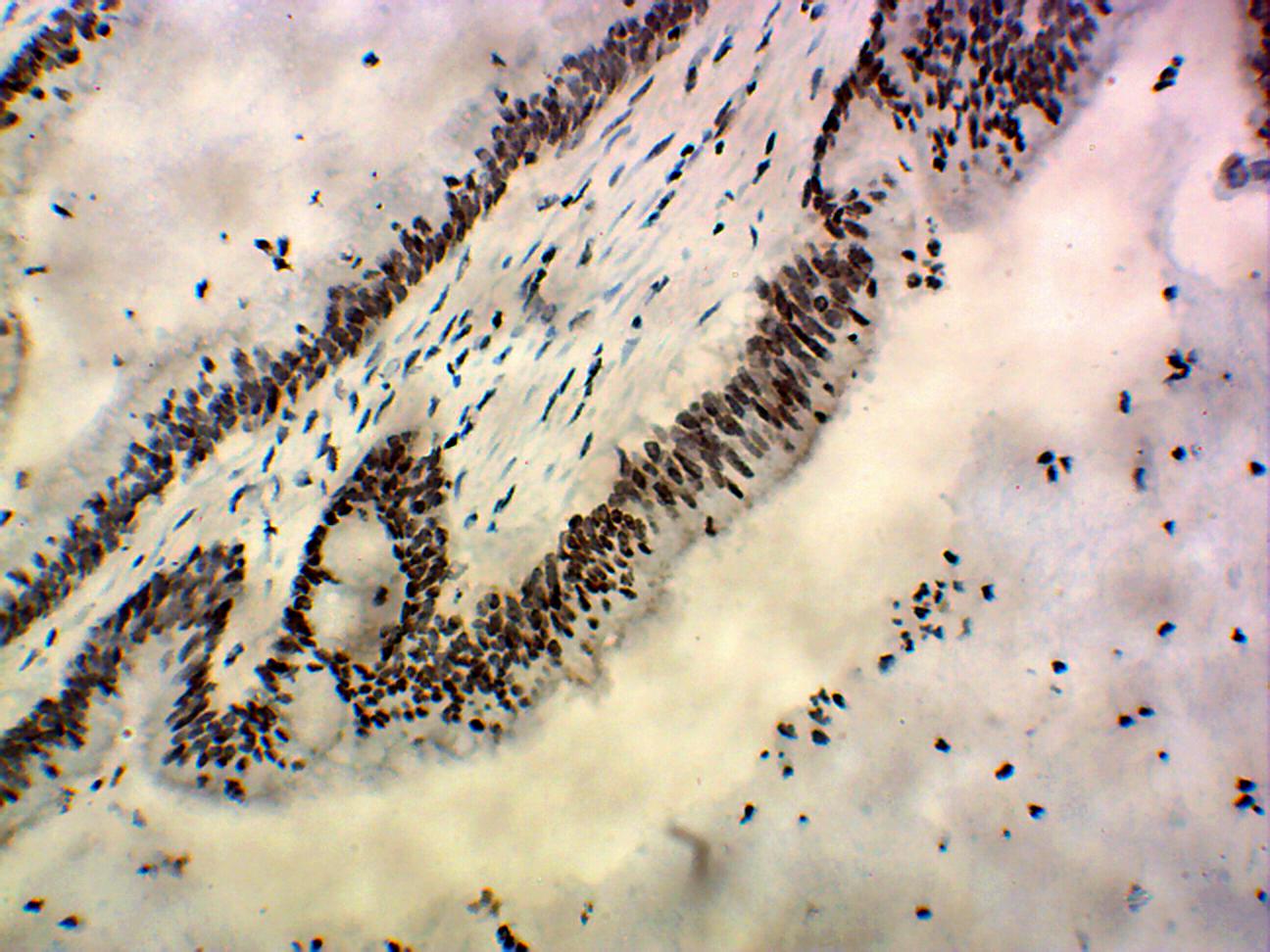

Supplement: Supplementary file 1 — Supplementary figures and tables. [file jcav14p2739s1.zip › supplementary/raw data/Figure 1/ca-4(1).JPG]

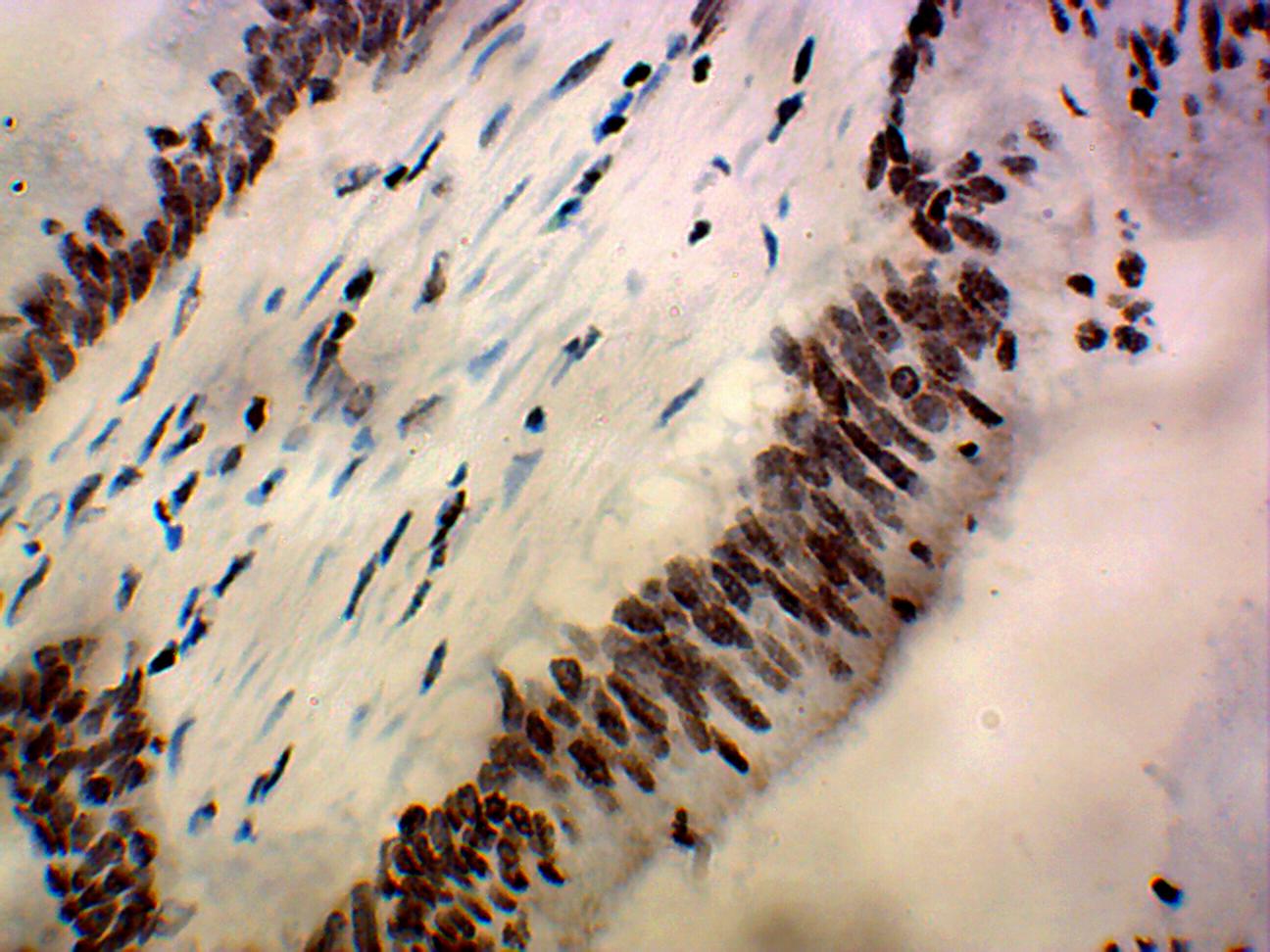

Supplement: Supplementary file 1 — Supplementary figures and tables. [file jcav14p2739s1.zip › supplementary/raw data/Figure 1/ca-4(2).JPG]

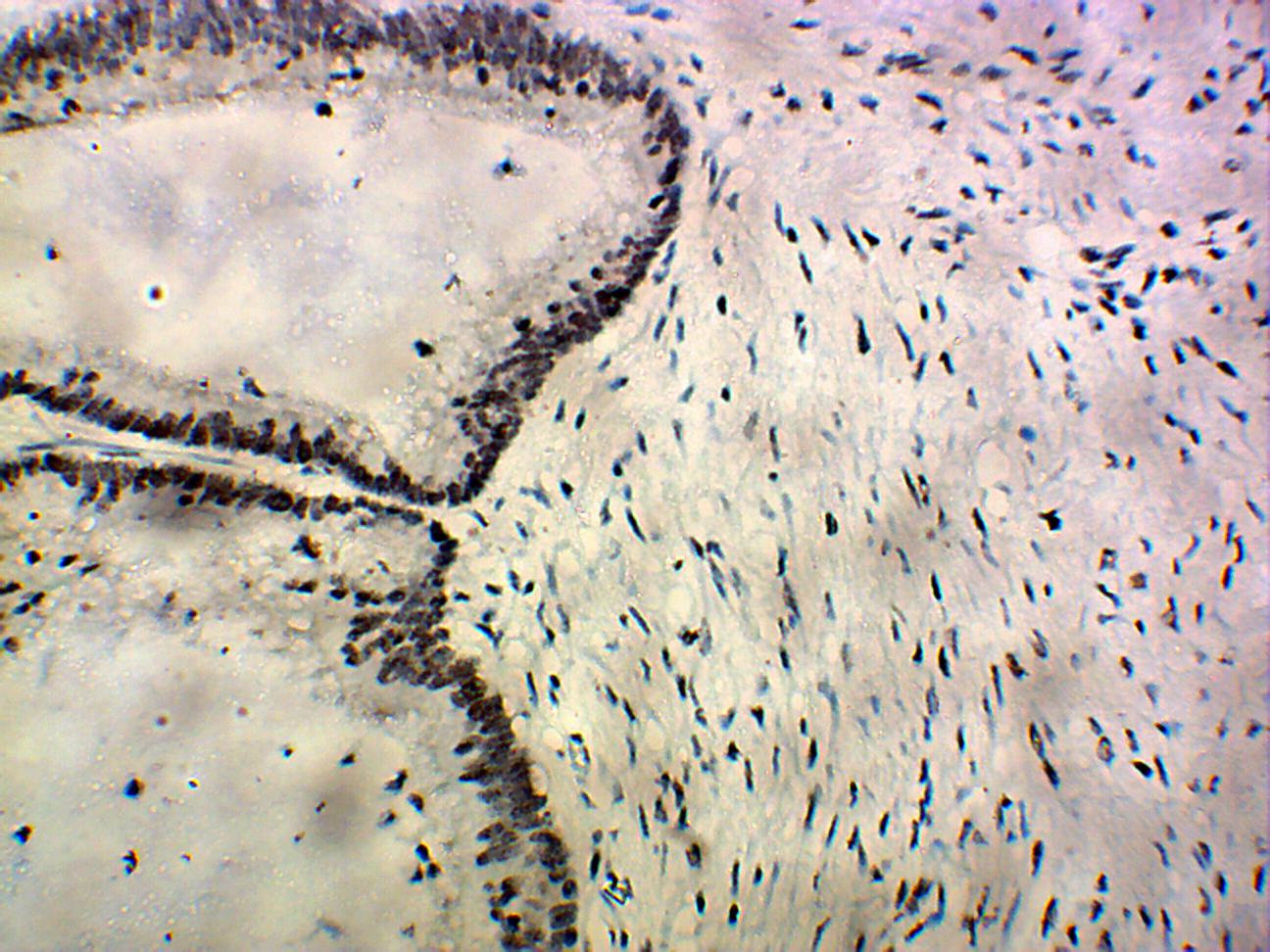

Supplement: Supplementary file 1 — Supplementary figures and tables. [file jcav14p2739s1.zip › supplementary/raw data/Figure 1/ca-5(1).JPG]

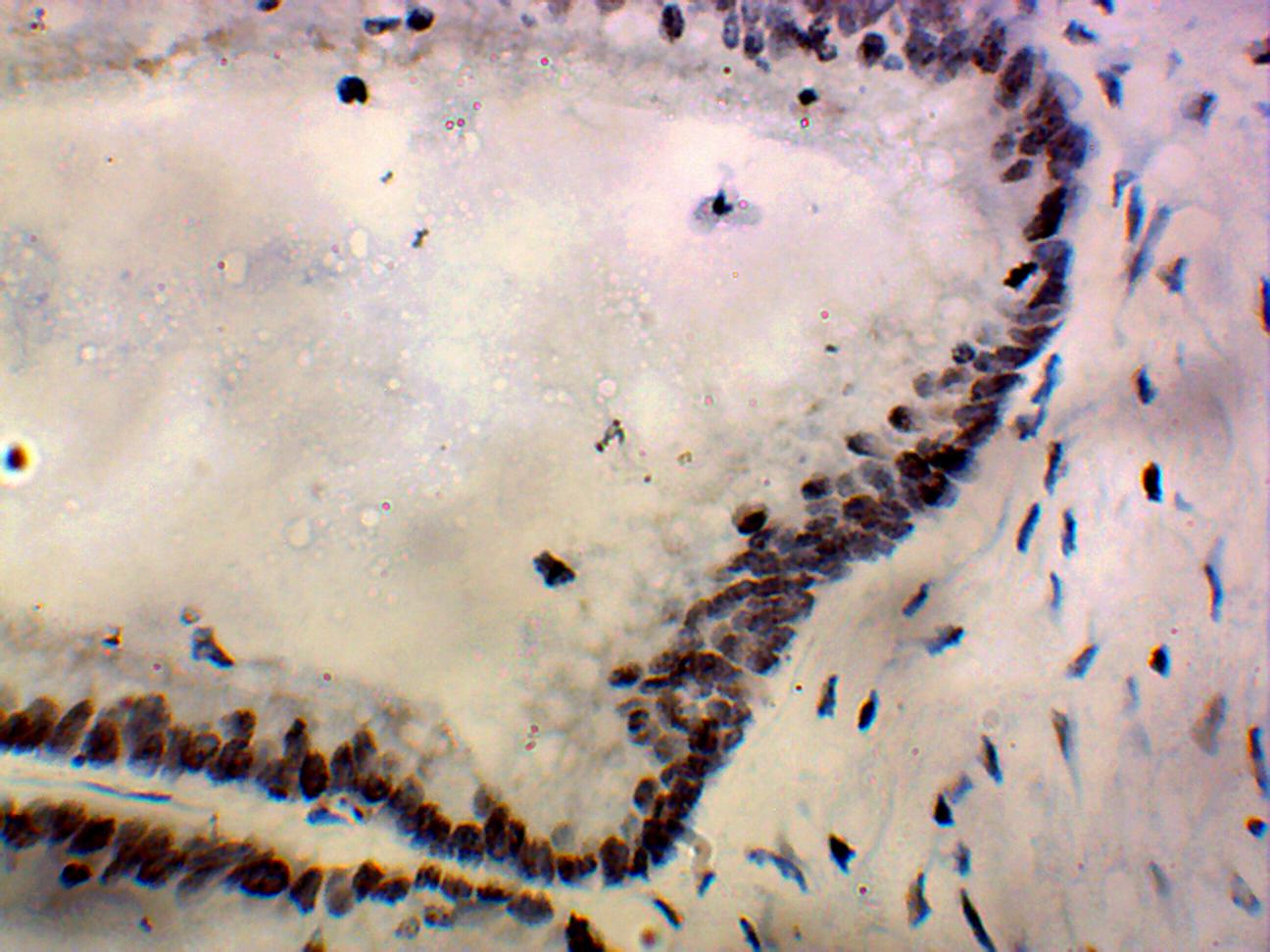

Supplement: Supplementary file 1 — Supplementary figures and tables. [file jcav14p2739s1.zip › supplementary/raw data/Figure 1/ca-5(2).JPG]

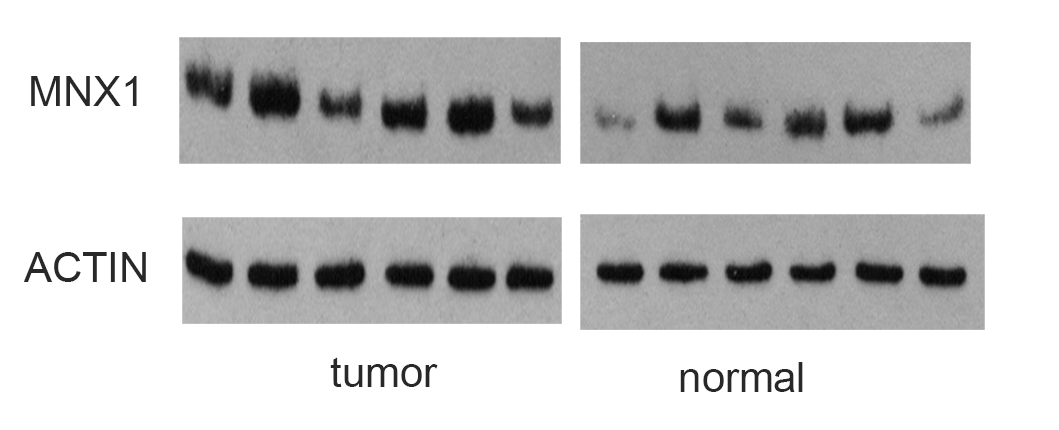

Supplement: Supplementary file 1 — Supplementary figures and tables. [file jcav14p2739s1.zip › supplementary/raw data/Figure 1/f1e repeat.tif]

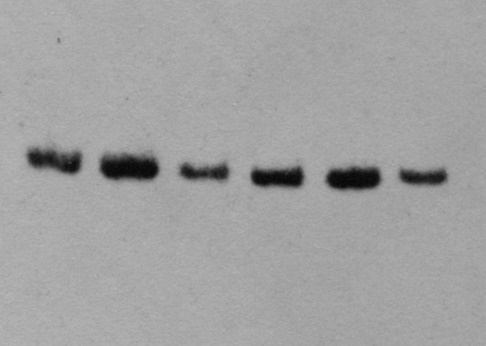

Supplement: Supplementary file 1 — Supplementary figures and tables. [file jcav14p2739s1.zip › supplementary/raw data/Figure 1/f1e-1.tif]

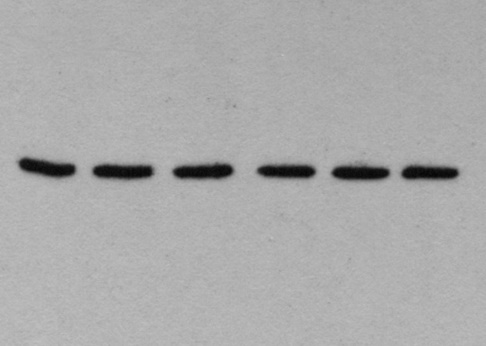

Supplement: Supplementary file 1 — Supplementary figures and tables. [file jcav14p2739s1.zip › supplementary/raw data/Figure 1/f1e-1b.tif]

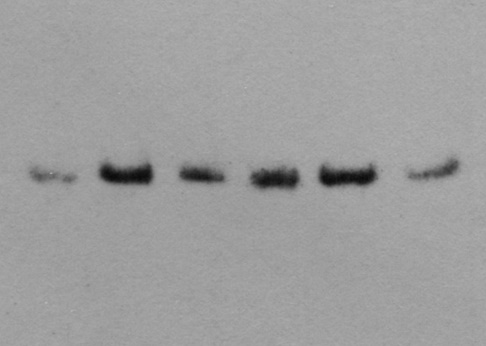

Supplement: Supplementary file 1 — Supplementary figures and tables. [file jcav14p2739s1.zip › supplementary/raw data/Figure 1/f1e-2.tif]

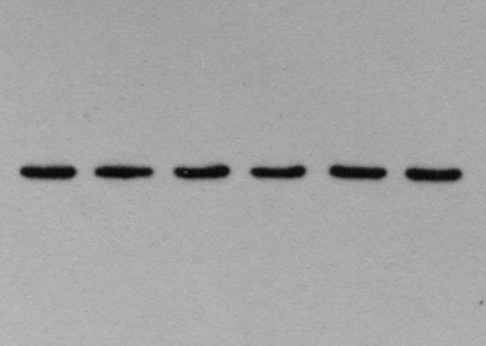

Supplement: Supplementary file 1 — Supplementary figures and tables. [file jcav14p2739s1.zip › supplementary/raw data/Figure 1/f1e-2b.tif]

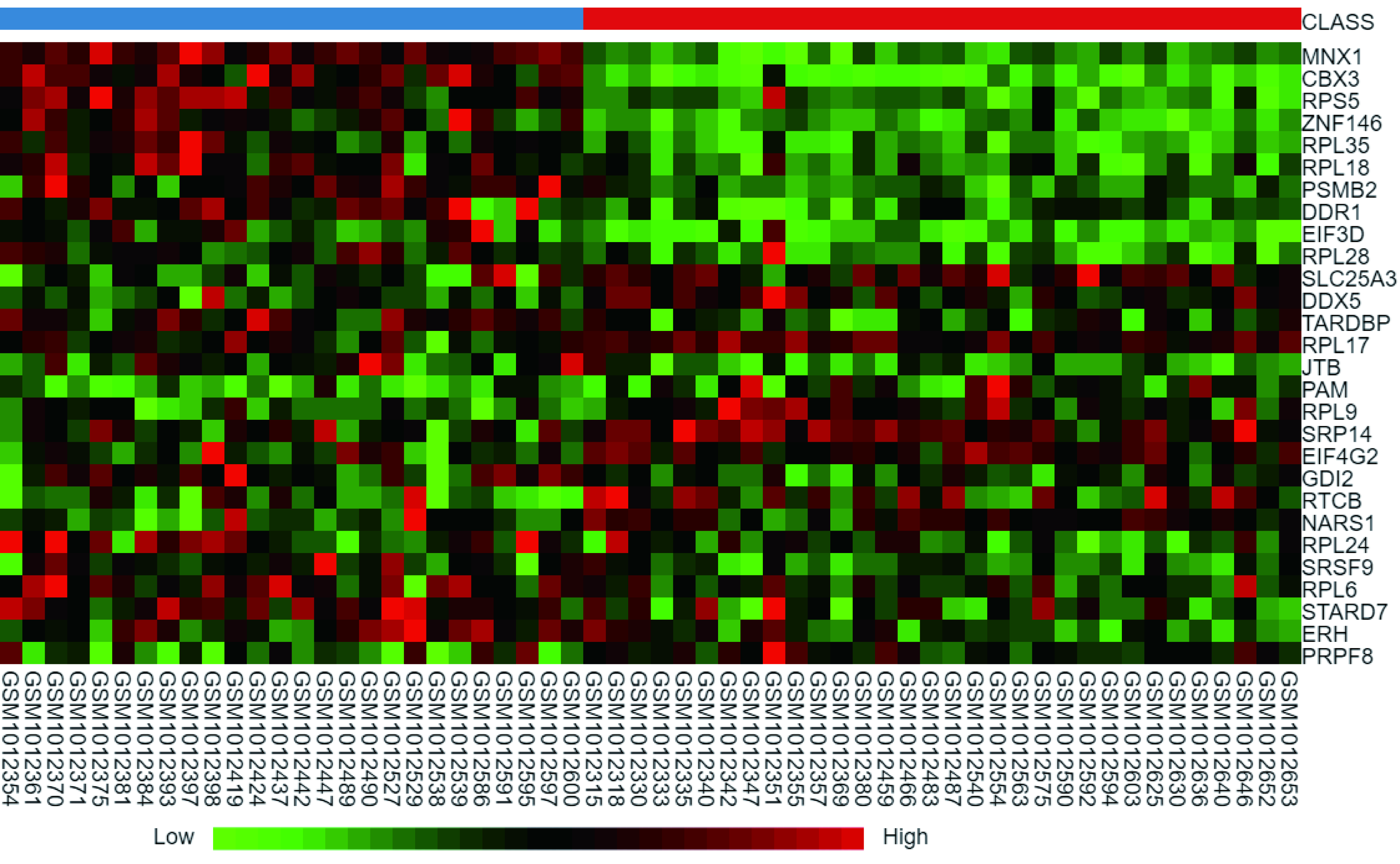

Supplement: Supplementary file 1 — Supplementary figures and tables. [file jcav14p2739s1.zip › supplementary/raw data/Figure 1/MNX1-heatmap.tif]

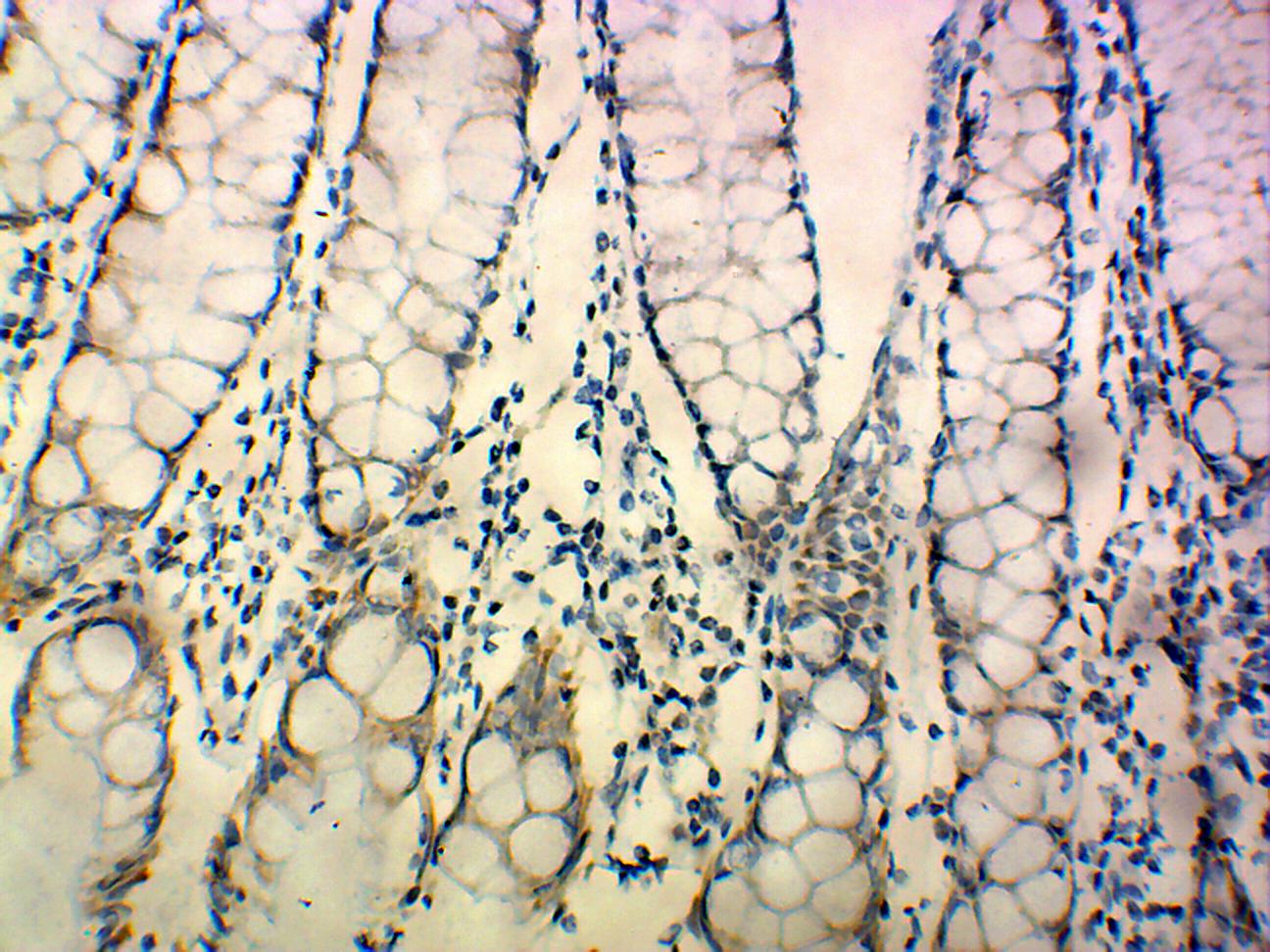

Supplement: Supplementary file 1 — Supplementary figures and tables. [file jcav14p2739s1.zip › supplementary/raw data/Figure 1/pa-4(1).JPG]

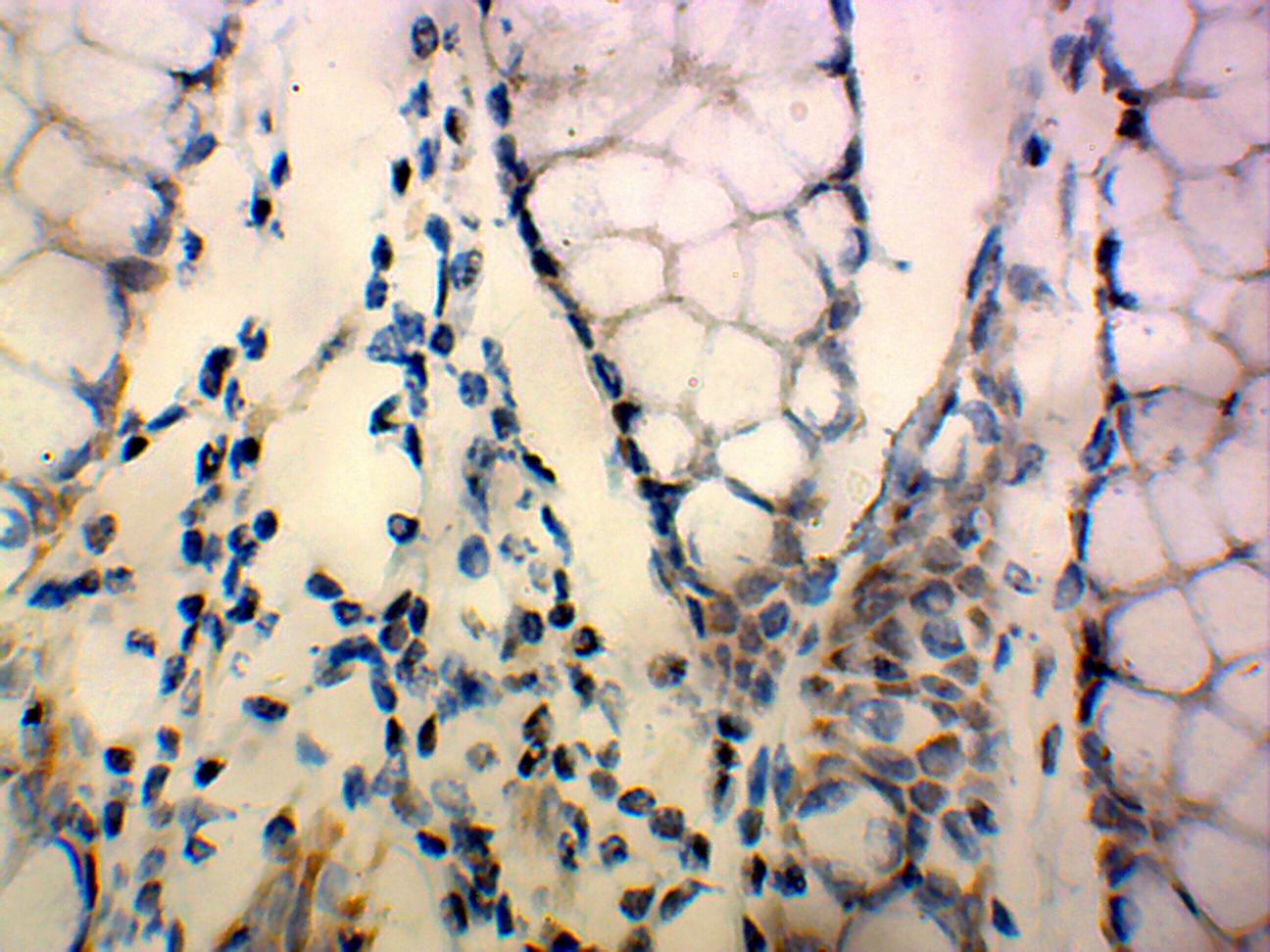

Supplement: Supplementary file 1 — Supplementary figures and tables. [file jcav14p2739s1.zip › supplementary/raw data/Figure 1/pa-4(2).JPG]

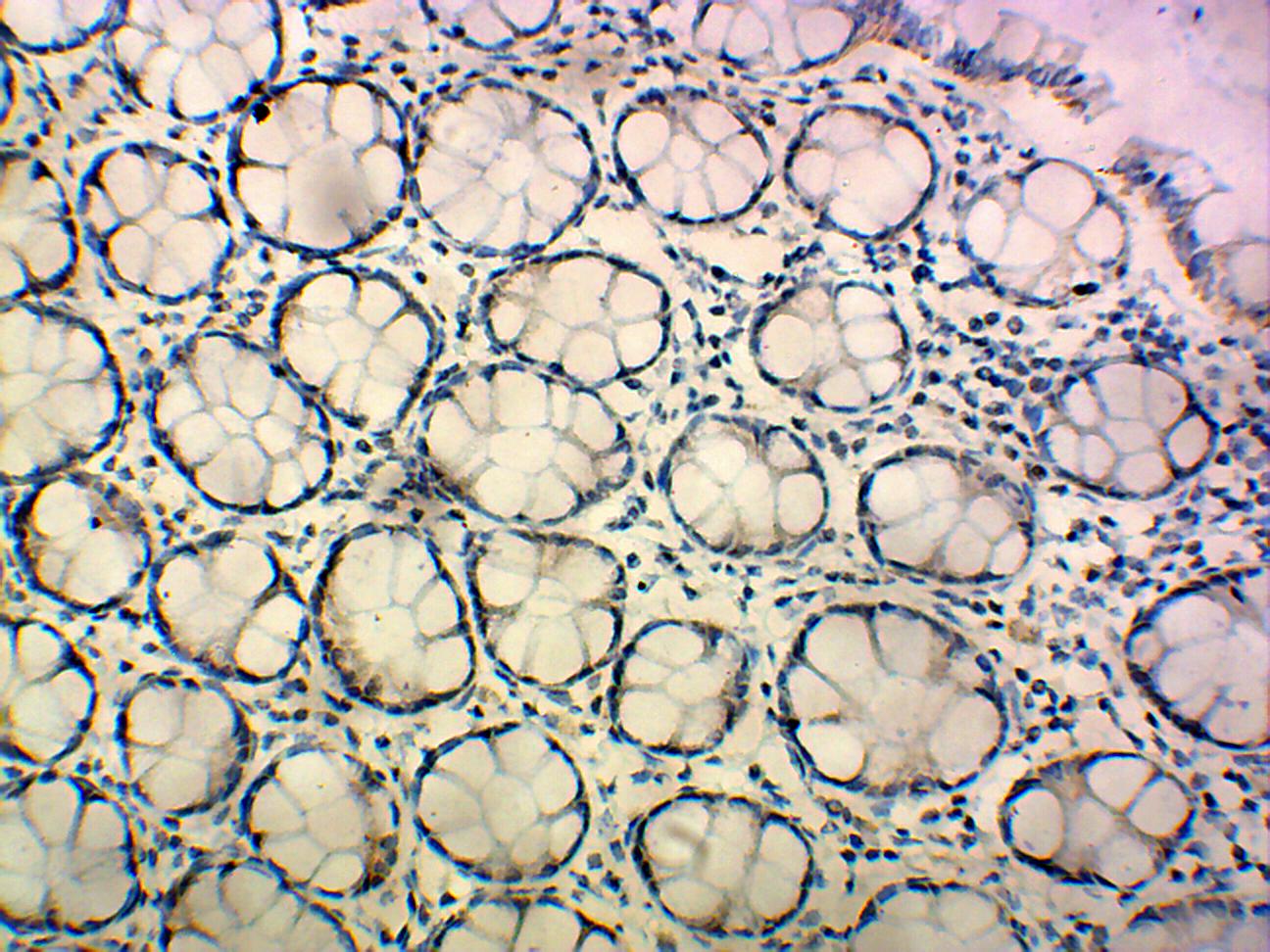

Supplement: Supplementary file 1 — Supplementary figures and tables. [file jcav14p2739s1.zip › supplementary/raw data/Figure 1/pa-5(1).JPG]

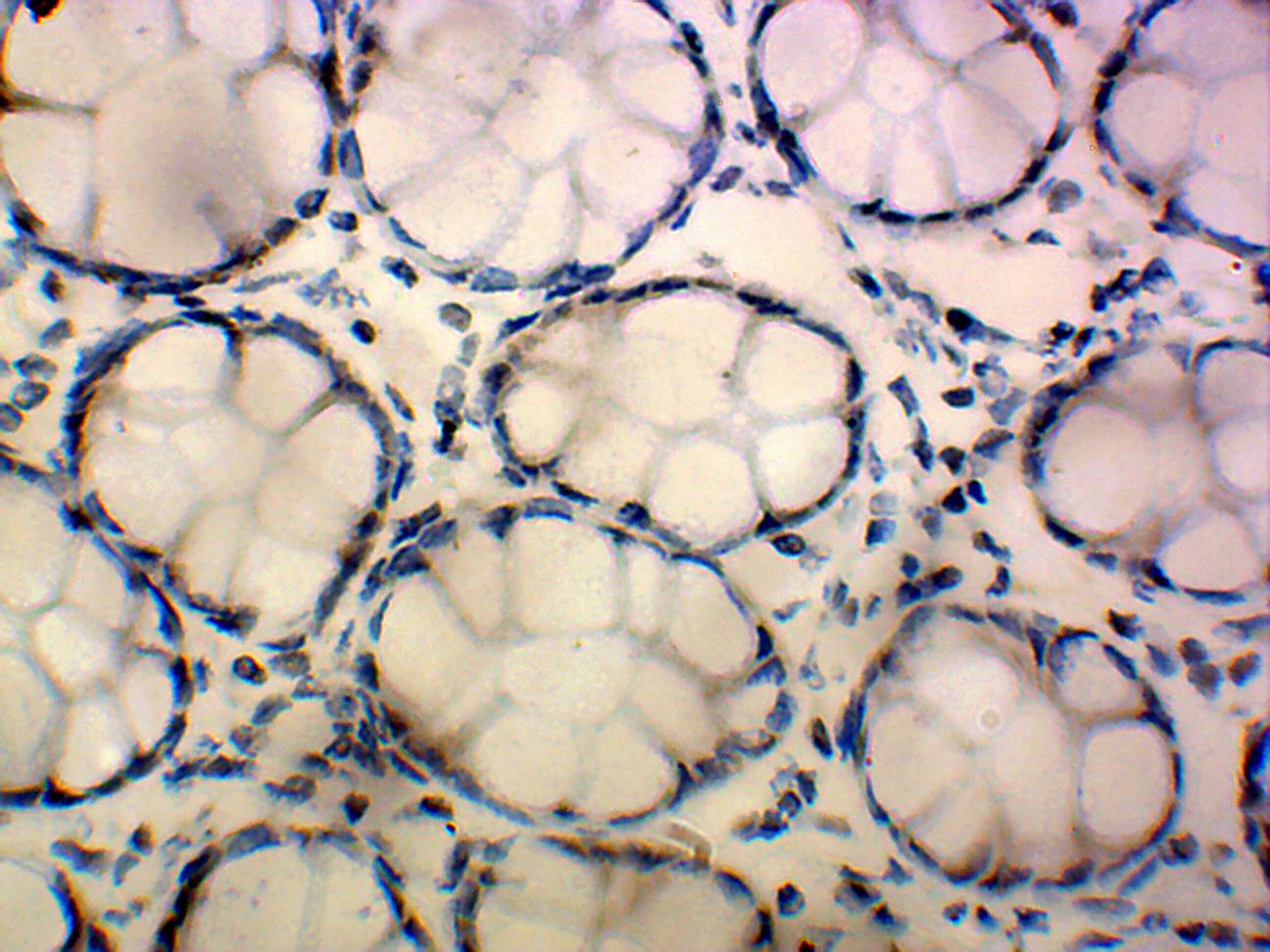

Supplement: Supplementary file 1 — Supplementary figures and tables. [file jcav14p2739s1.zip › supplementary/raw data/Figure 1/pa-5(2).JPG]

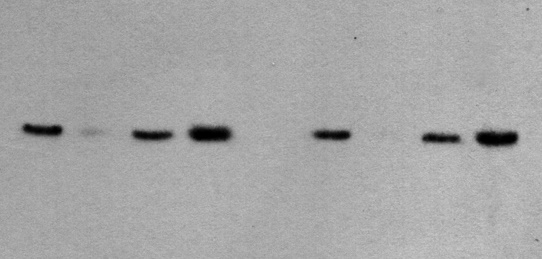

Supplement: Supplementary file 1 — Supplementary figures and tables. [file jcav14p2739s1.zip › supplementary/raw data/Figure 2/f2-1.tif]

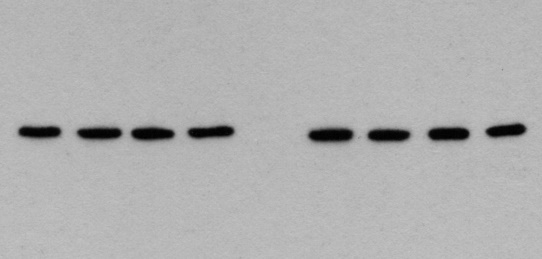

Supplement: Supplementary file 1 — Supplementary figures and tables. [file jcav14p2739s1.zip › supplementary/raw data/Figure 2/f2-1b.tif]

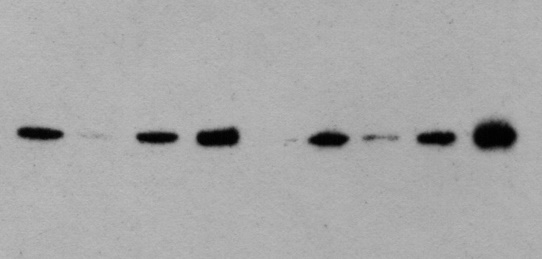

Supplement: Supplementary file 1 — Supplementary figures and tables. [file jcav14p2739s1.zip › supplementary/raw data/Figure 2/f2-2.tif]

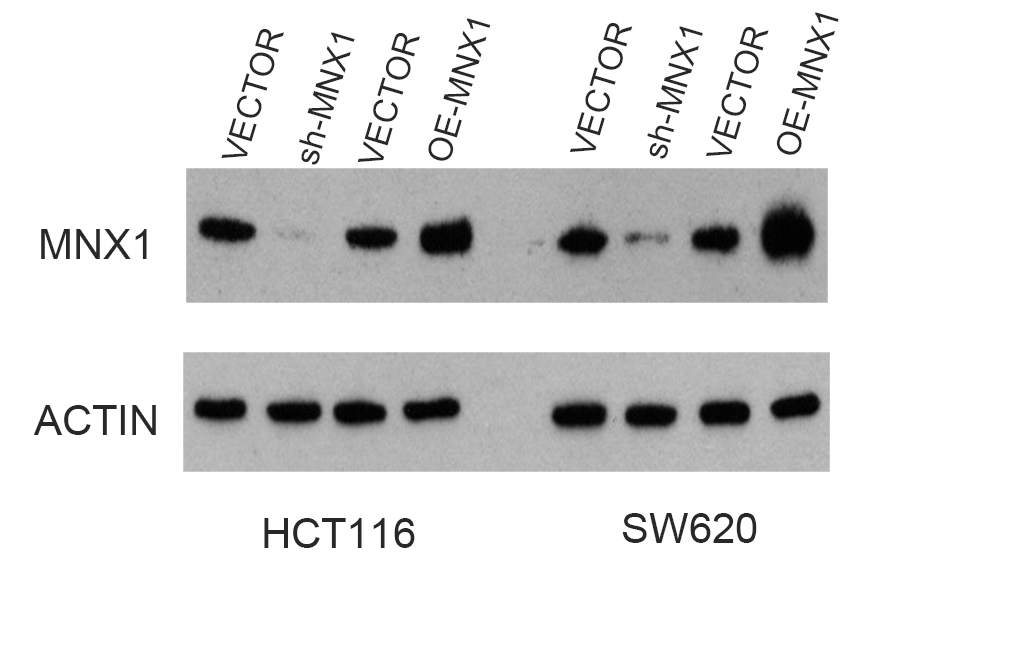

Supplement: Supplementary file 1 — Supplementary figures and tables. [file jcav14p2739s1.zip › supplementary/raw data/Figure 2/F2B-F3Brepeat.tif]

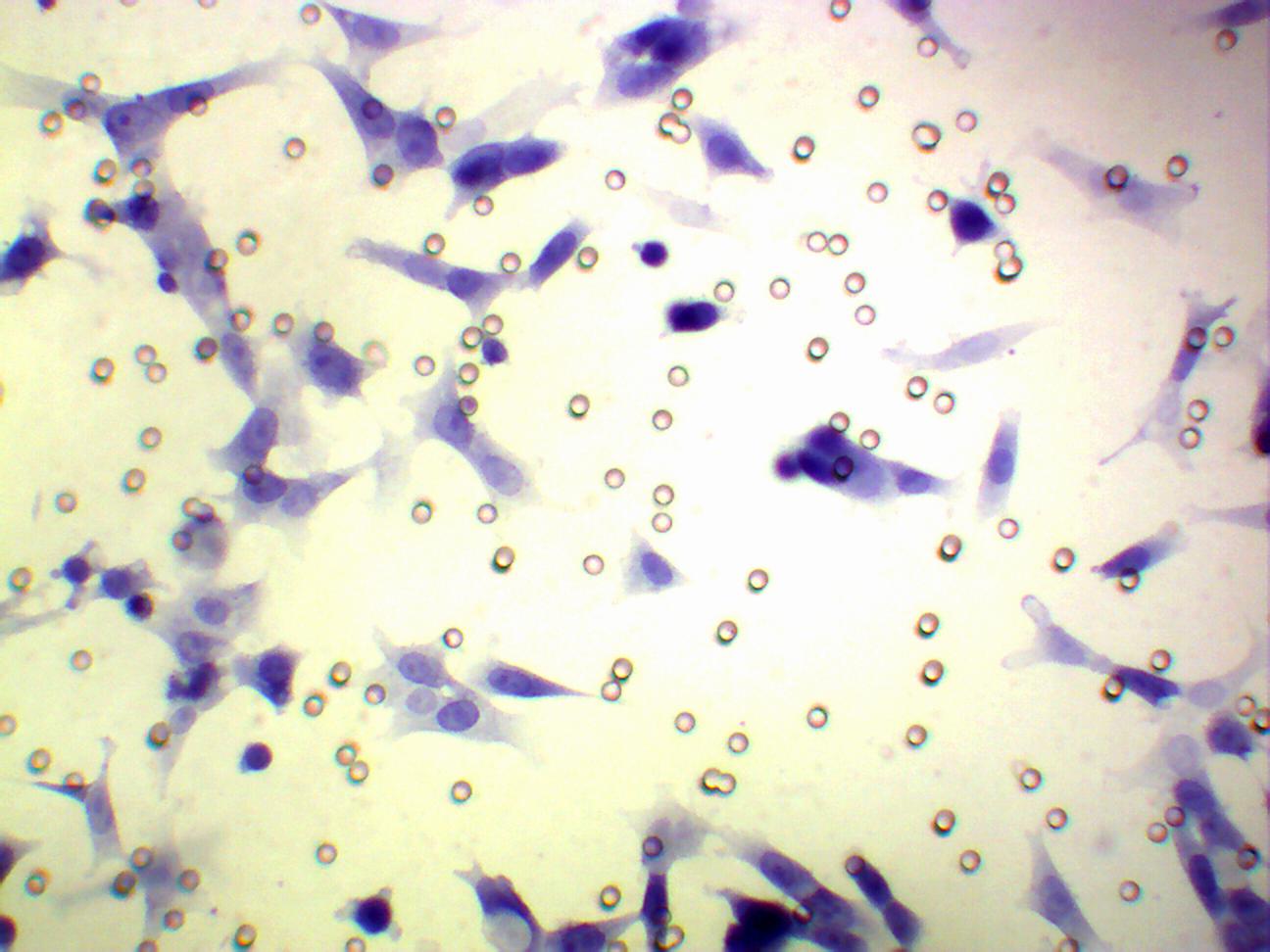

Supplement: Supplementary file 1 — Supplementary figures and tables. [file jcav14p2739s1.zip › supplementary/raw data/Figure 2/HCT116/MNX1 1.JPG]

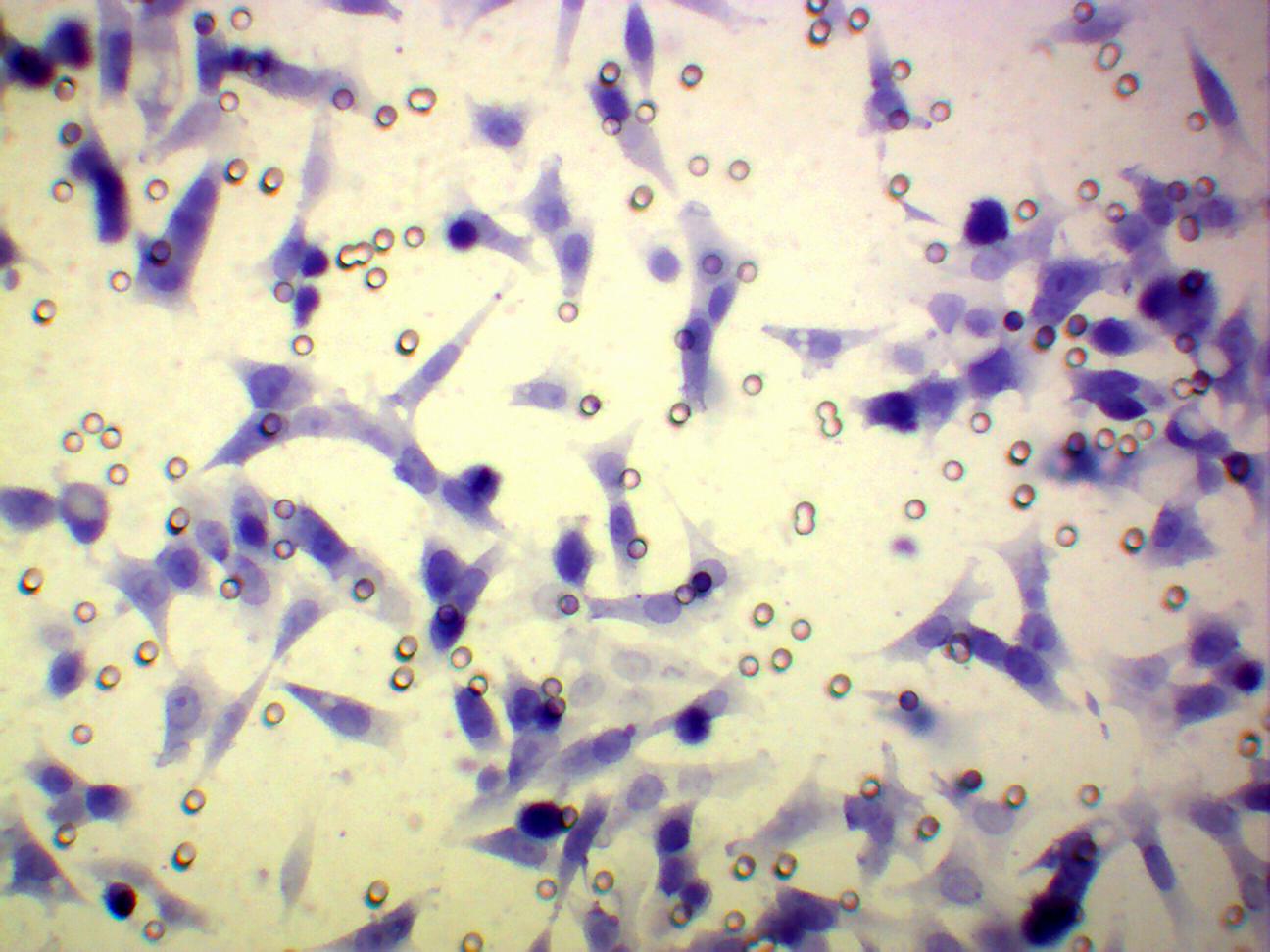

Supplement: Supplementary file 1 — Supplementary figures and tables. [file jcav14p2739s1.zip › supplementary/raw data/Figure 2/HCT116/MNX1 3.JPG]

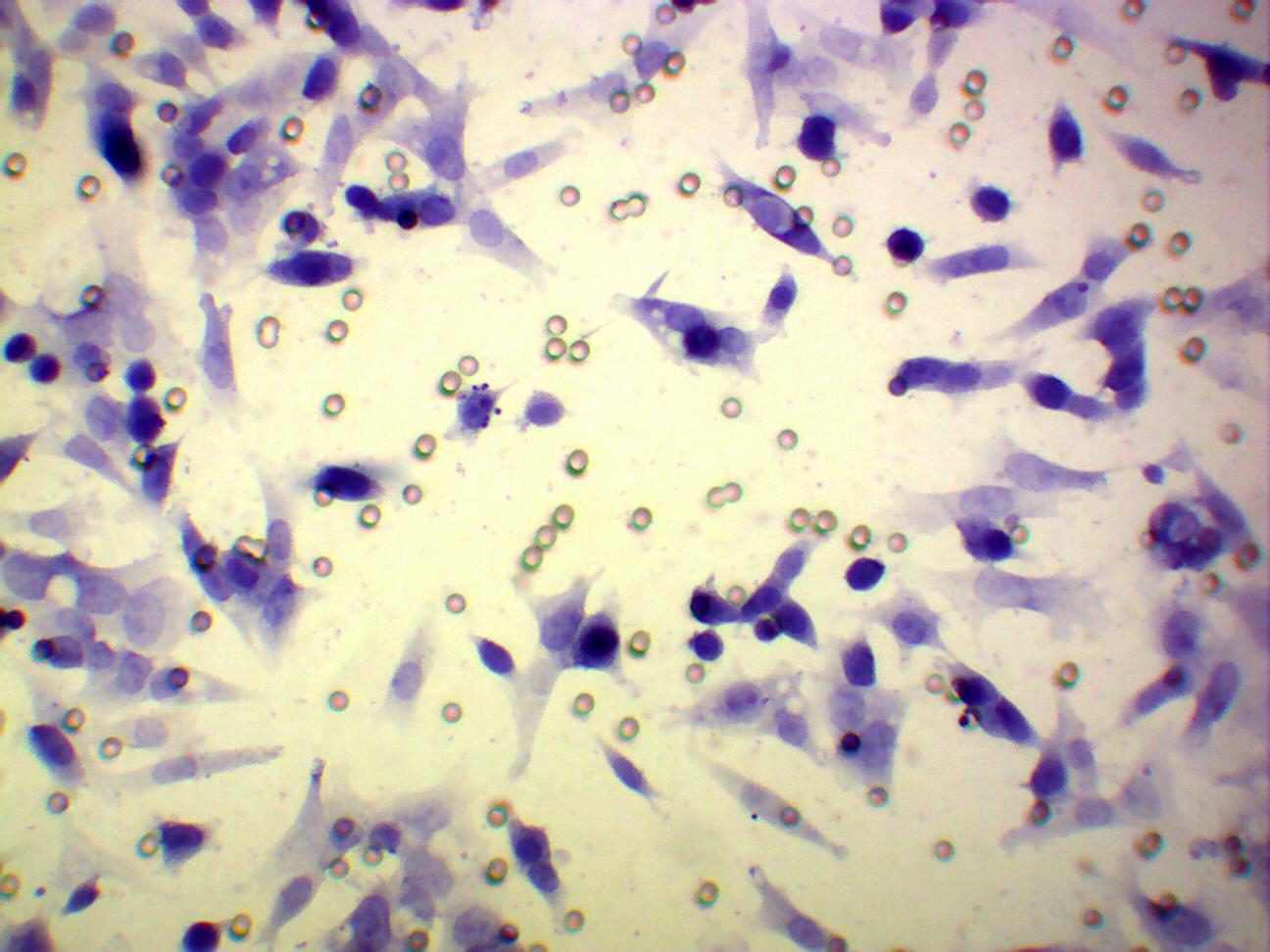

Supplement: Supplementary file 1 — Supplementary figures and tables. [file jcav14p2739s1.zip › supplementary/raw data/Figure 2/HCT116/MNX14.JPG]

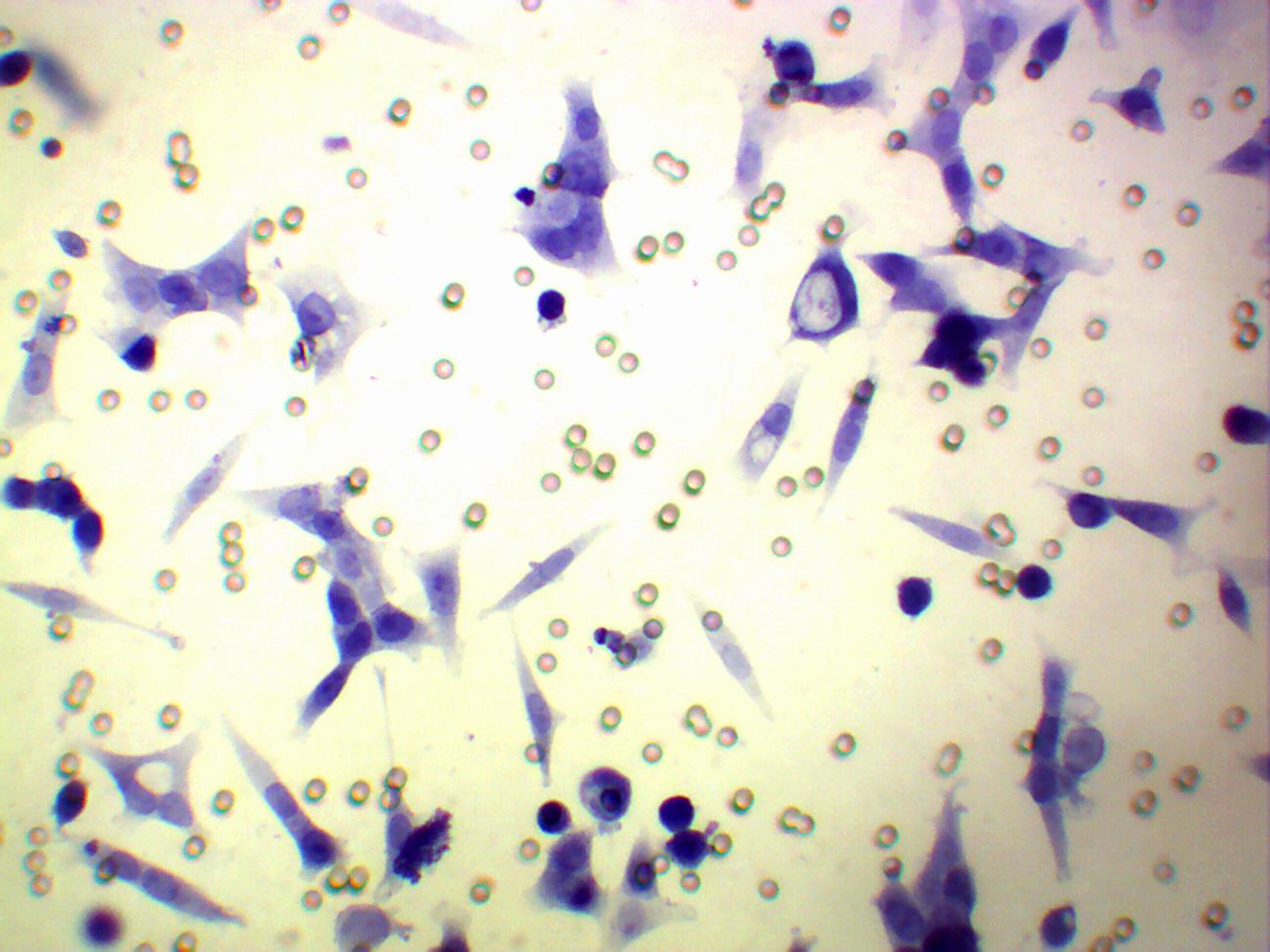

Supplement: Supplementary file 1 — Supplementary figures and tables. [file jcav14p2739s1.zip › supplementary/raw data/Figure 2/HCT116/MNX1(2).JPG]

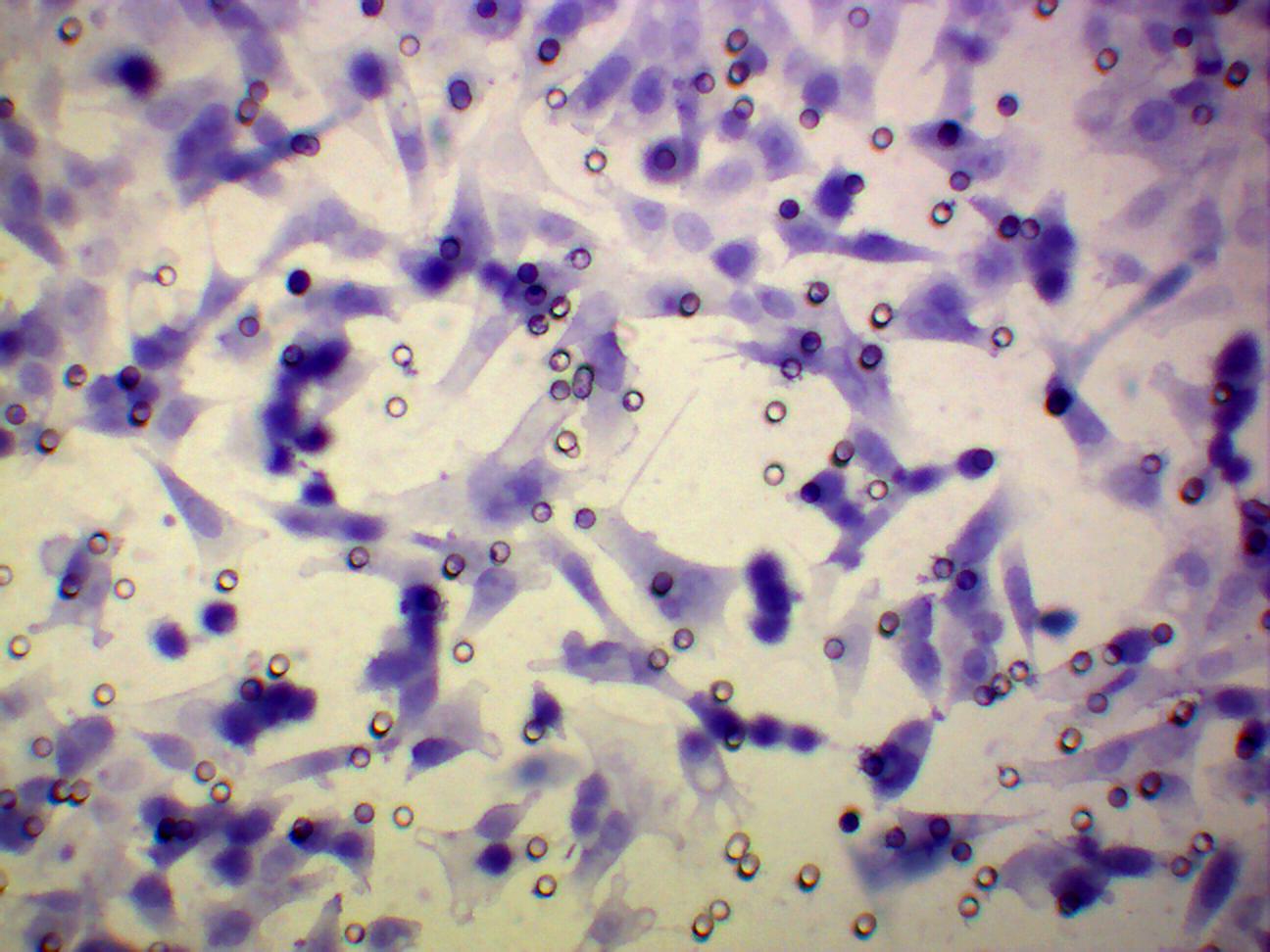

Supplement: Supplementary file 1 — Supplementary figures and tables. [file jcav14p2739s1.zip › supplementary/raw data/Figure 2/HCT116/NC 3.JPG]

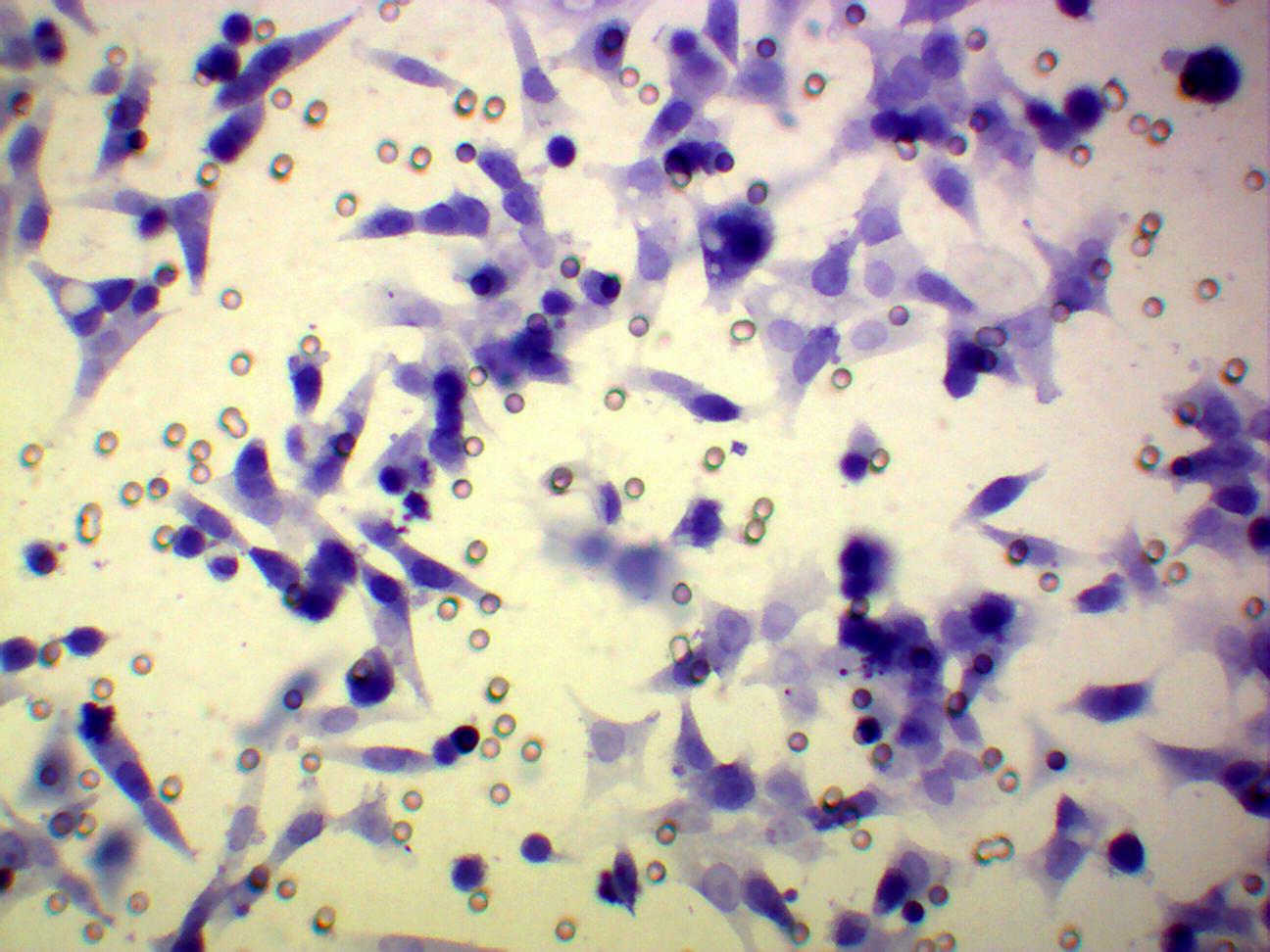

Supplement: Supplementary file 1 — Supplementary figures and tables. [file jcav14p2739s1.zip › supplementary/raw data/Figure 2/HCT116/NC(1).JPG]

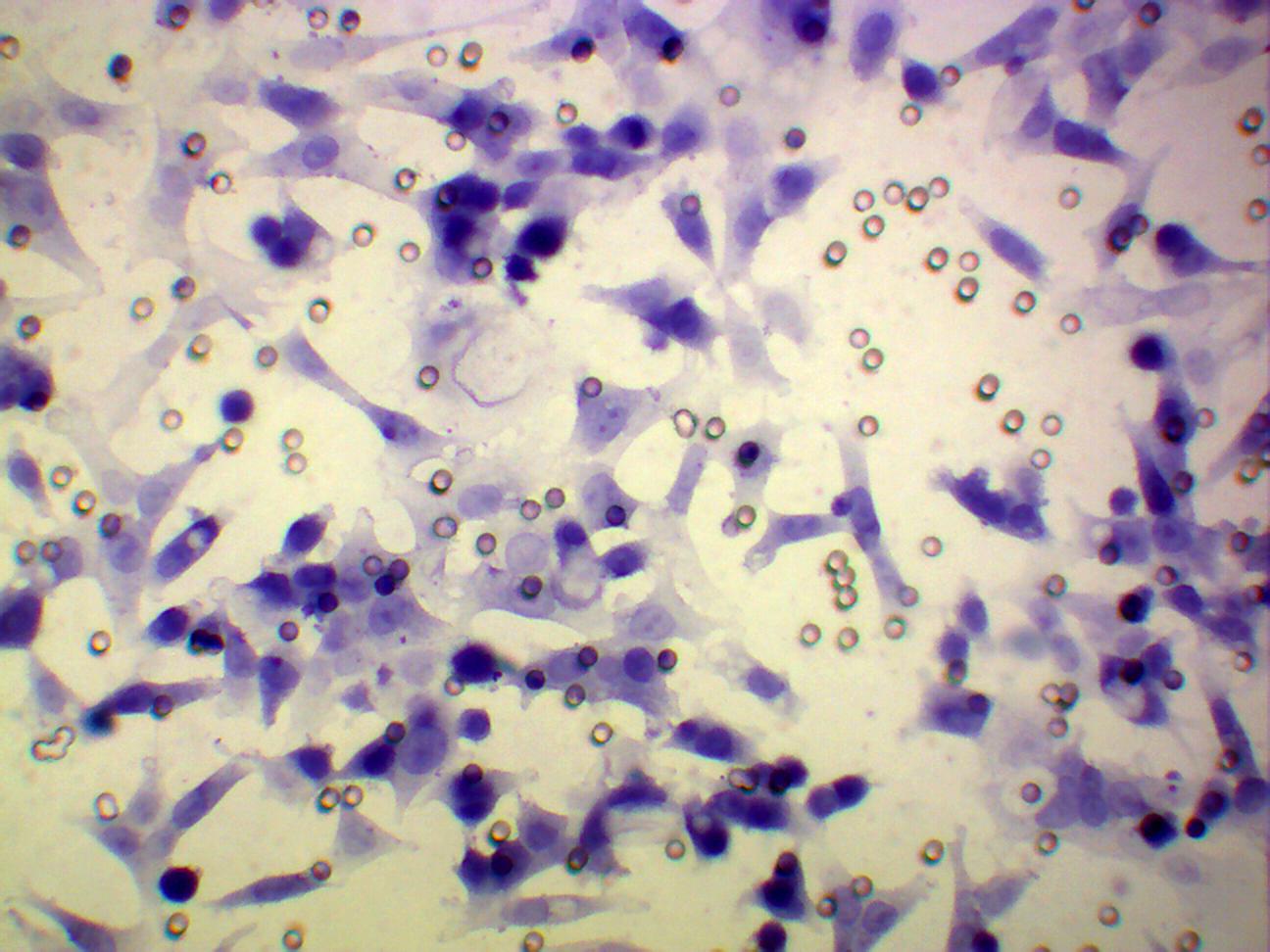

Supplement: Supplementary file 1 — Supplementary figures and tables. [file jcav14p2739s1.zip › supplementary/raw data/Figure 2/HCT116/NC(2).JPG]

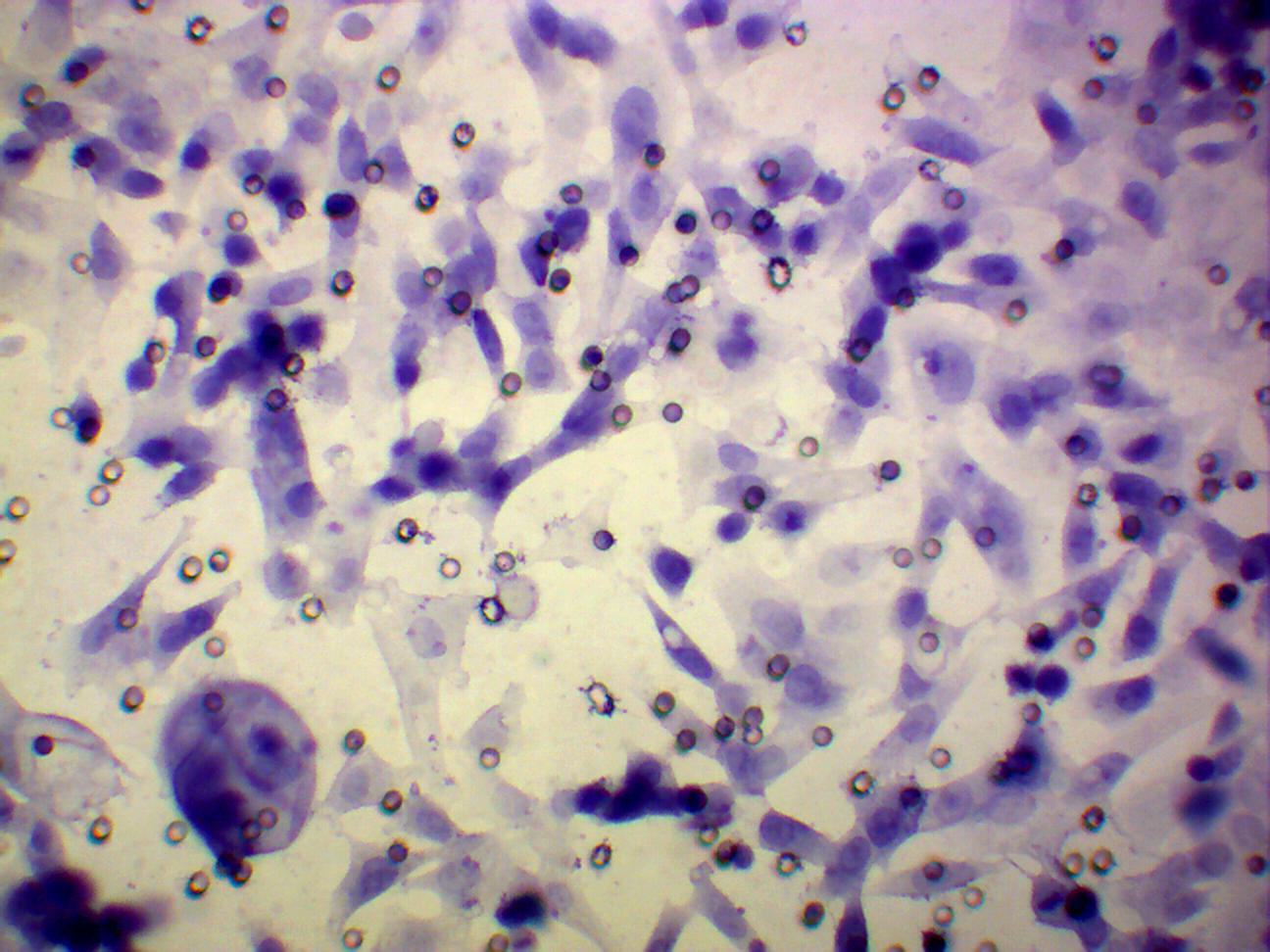

Supplement: Supplementary file 1 — Supplementary figures and tables. [file jcav14p2739s1.zip › supplementary/raw data/Figure 2/HCT116/NC(4).JPG]

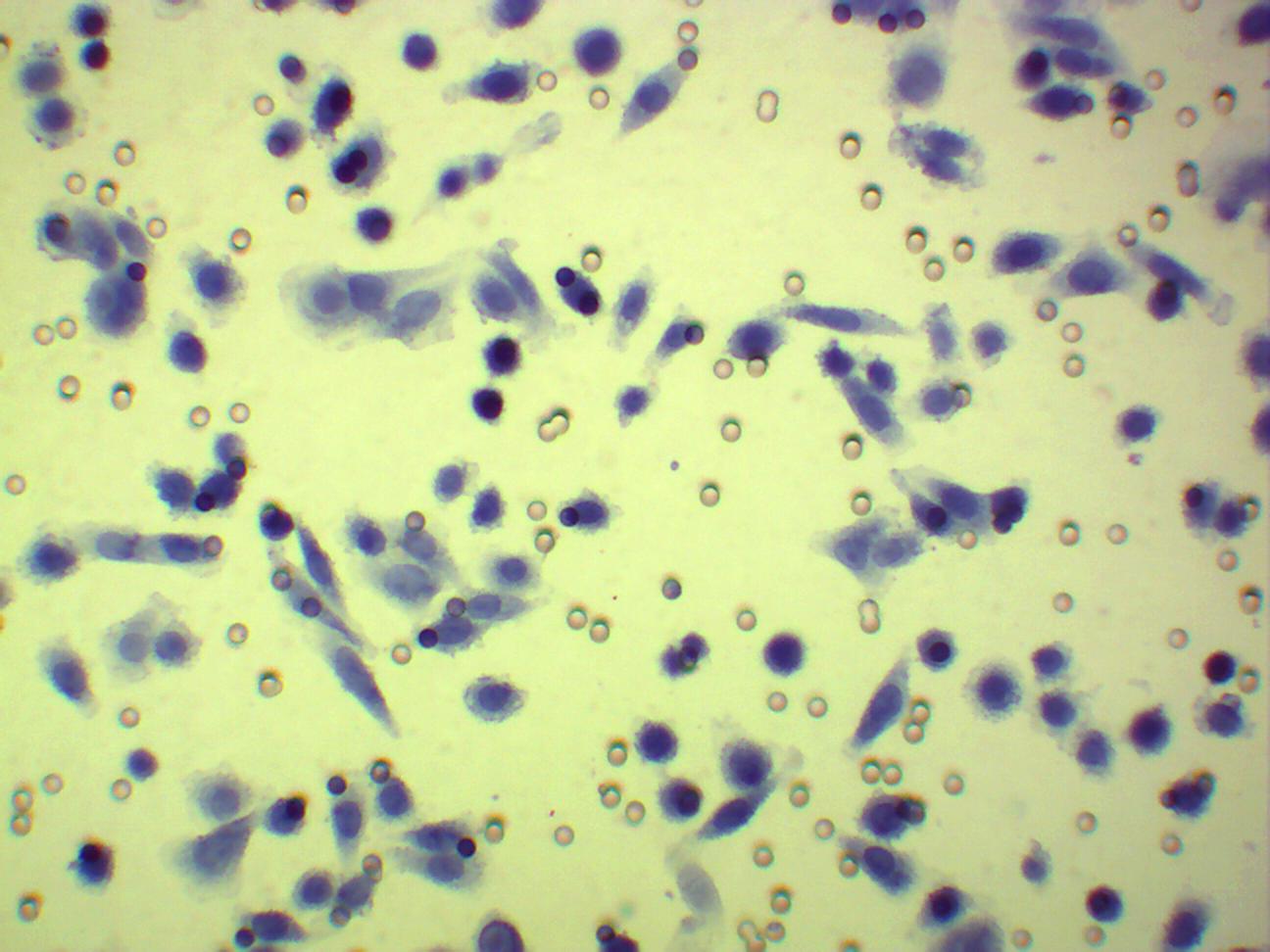

Supplement: Supplementary file 1 — Supplementary figures and tables. [file jcav14p2739s1.zip › supplementary/raw data/Figure 2/SW620/MNX1(1) .JPG]

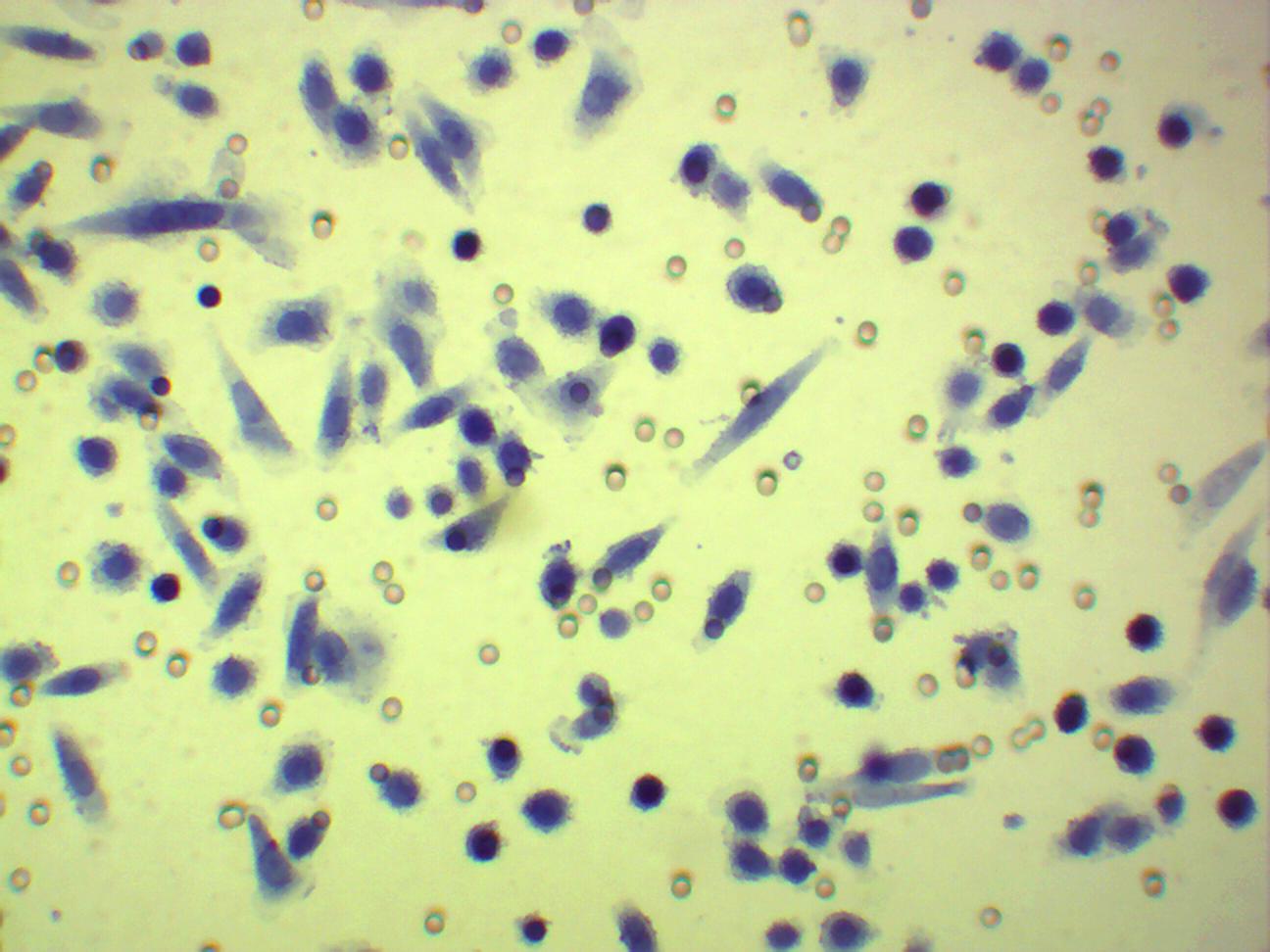

Supplement: Supplementary file 1 — Supplementary figures and tables. [file jcav14p2739s1.zip › supplementary/raw data/Figure 2/SW620/MNX1(2).JPG]

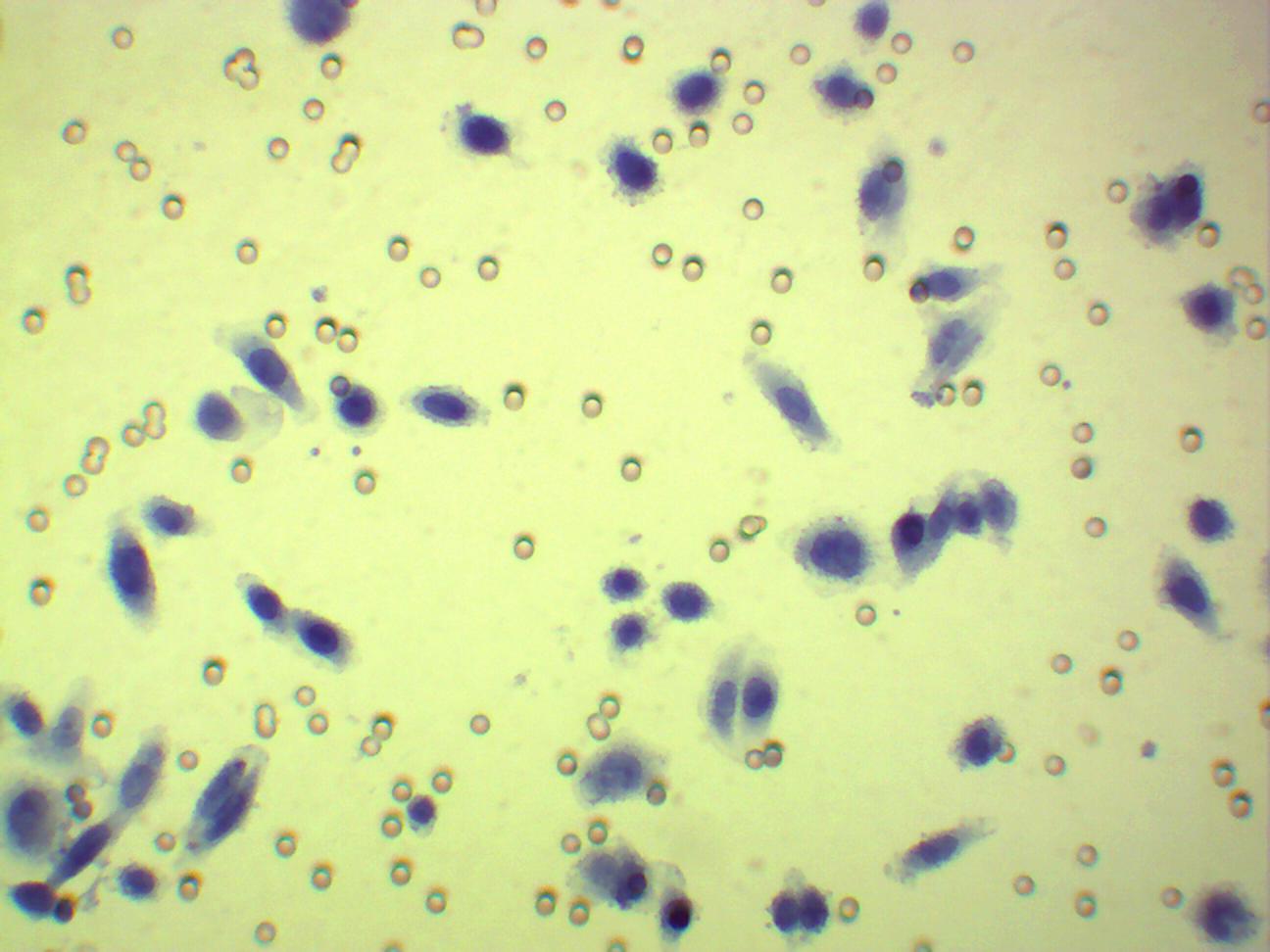

Supplement: Supplementary file 1 — Supplementary figures and tables. [file jcav14p2739s1.zip › supplementary/raw data/Figure 2/SW620/MNX1(3).JPG]

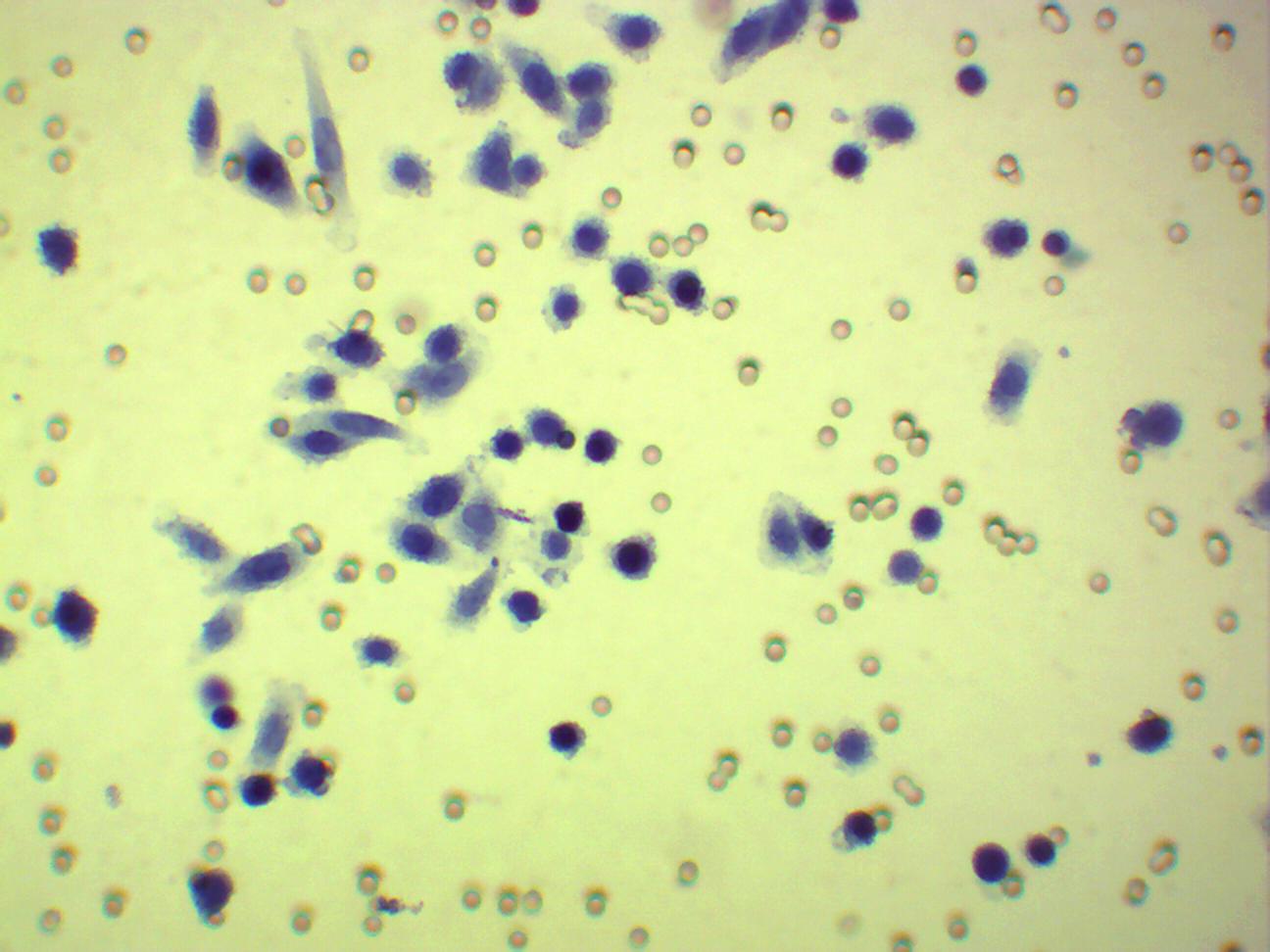

Supplement: Supplementary file 1 — Supplementary figures and tables. [file jcav14p2739s1.zip › supplementary/raw data/Figure 2/SW620/MNX1(4).JPG]

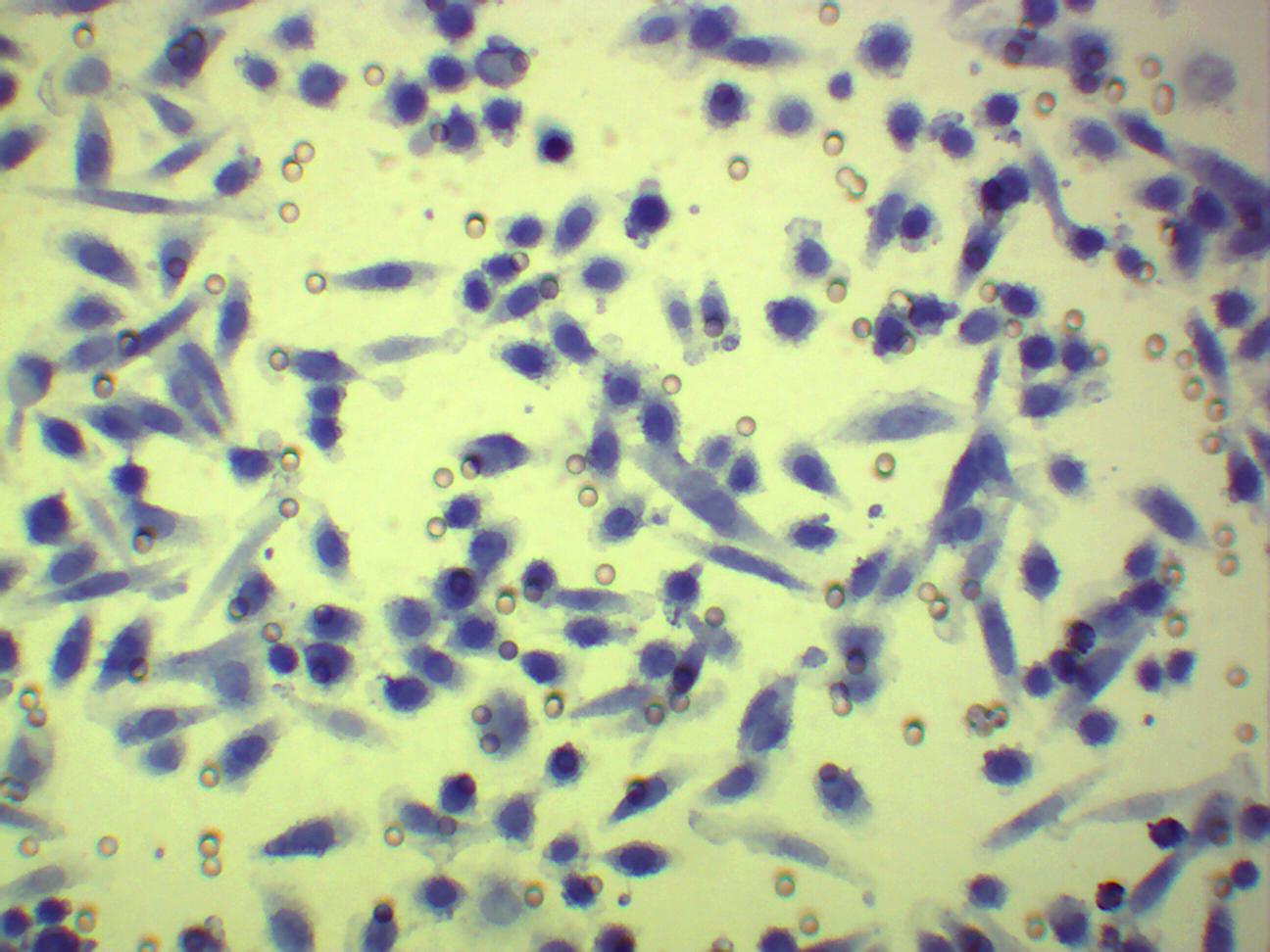

Supplement: Supplementary file 1 — Supplementary figures and tables. [file jcav14p2739s1.zip › supplementary/raw data/Figure 2/SW620/NC (1).JPG]

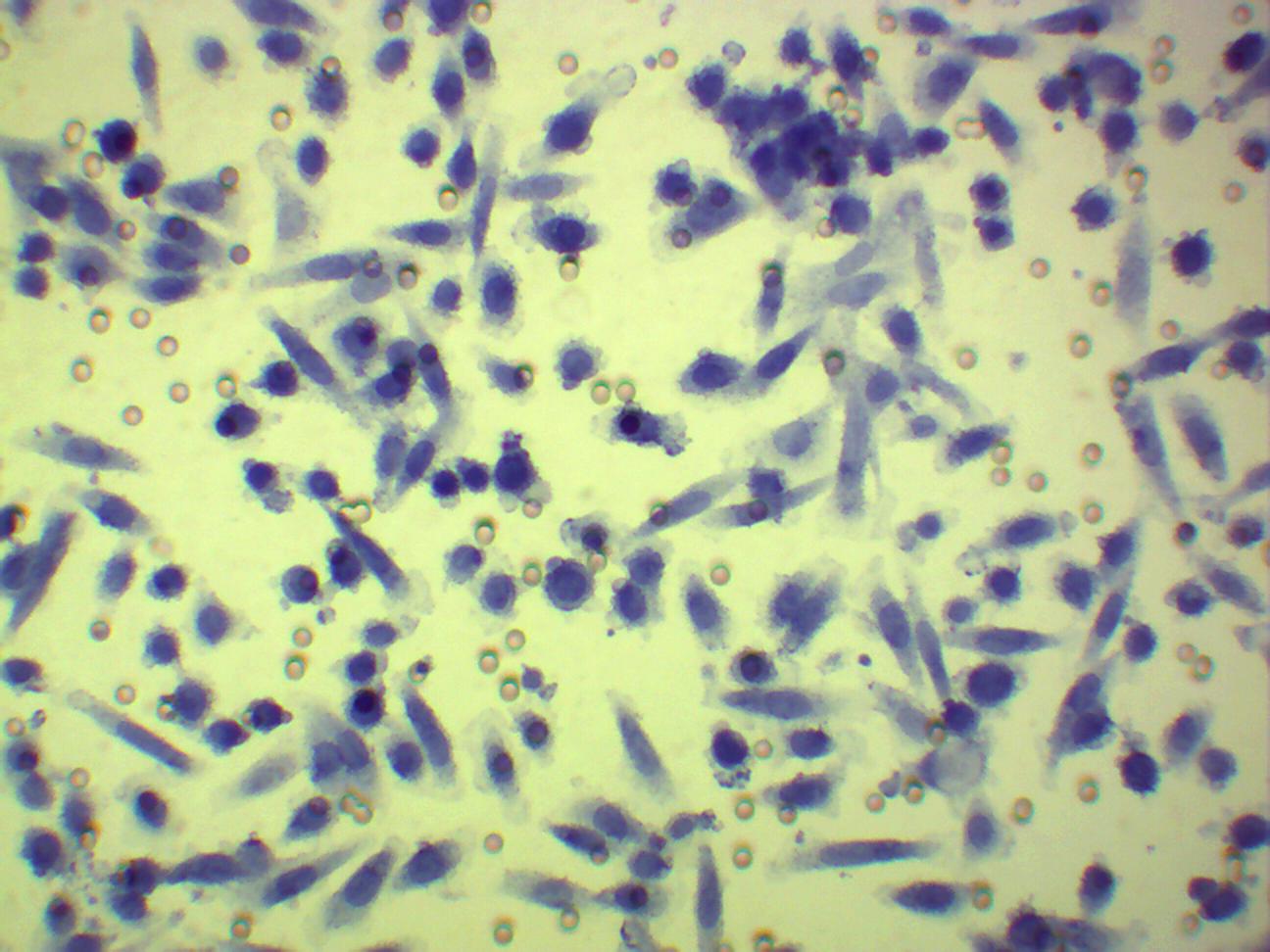

Supplement: Supplementary file 1 — Supplementary figures and tables. [file jcav14p2739s1.zip › supplementary/raw data/Figure 2/SW620/NC (2).JPG]

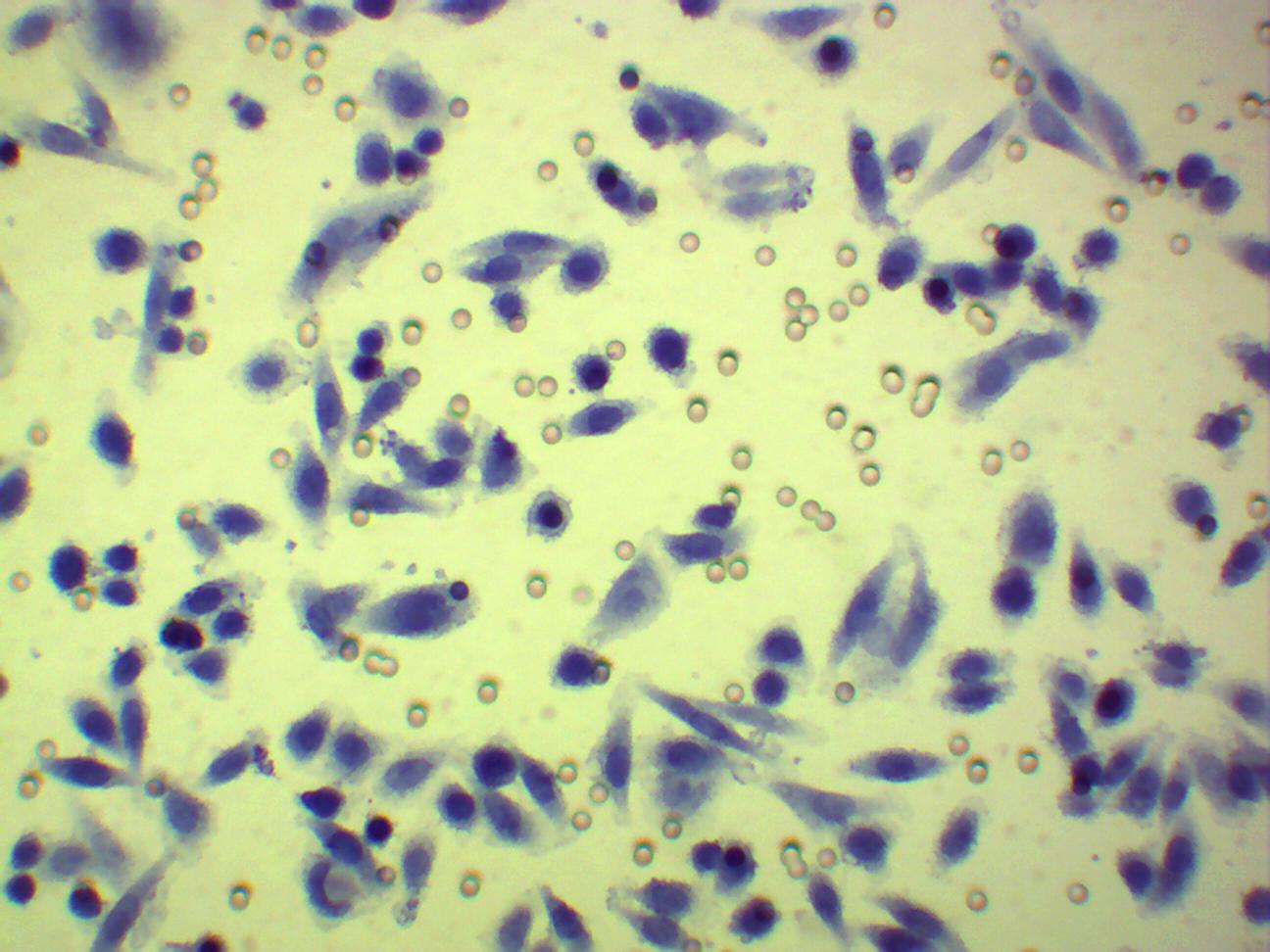

Supplement: Supplementary file 1 — Supplementary figures and tables. [file jcav14p2739s1.zip › supplementary/raw data/Figure 2/SW620/NC(3).JPG]

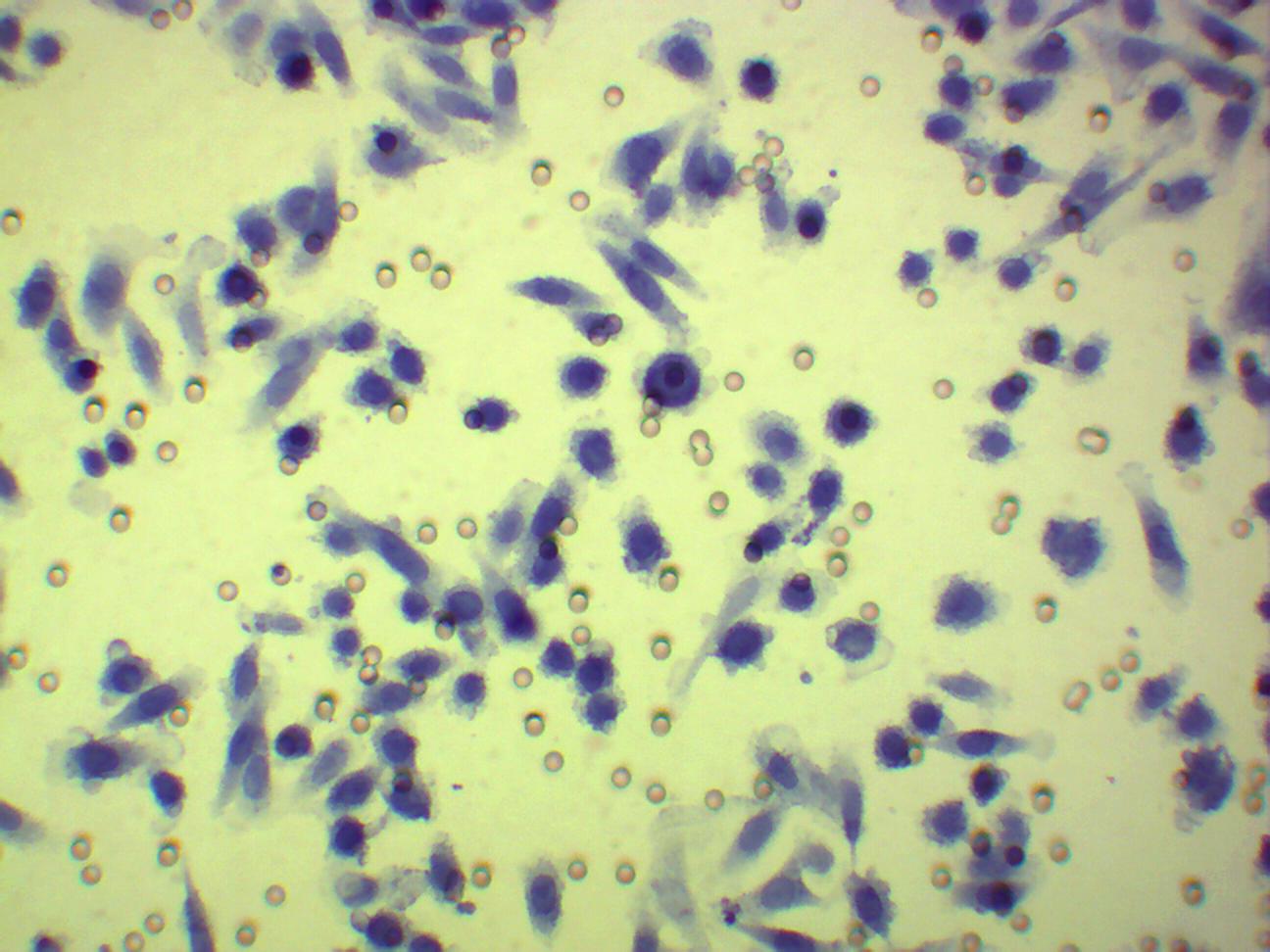

Supplement: Supplementary file 1 — Supplementary figures and tables. [file jcav14p2739s1.zip › supplementary/raw data/Figure 2/SW620/NC(4).JPG]

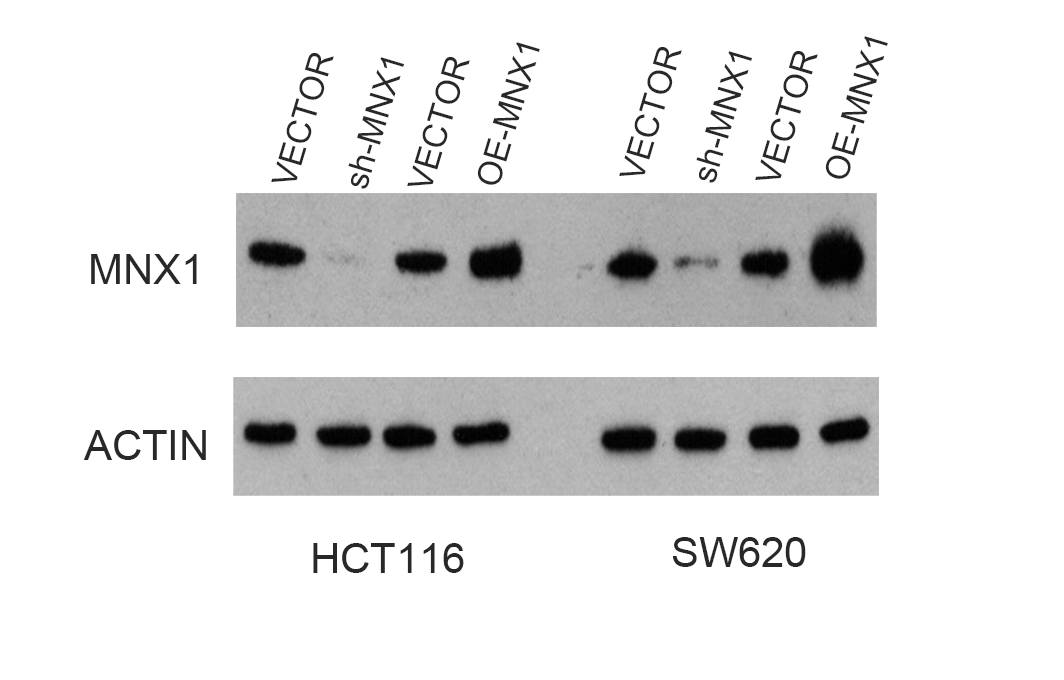

Supplement: Supplementary file 1 — Supplementary figures and tables. [file jcav14p2739s1.zip › supplementary/raw data/Figure 3/F2B-F3B repeat.tif]

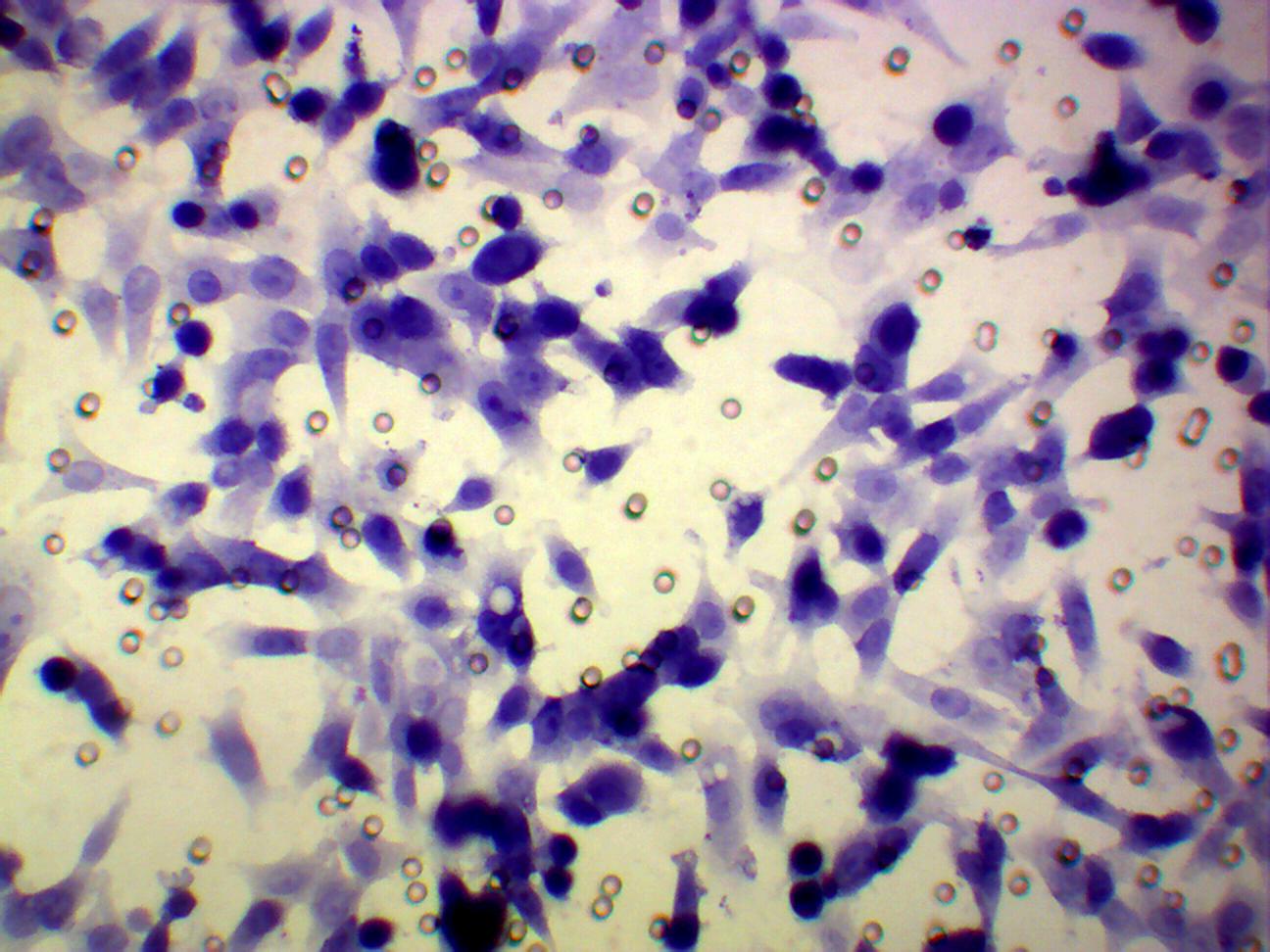

Supplement: Supplementary file 1 — Supplementary figures and tables. [file jcav14p2739s1.zip › supplementary/raw data/Figure 3/HCT116/MNX1 (1).JPG]

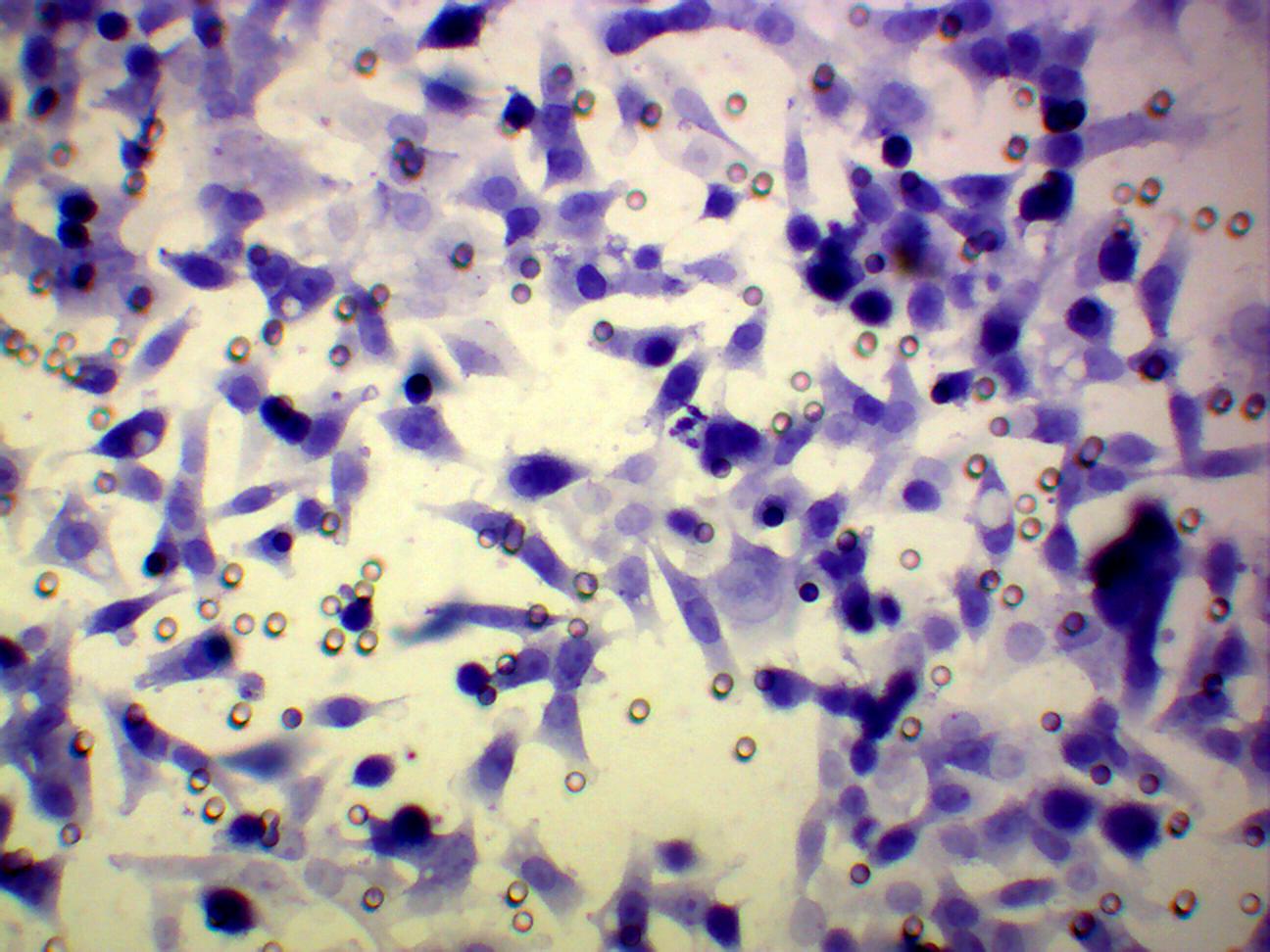

Supplement: Supplementary file 1 — Supplementary figures and tables. [file jcav14p2739s1.zip › supplementary/raw data/Figure 3/HCT116/MNX1 (2).JPG]

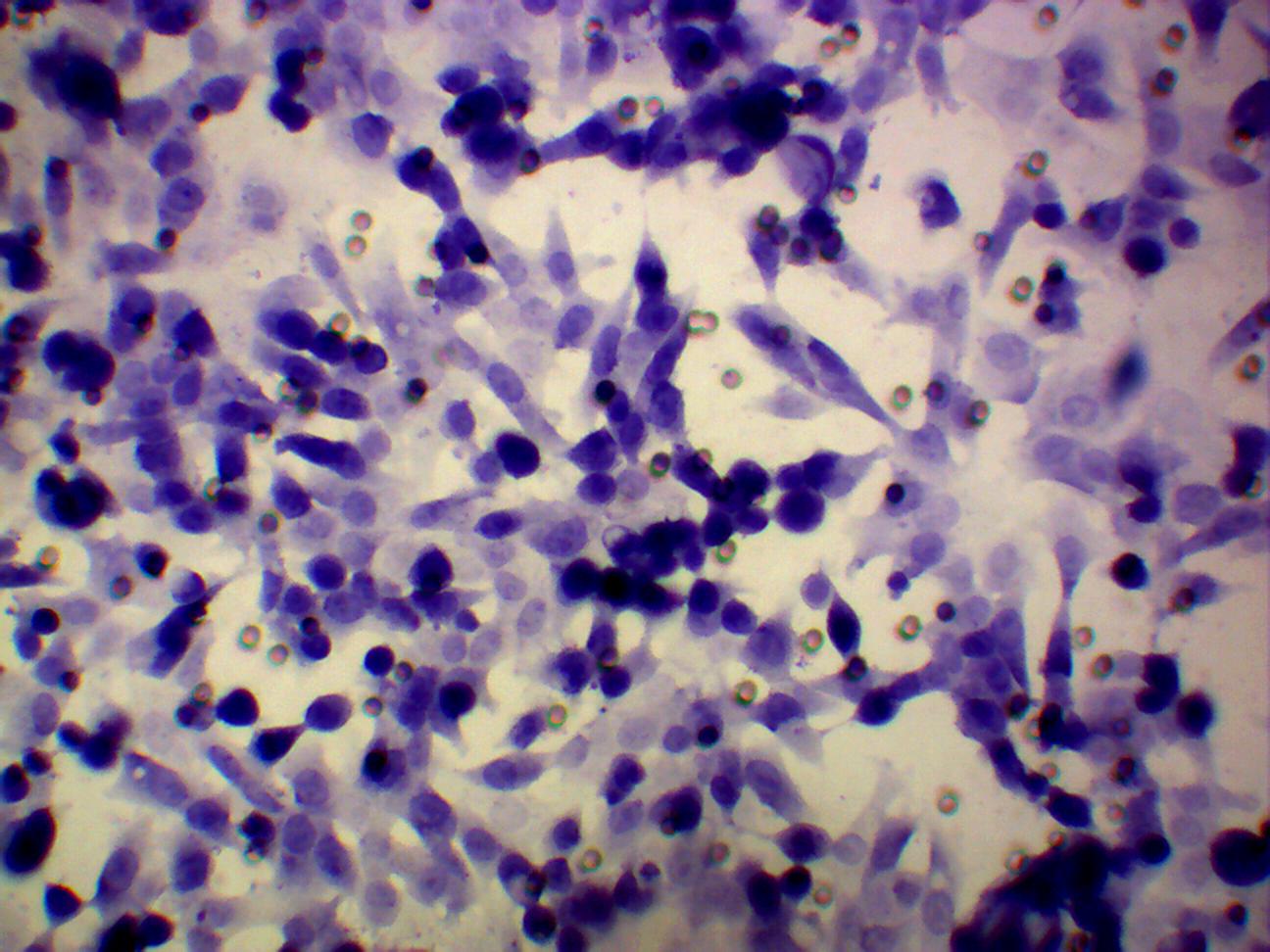

Supplement: Supplementary file 1 — Supplementary figures and tables. [file jcav14p2739s1.zip › supplementary/raw data/Figure 3/HCT116/MNX1(1).JPG]

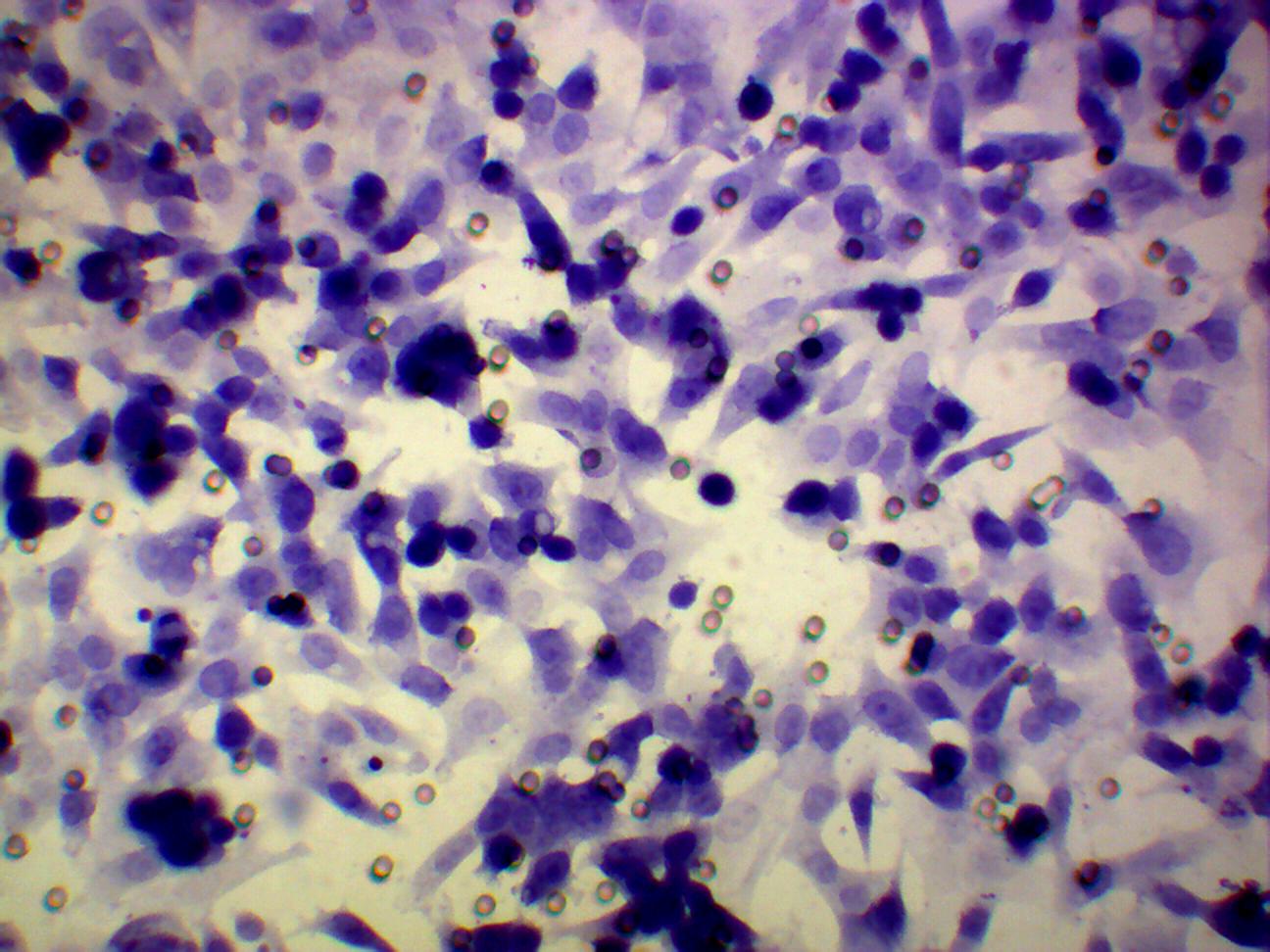

Supplement: Supplementary file 1 — Supplementary figures and tables. [file jcav14p2739s1.zip › supplementary/raw data/Figure 3/HCT116/MNX1(2).JPG]

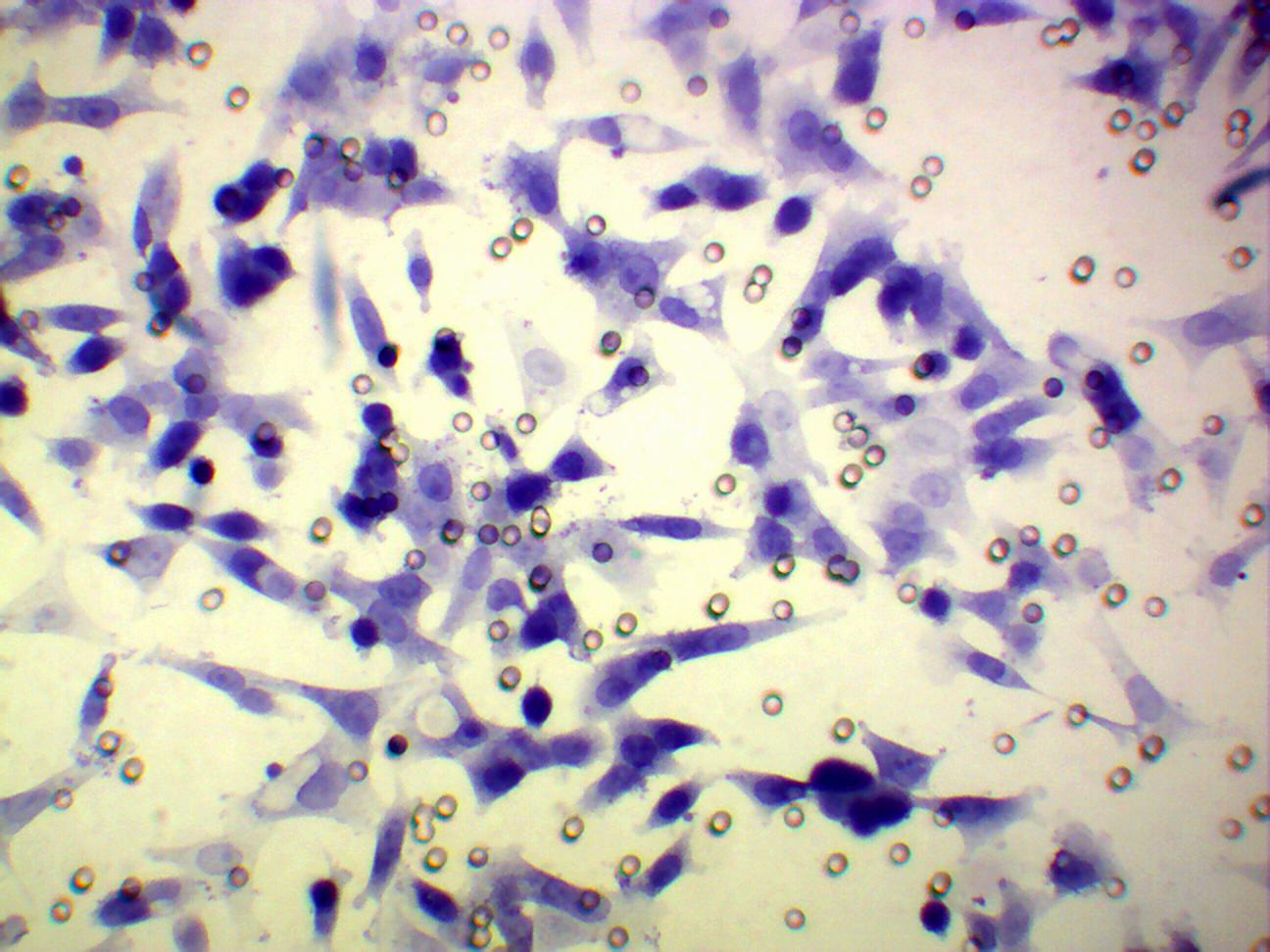

Supplement: Supplementary file 1 — Supplementary figures and tables. [file jcav14p2739s1.zip › supplementary/raw data/Figure 3/HCT116/Vector (1).JPG]

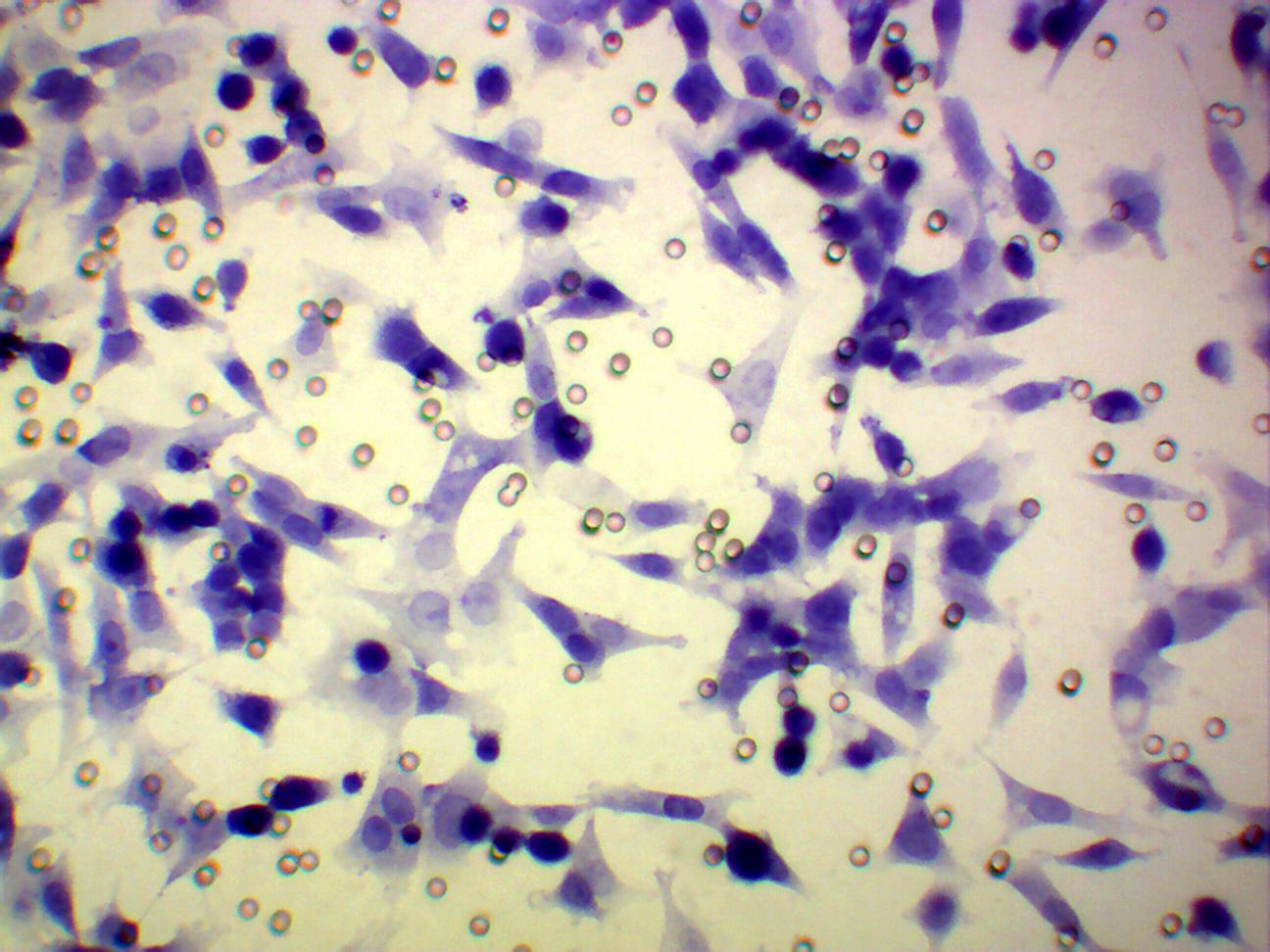

Supplement: Supplementary file 1 — Supplementary figures and tables. [file jcav14p2739s1.zip › supplementary/raw data/Figure 3/HCT116/Vector (2).JPG]

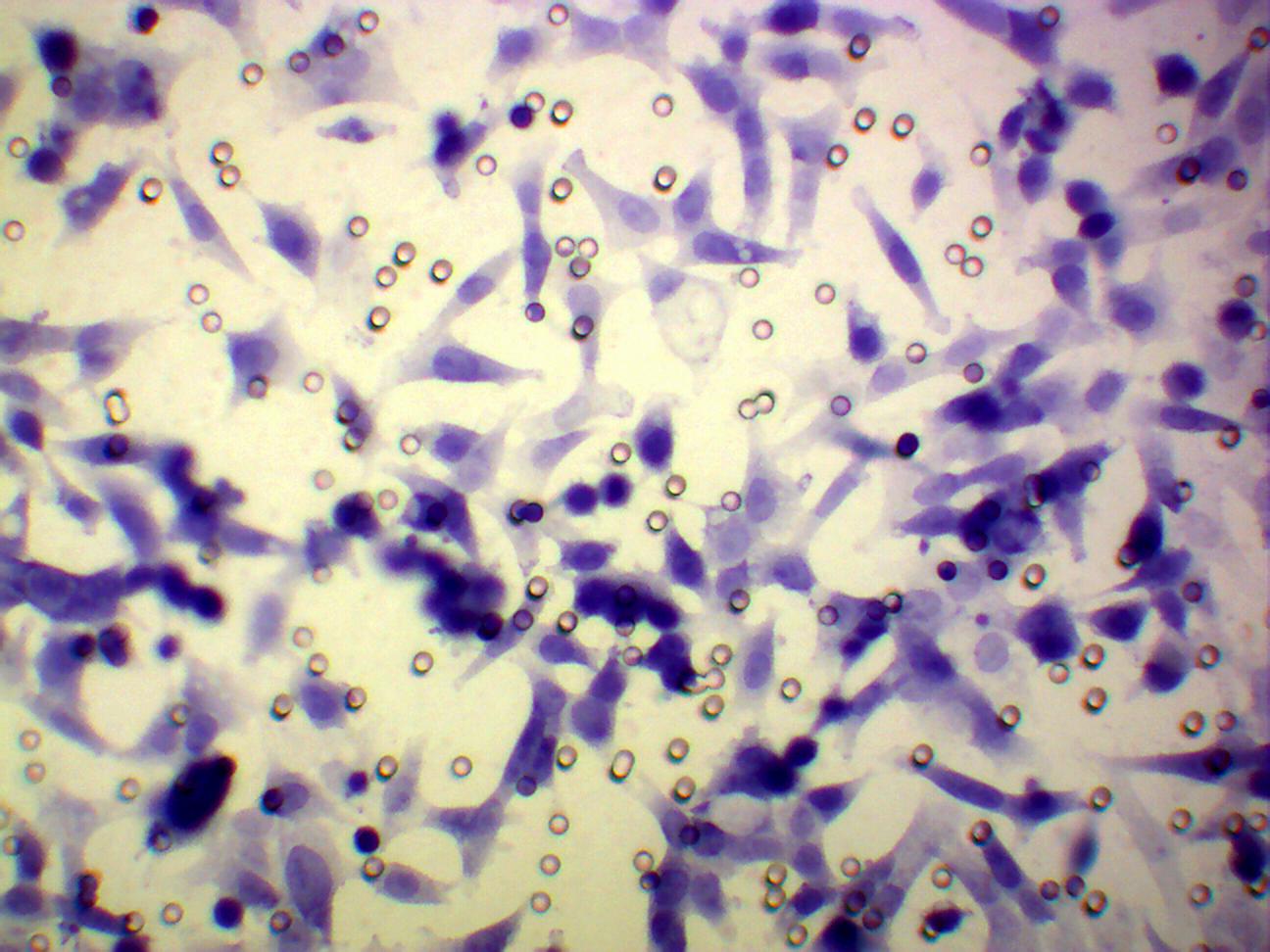

Supplement: Supplementary file 1 — Supplementary figures and tables. [file jcav14p2739s1.zip › supplementary/raw data/Figure 3/HCT116/Vector(1).JPG]

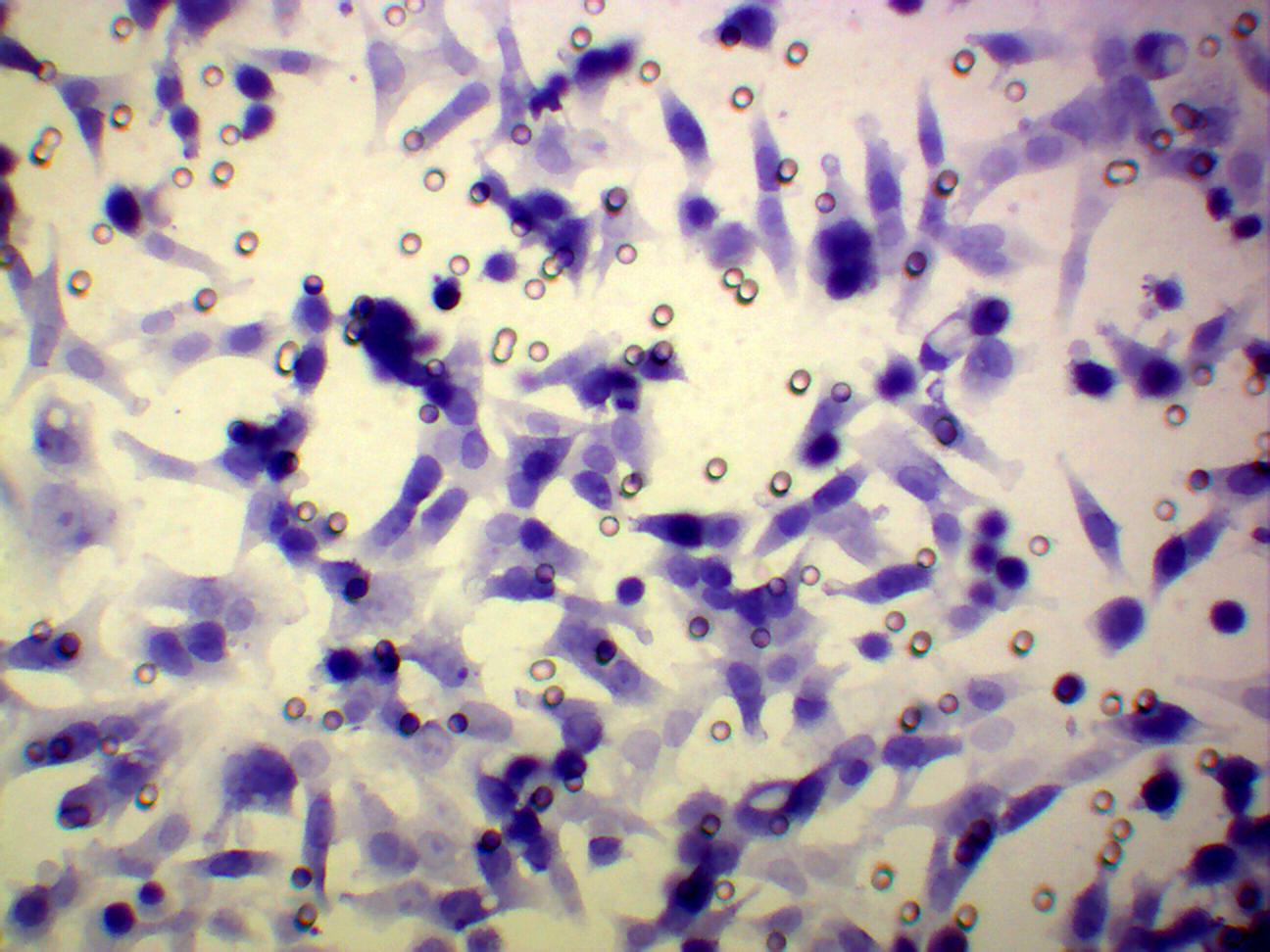

Supplement: Supplementary file 1 — Supplementary figures and tables. [file jcav14p2739s1.zip › supplementary/raw data/Figure 3/HCT116/Vector(2).JPG]

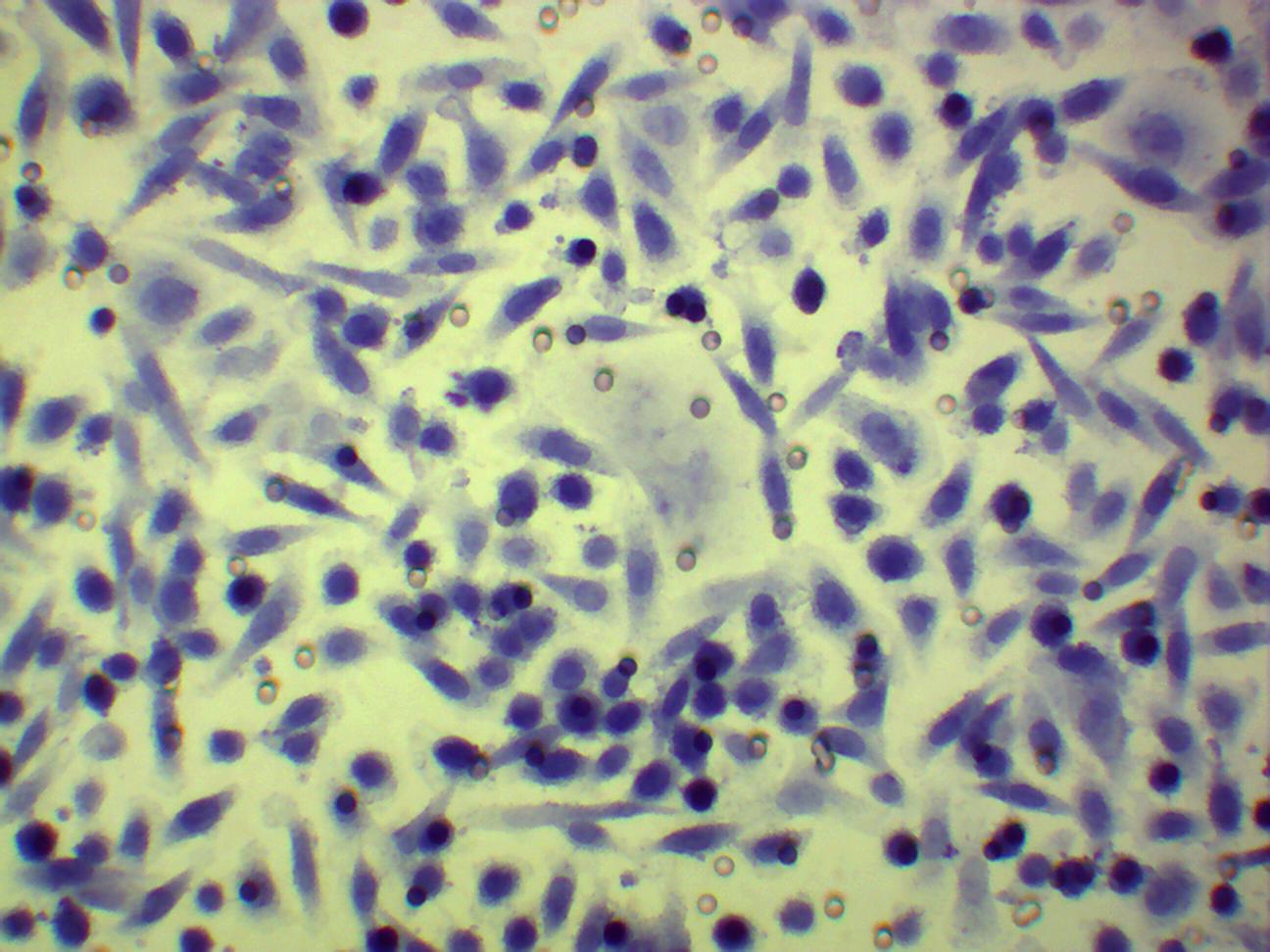

Supplement: Supplementary file 1 — Supplementary figures and tables. [file jcav14p2739s1.zip › supplementary/raw data/Figure 3/SW620/MNX1 1.JPG]

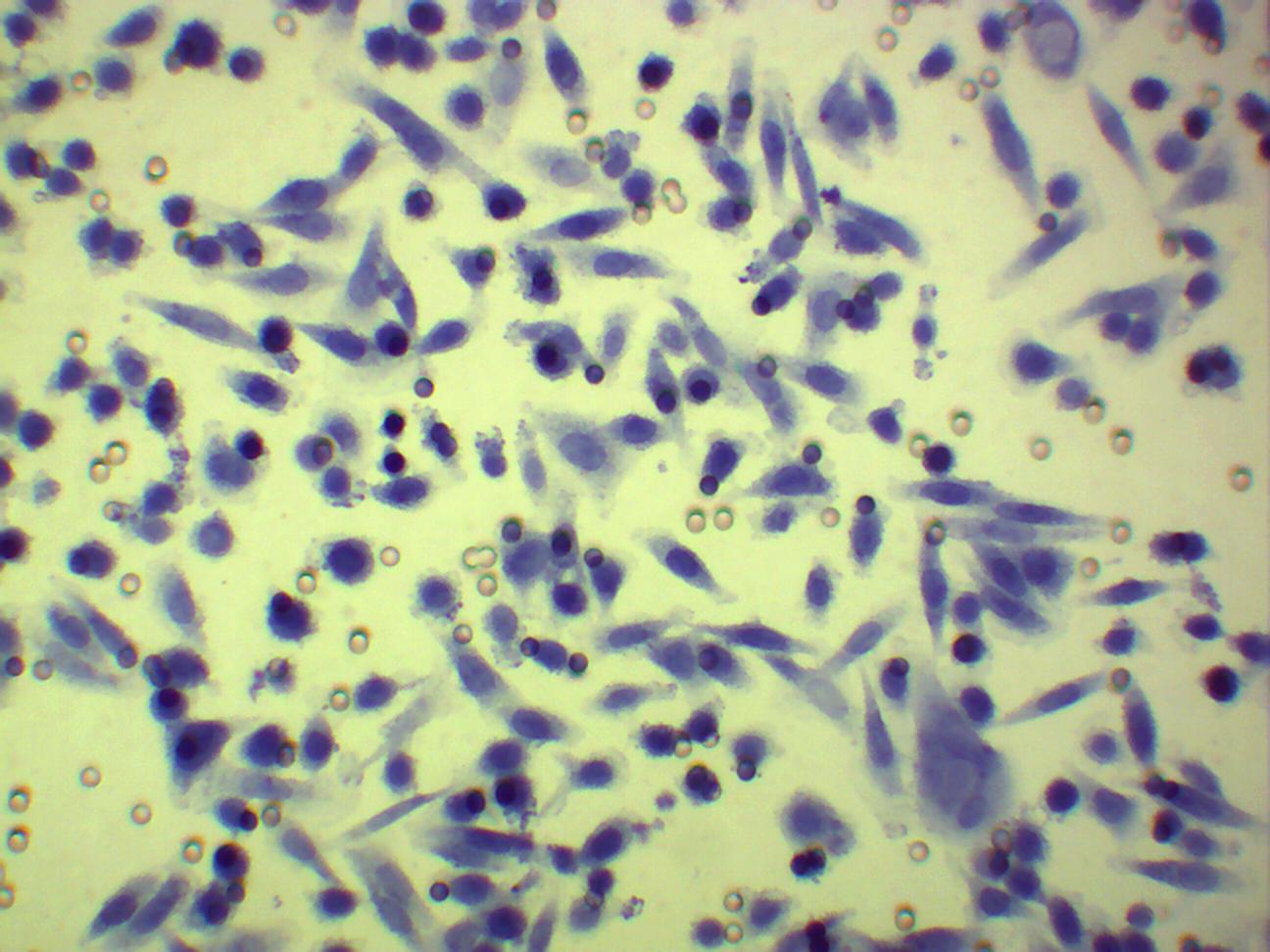

Supplement: Supplementary file 1 — Supplementary figures and tables. [file jcav14p2739s1.zip › supplementary/raw data/Figure 3/SW620/MNX1(1).JPG]

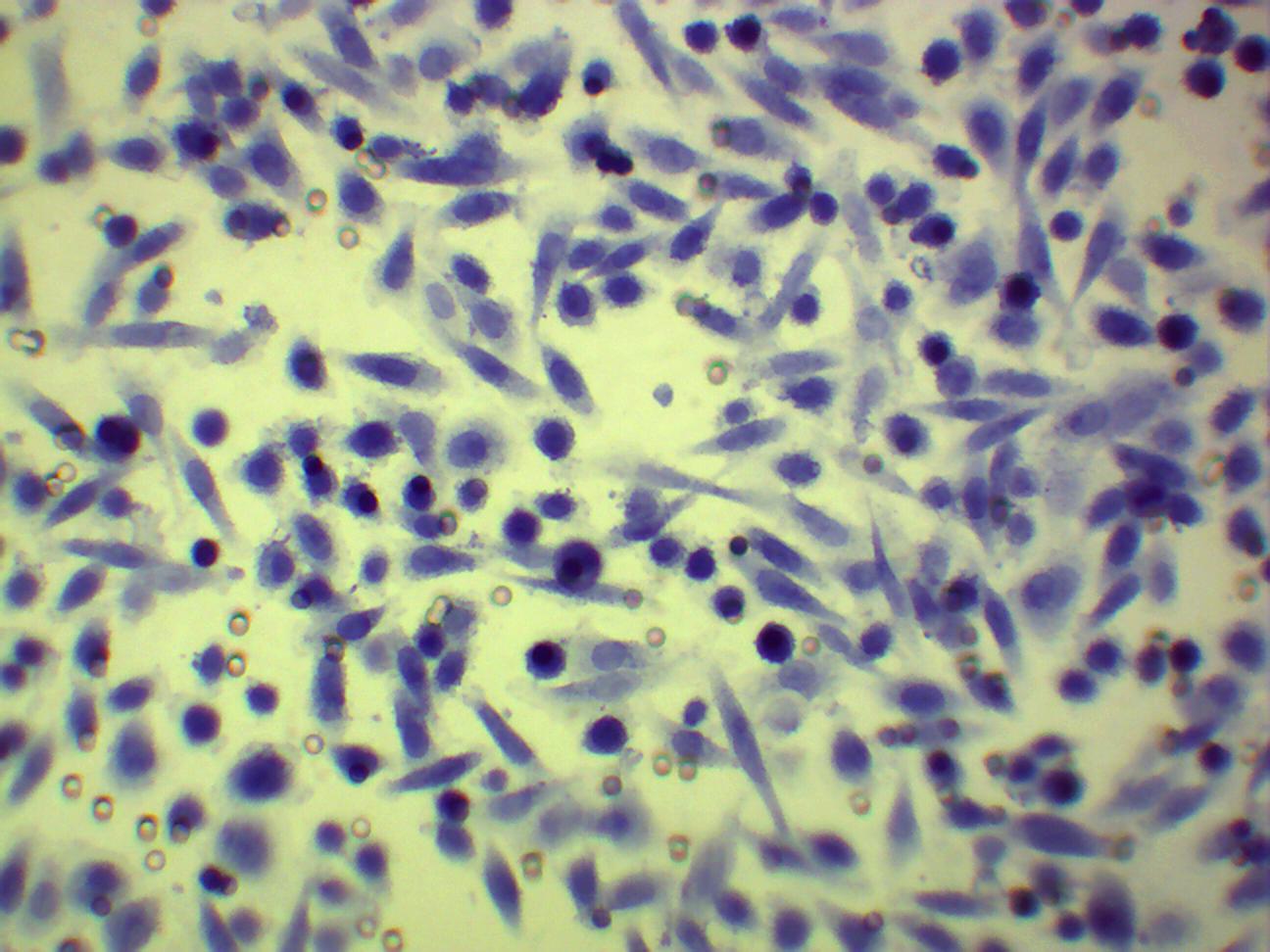

Supplement: Supplementary file 1 — Supplementary figures and tables. [file jcav14p2739s1.zip › supplementary/raw data/Figure 3/SW620/MNX1(2) (2).JPG]

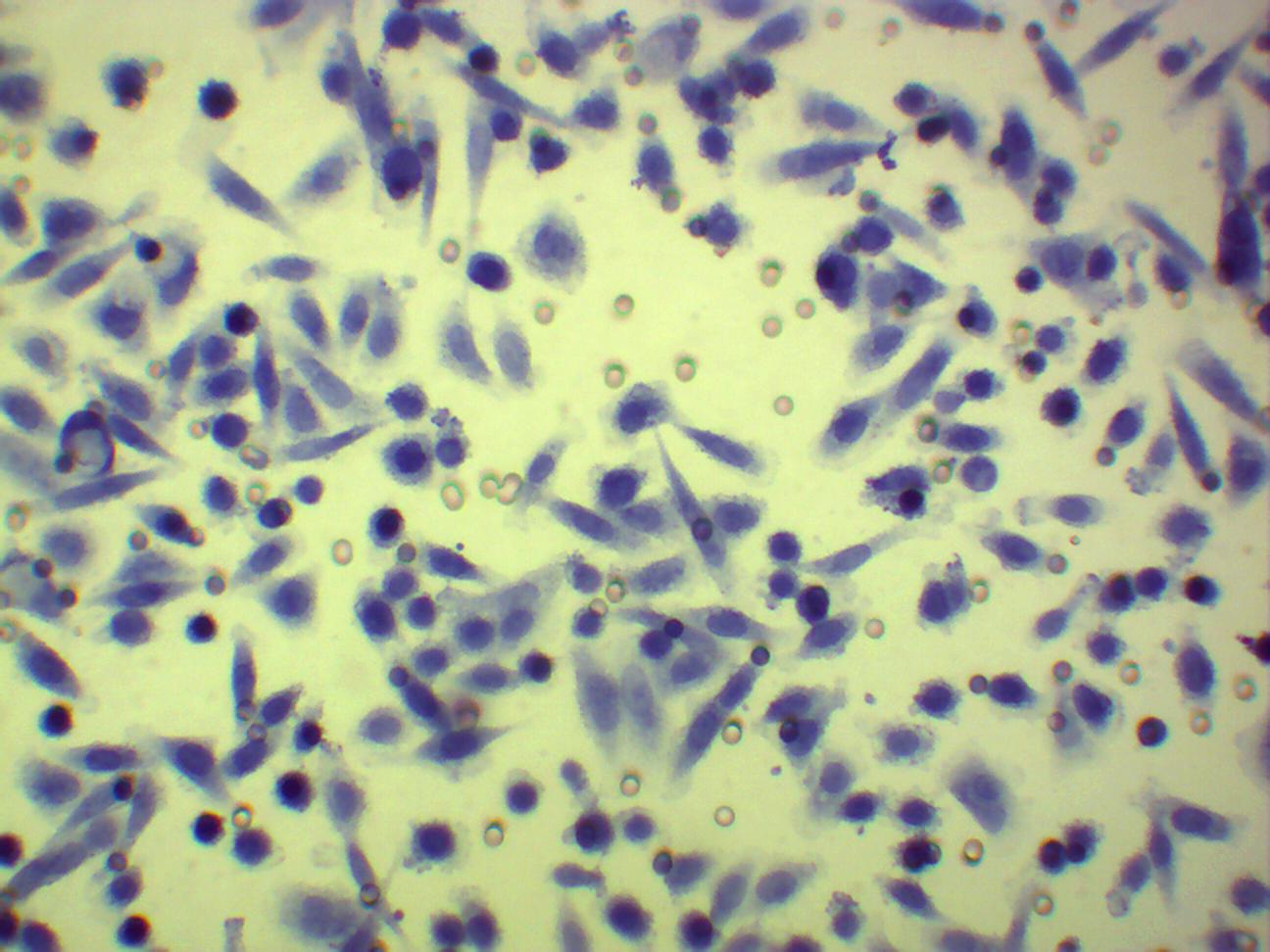

Supplement: Supplementary file 1 — Supplementary figures and tables. [file jcav14p2739s1.zip › supplementary/raw data/Figure 3/SW620/MNX1(2).JPG]

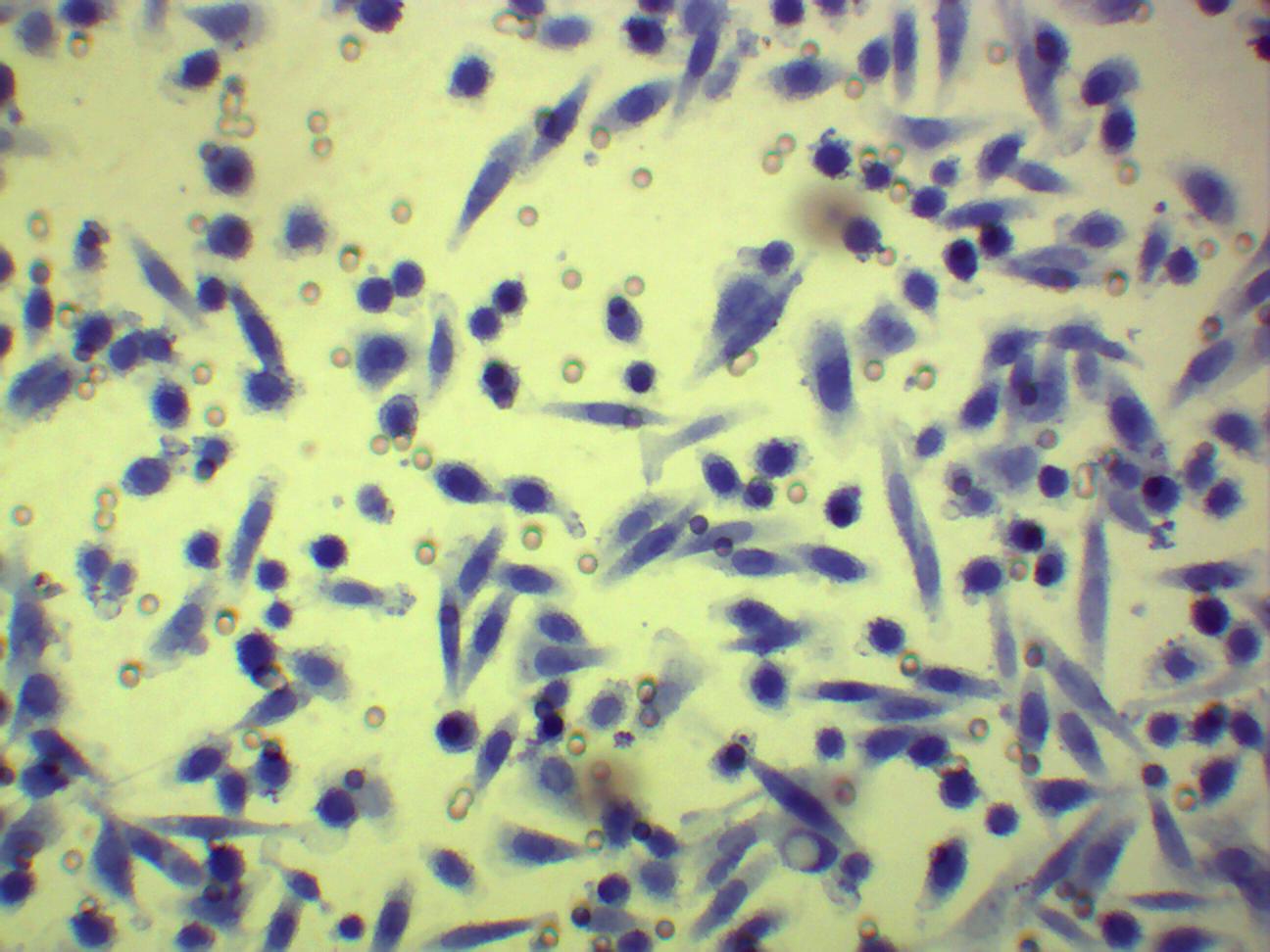

Supplement: Supplementary file 1 — Supplementary figures and tables. [file jcav14p2739s1.zip › supplementary/raw data/Figure 3/SW620/Vector2.JPG]

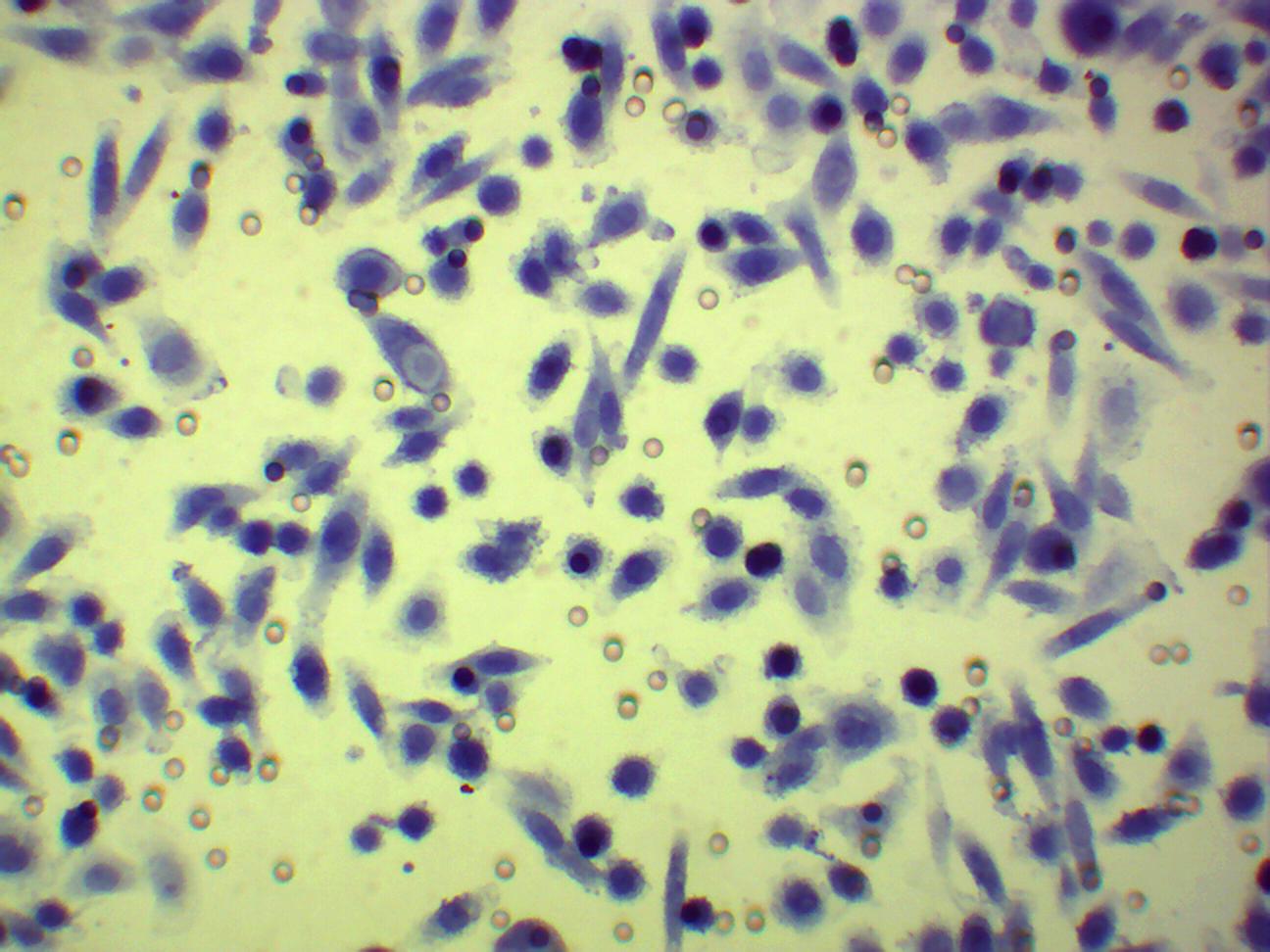

Supplement: Supplementary file 1 — Supplementary figures and tables. [file jcav14p2739s1.zip › supplementary/raw data/Figure 3/SW620/Vector(1) .JPG]

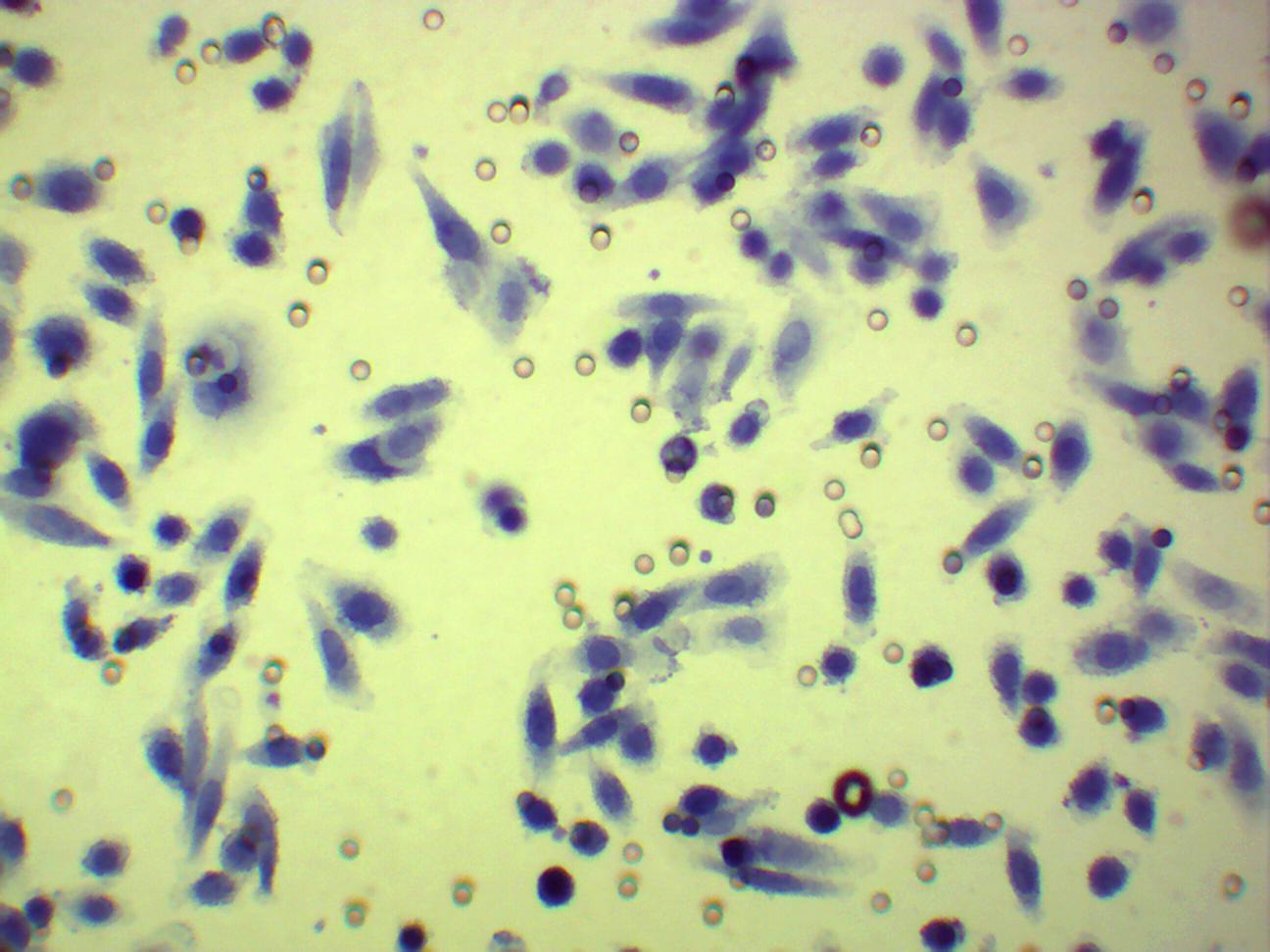

Supplement: Supplementary file 1 — Supplementary figures and tables. [file jcav14p2739s1.zip › supplementary/raw data/Figure 3/SW620/Vector(1).JPG]

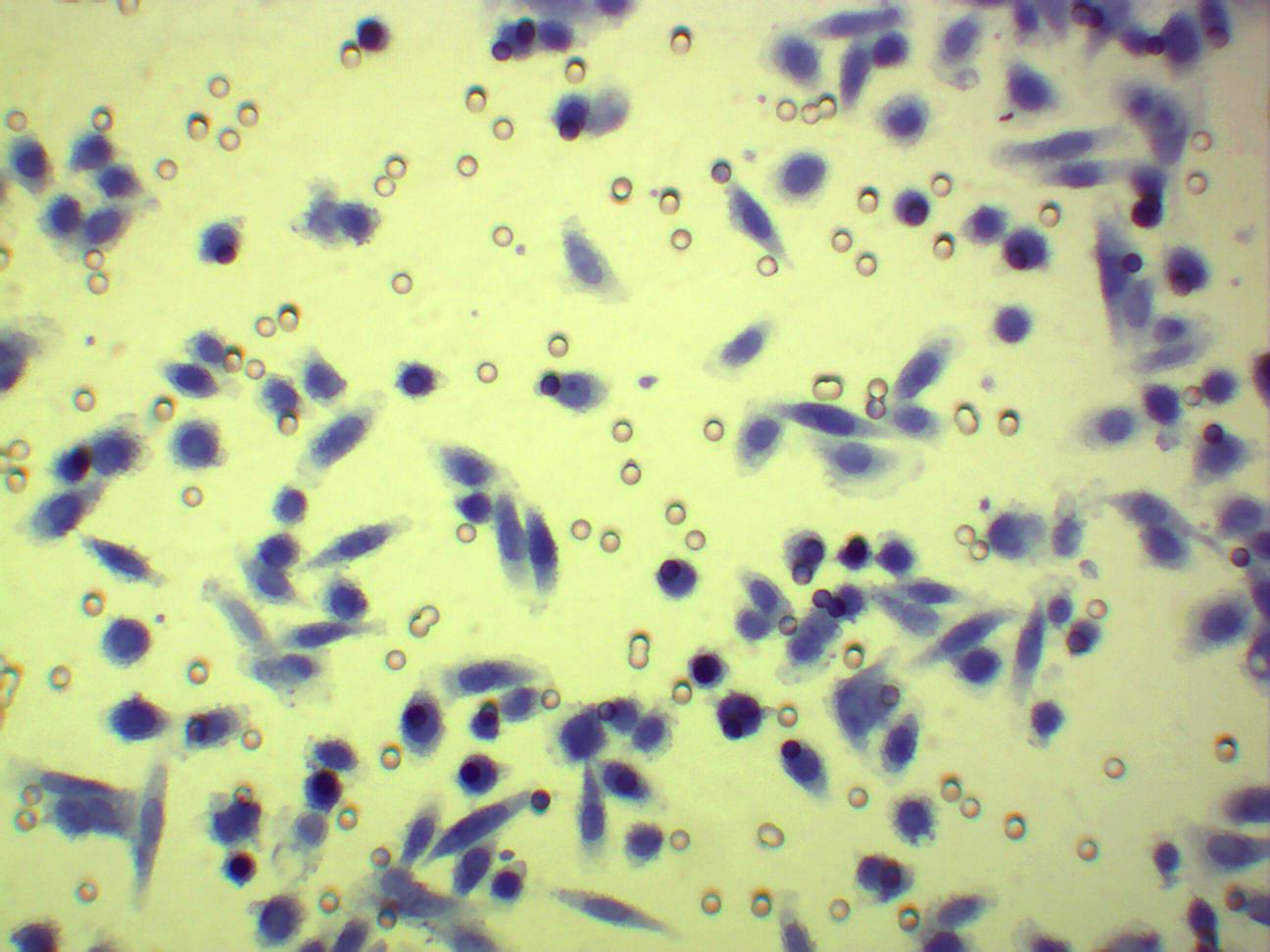

Supplement: Supplementary file 1 — Supplementary figures and tables. [file jcav14p2739s1.zip › supplementary/raw data/Figure 3/SW620/Vector(2).JPG]

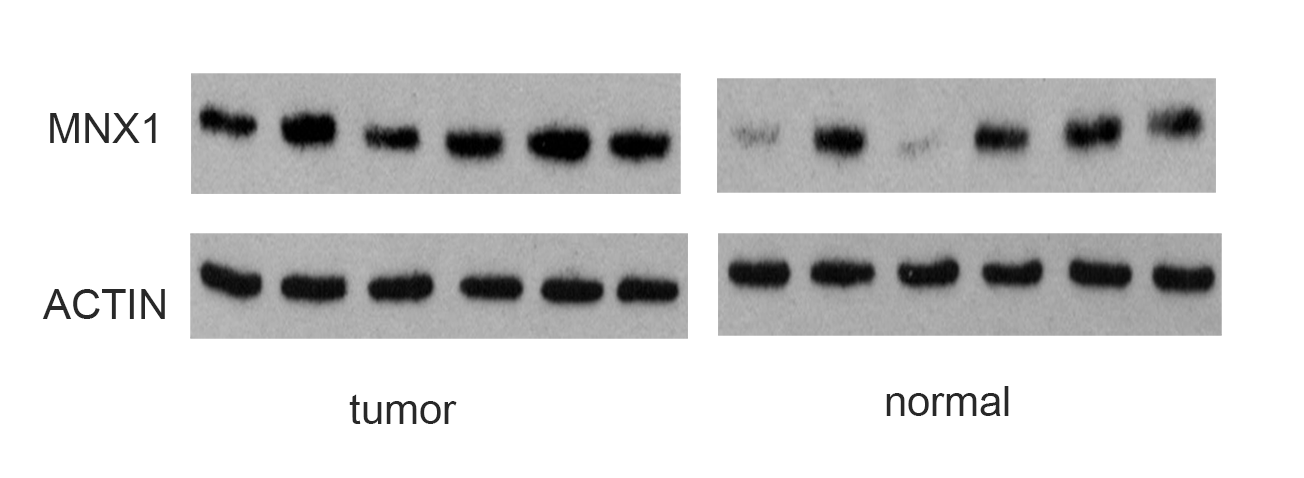

Supplement: Supplementary file 1 — Supplementary figures and tables. [file jcav14p2739s1.zip › supplementary/raw data/Figure 4/f4f repeat.tif]

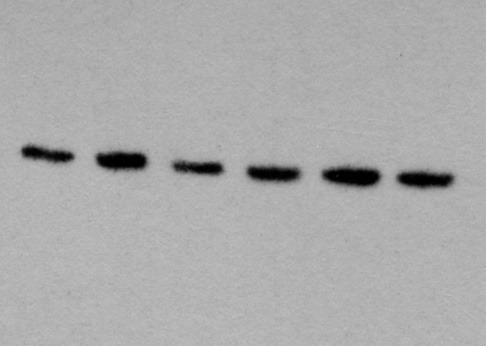

Supplement: Supplementary file 1 — Supplementary figures and tables. [file jcav14p2739s1.zip › supplementary/raw data/Figure 4/f4h-1.tif]

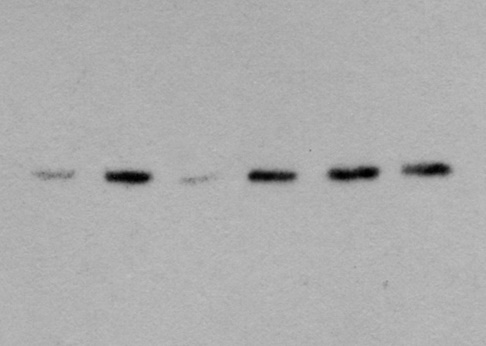

Supplement: Supplementary file 1 — Supplementary figures and tables. [file jcav14p2739s1.zip › supplementary/raw data/Figure 4/f4h-2.tif]

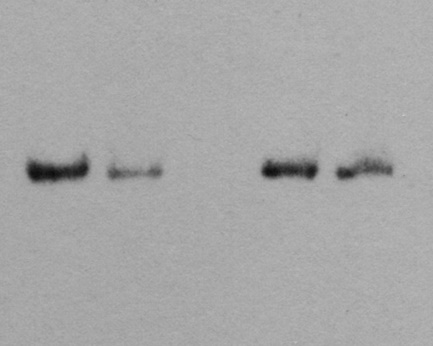

Supplement: Supplementary file 1 — Supplementary figures and tables. [file jcav14p2739s1.zip › supplementary/raw data/Figure 4/f4j-1.tif]

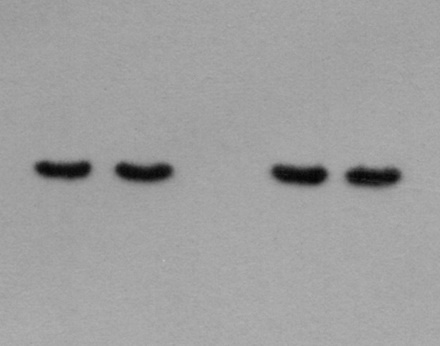

Supplement: Supplementary file 1 — Supplementary figures and tables. [file jcav14p2739s1.zip › supplementary/raw data/Figure 4/f4j-1b.tif]

E2F4 vs. MNX1, 331 samples (COAD), Pearson's  $r = 0.4564$ ,  $p\text{-value} = 1.97\text{e-}18$

Data Source: ChIPBase v2.0 project

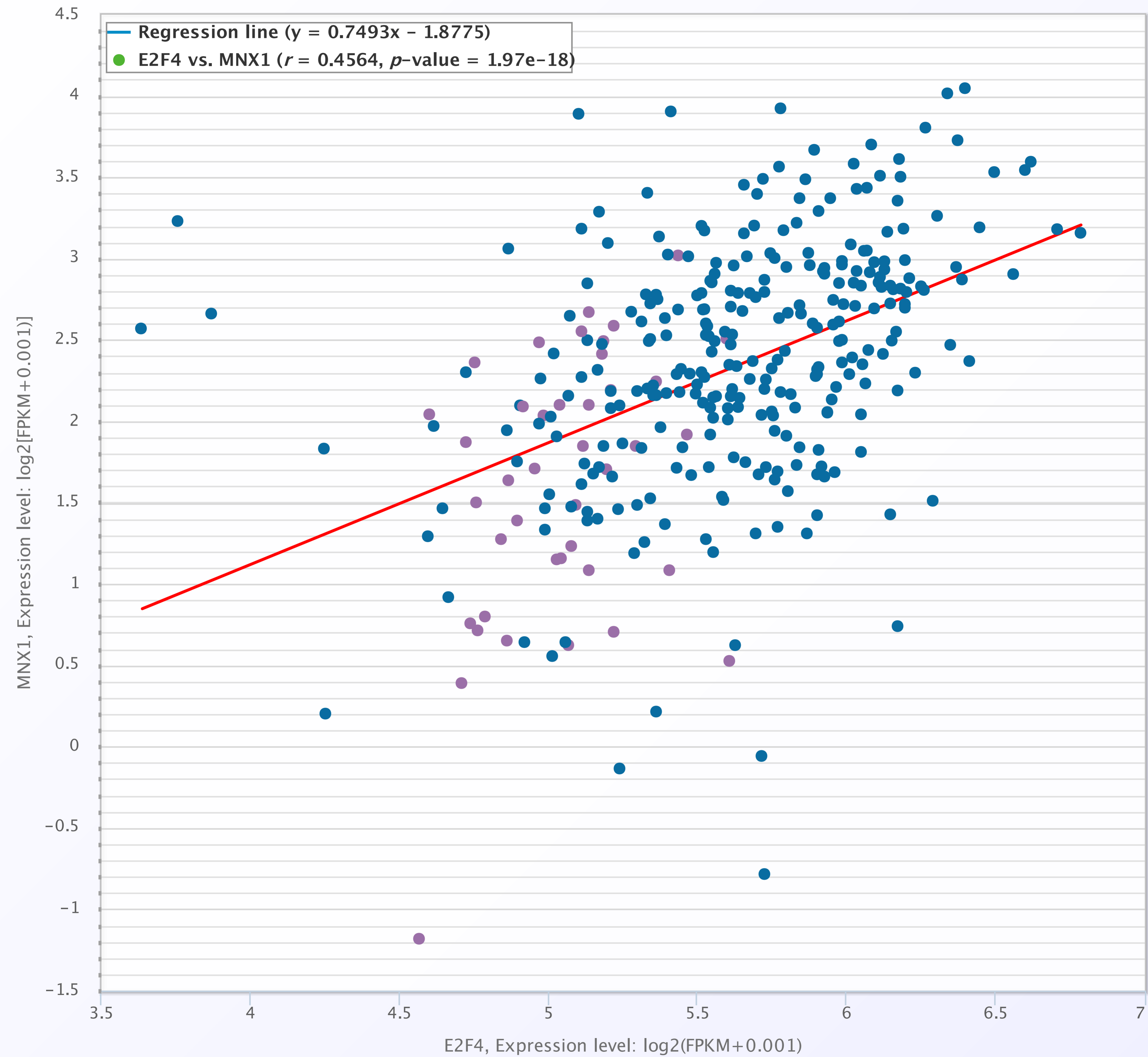

Supplement: Supplementary file 1 — Supplementary figures and tables. [file jcav14p2739s1.zip › supplementary/raw data/Figure 4/Fig B.pdf]

# E2F4 with 471 cancer and 41 normal samples in COAD

Data Source: starBase v3.0 project

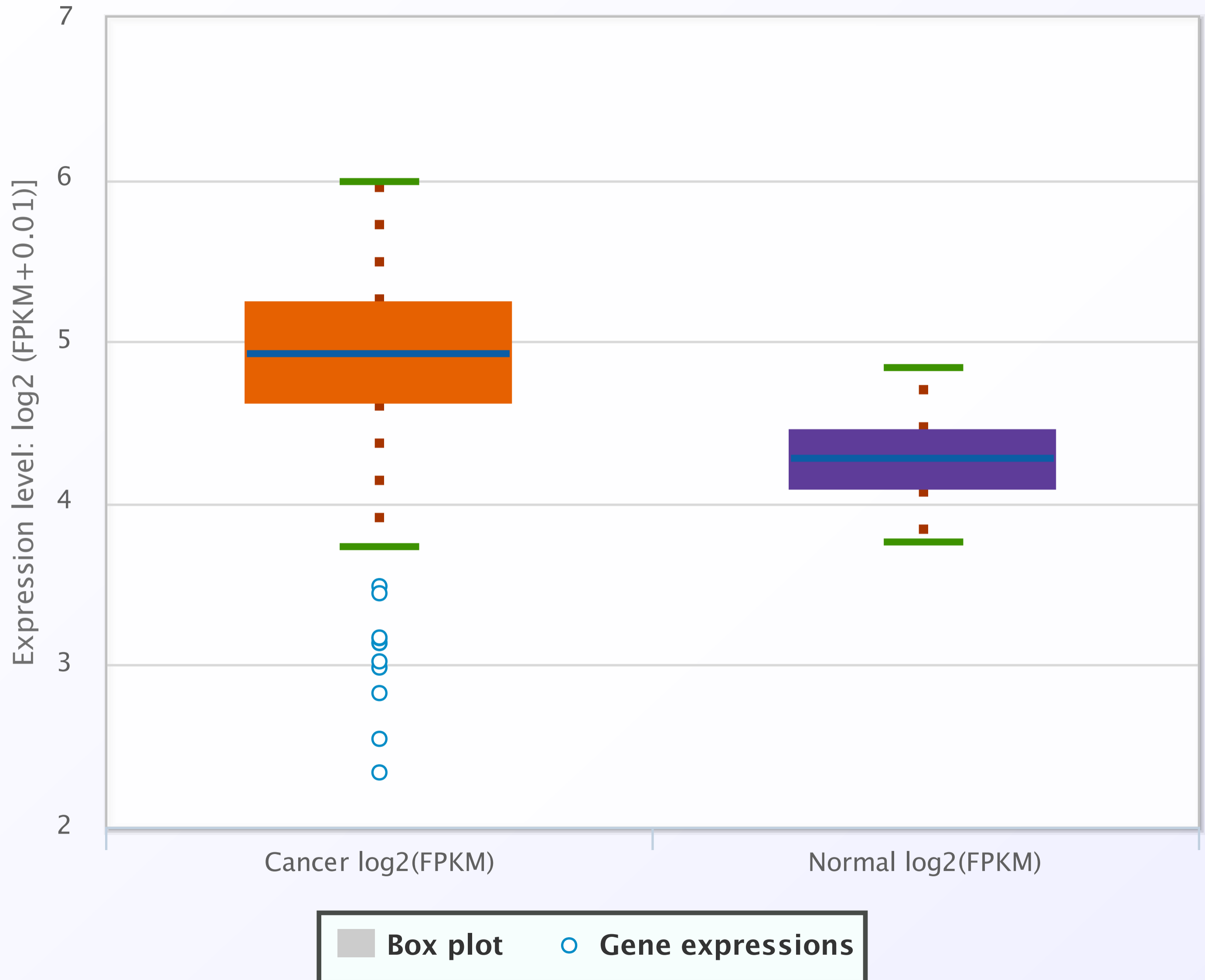

Supplement: Supplementary file 1 — Supplementary figures and tables. [file jcav14p2739s1.zip › supplementary/raw data/Figure 4/Fig C.pdf]

# Overall Survival

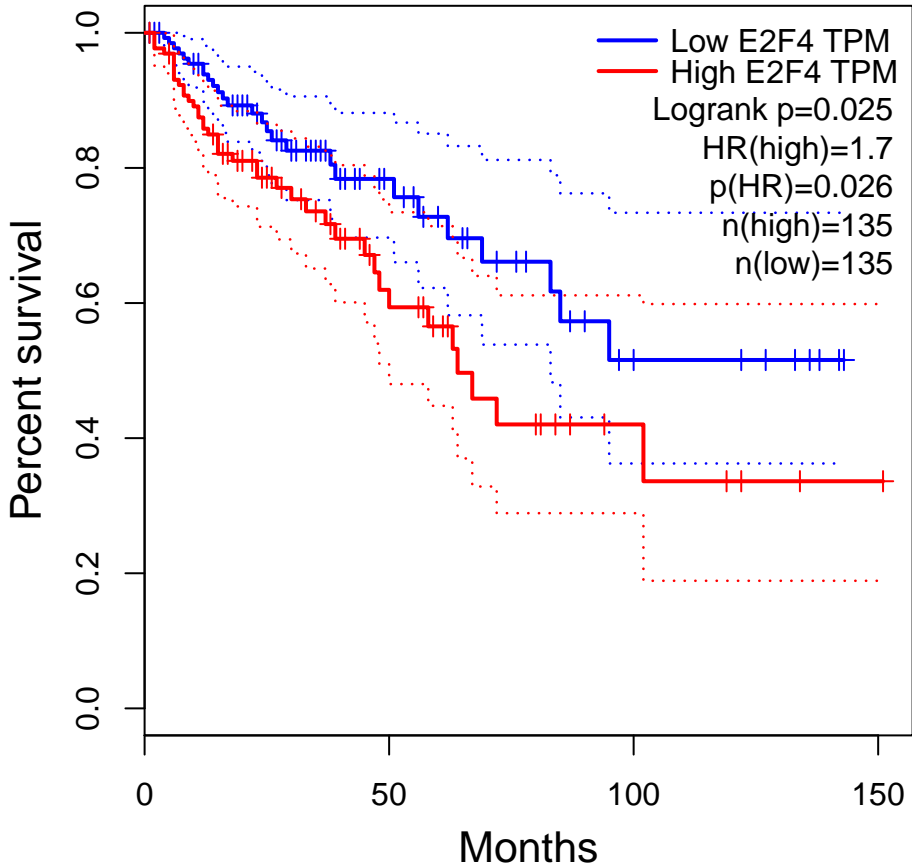

Supplement: Supplementary file 1 — Supplementary figures and tables. [file jcav14p2739s1.zip › supplementary/raw data/Figure 4/Fig D.pdf]

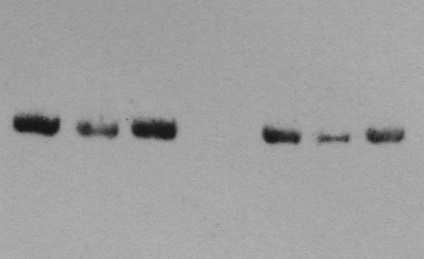

Supplement: Supplementary file 1 — Supplementary figures and tables. [file jcav14p2739s1.zip › supplementary/raw data/Figure 5/f5b-1.tif]

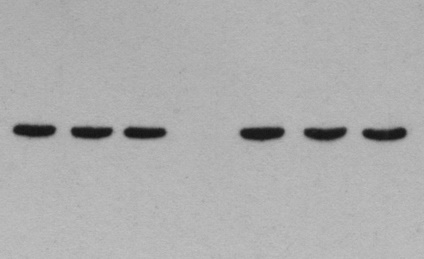

Supplement: Supplementary file 1 — Supplementary figures and tables. [file jcav14p2739s1.zip › supplementary/raw data/Figure 5/f5b-1b.tif]

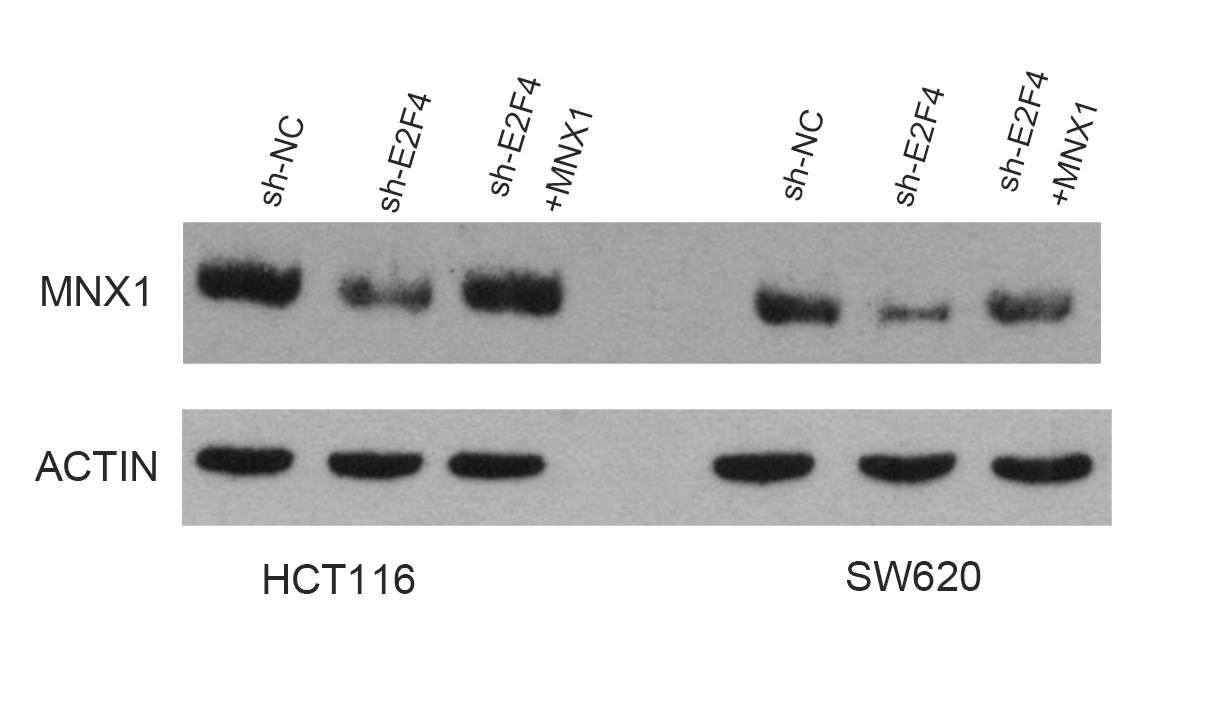

Supplement: Supplementary file 1 — Supplementary figures and tables. [file jcav14p2739s1.zip › supplementary/raw data/Figure 5/F5B-repeat.tif]

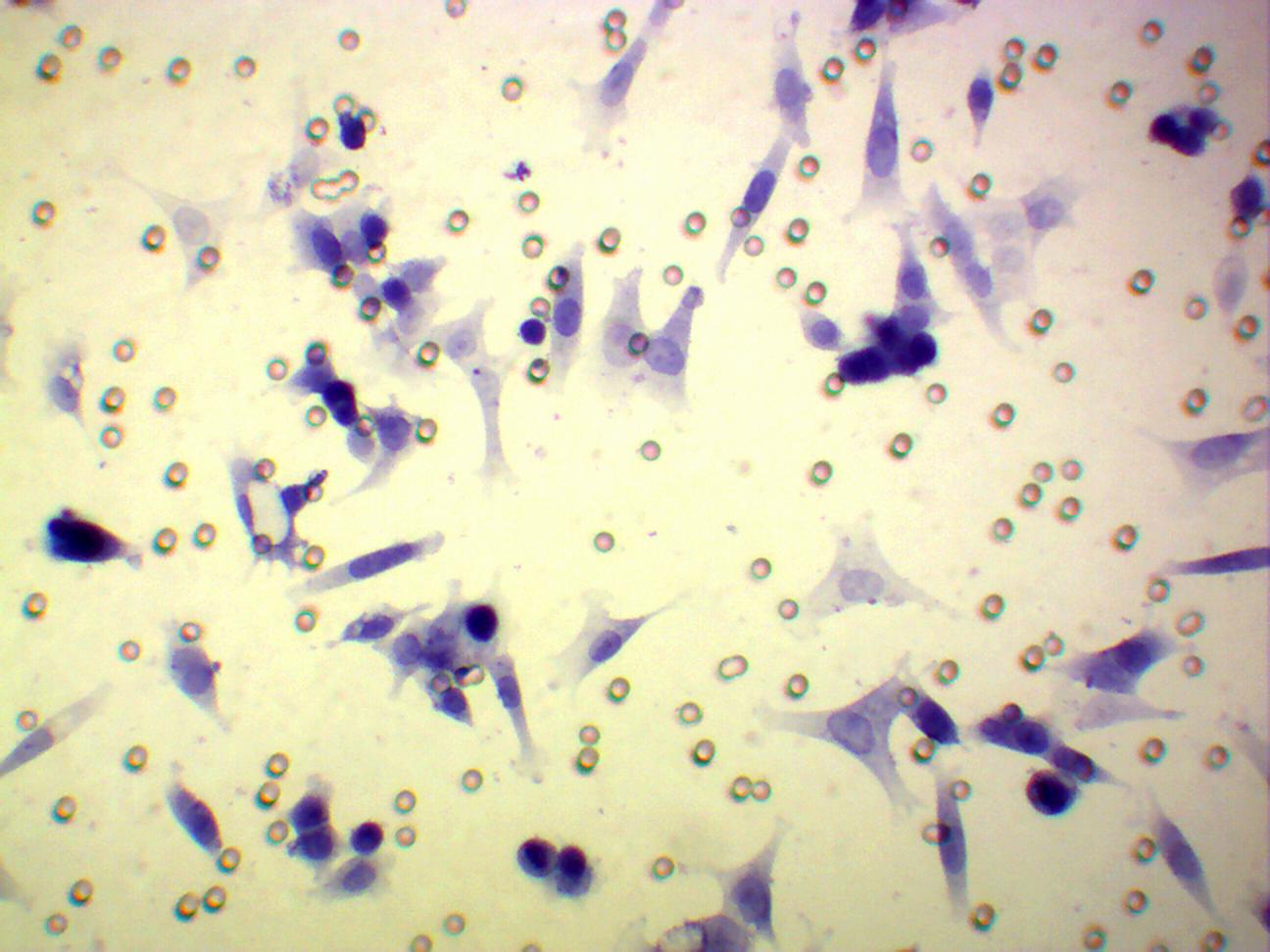

Supplement: Supplementary file 1 — Supplementary figures and tables. [file jcav14p2739s1.zip › supplementary/raw data/Figure 5/HCT116/E2F4 (1).JPG]

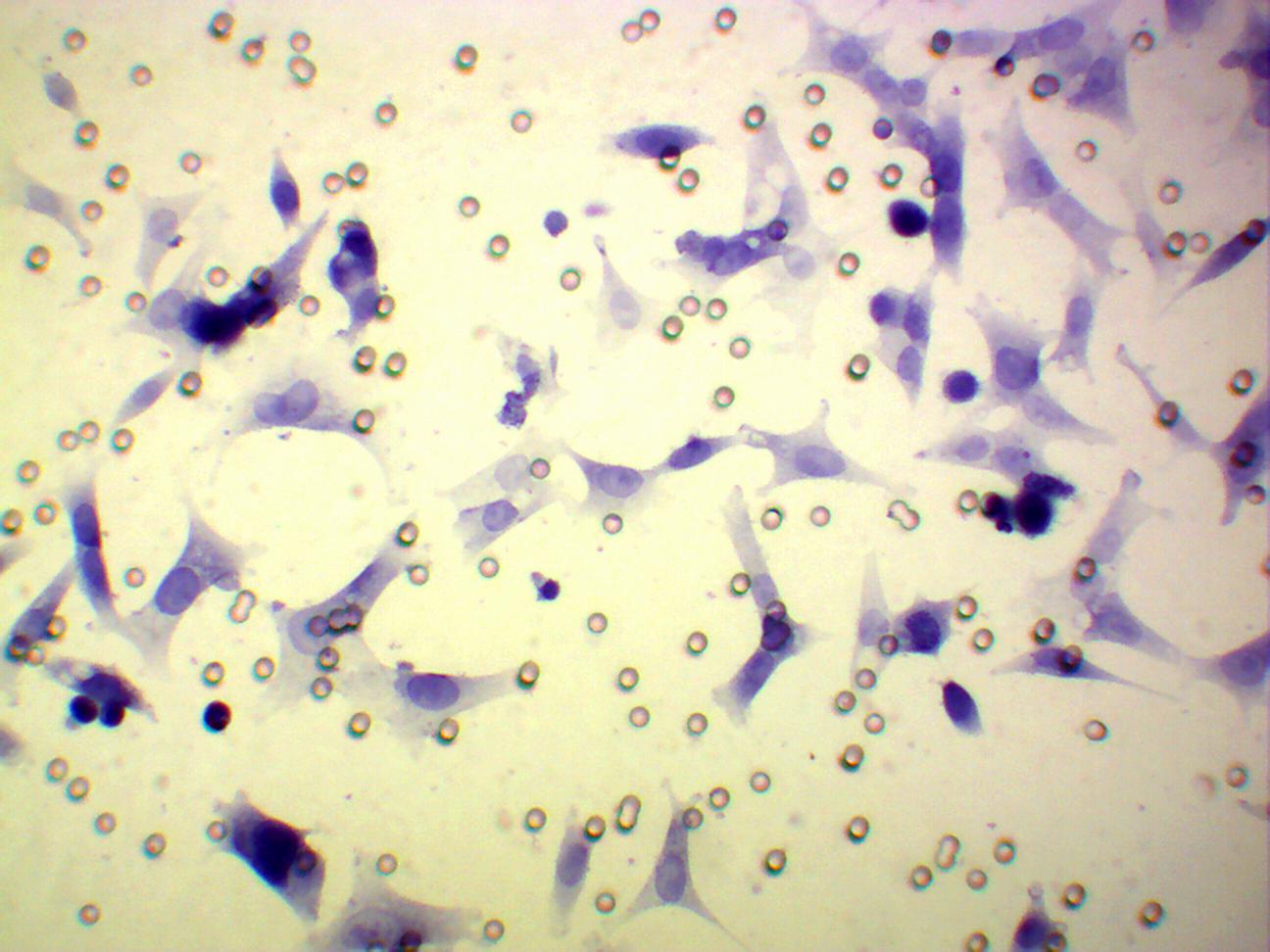

Supplement: Supplementary file 1 — Supplementary figures and tables. [file jcav14p2739s1.zip › supplementary/raw data/Figure 5/HCT116/E2F4 (2).JPG]

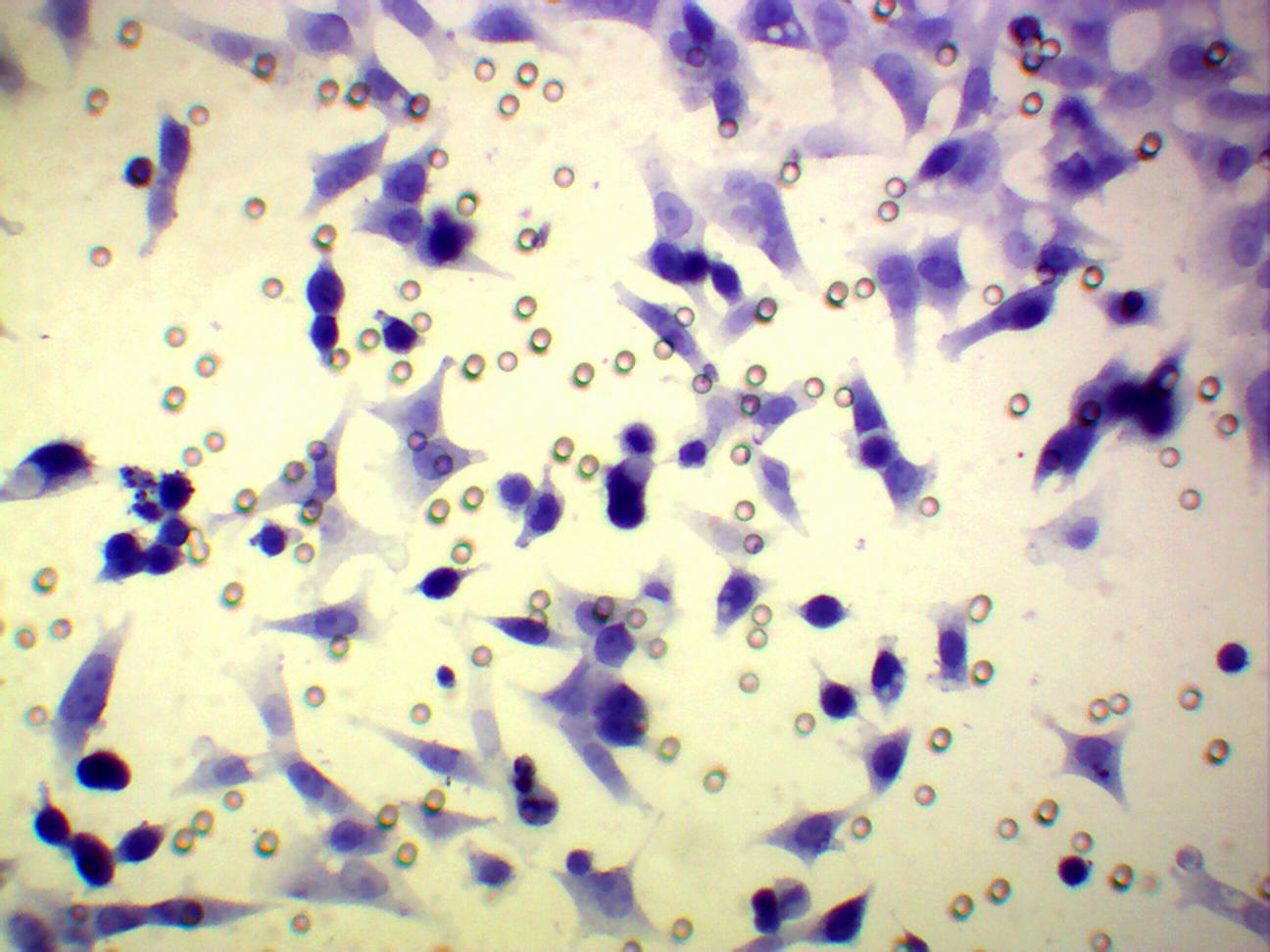

Supplement: Supplementary file 1 — Supplementary figures and tables. [file jcav14p2739s1.zip › supplementary/raw data/Figure 5/HCT116/E2F4+MNX1 (1).JPG]

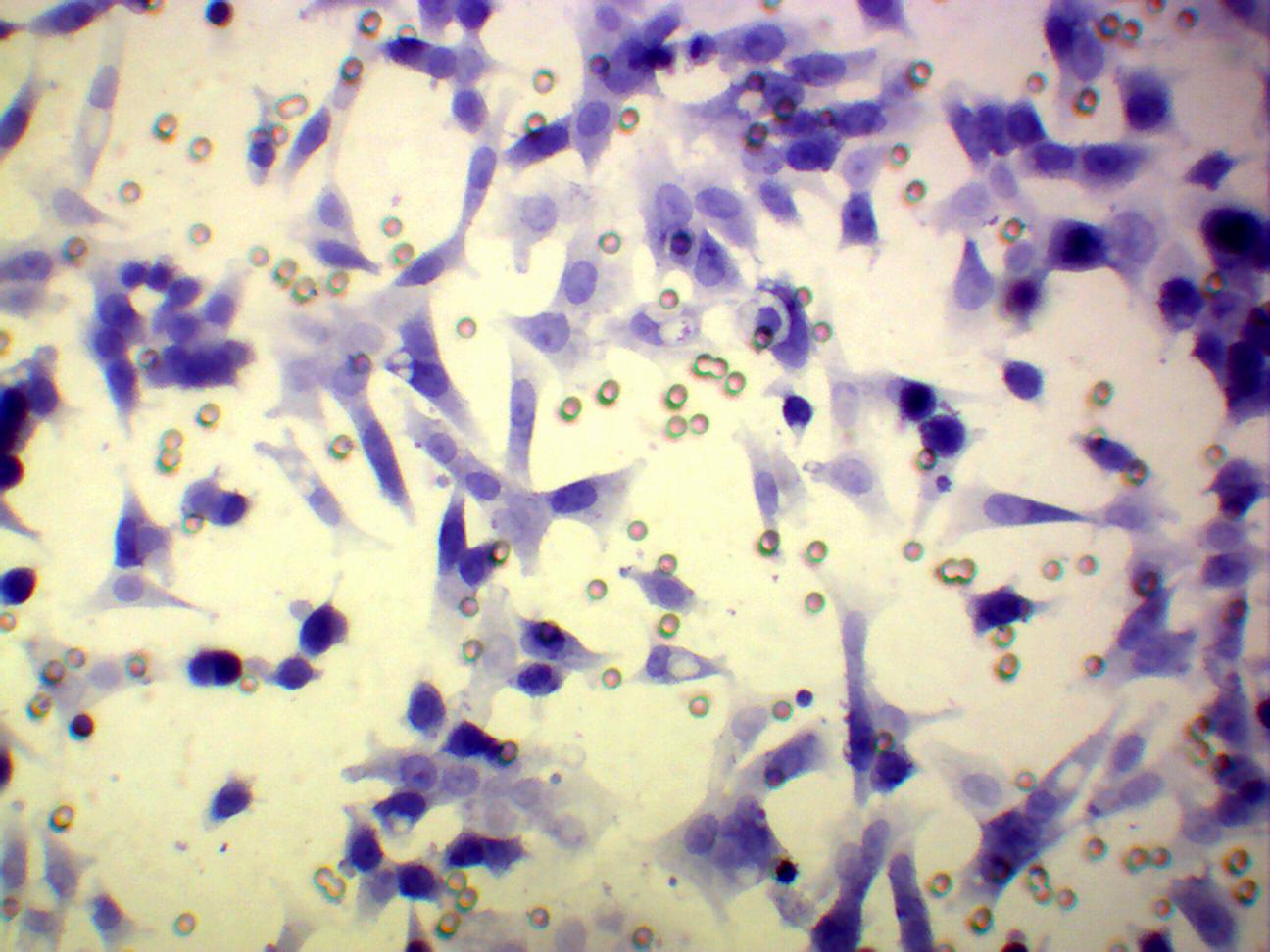

Supplement: Supplementary file 1 — Supplementary figures and tables. [file jcav14p2739s1.zip › supplementary/raw data/Figure 5/HCT116/E2F4+MNX1(1).JPG]

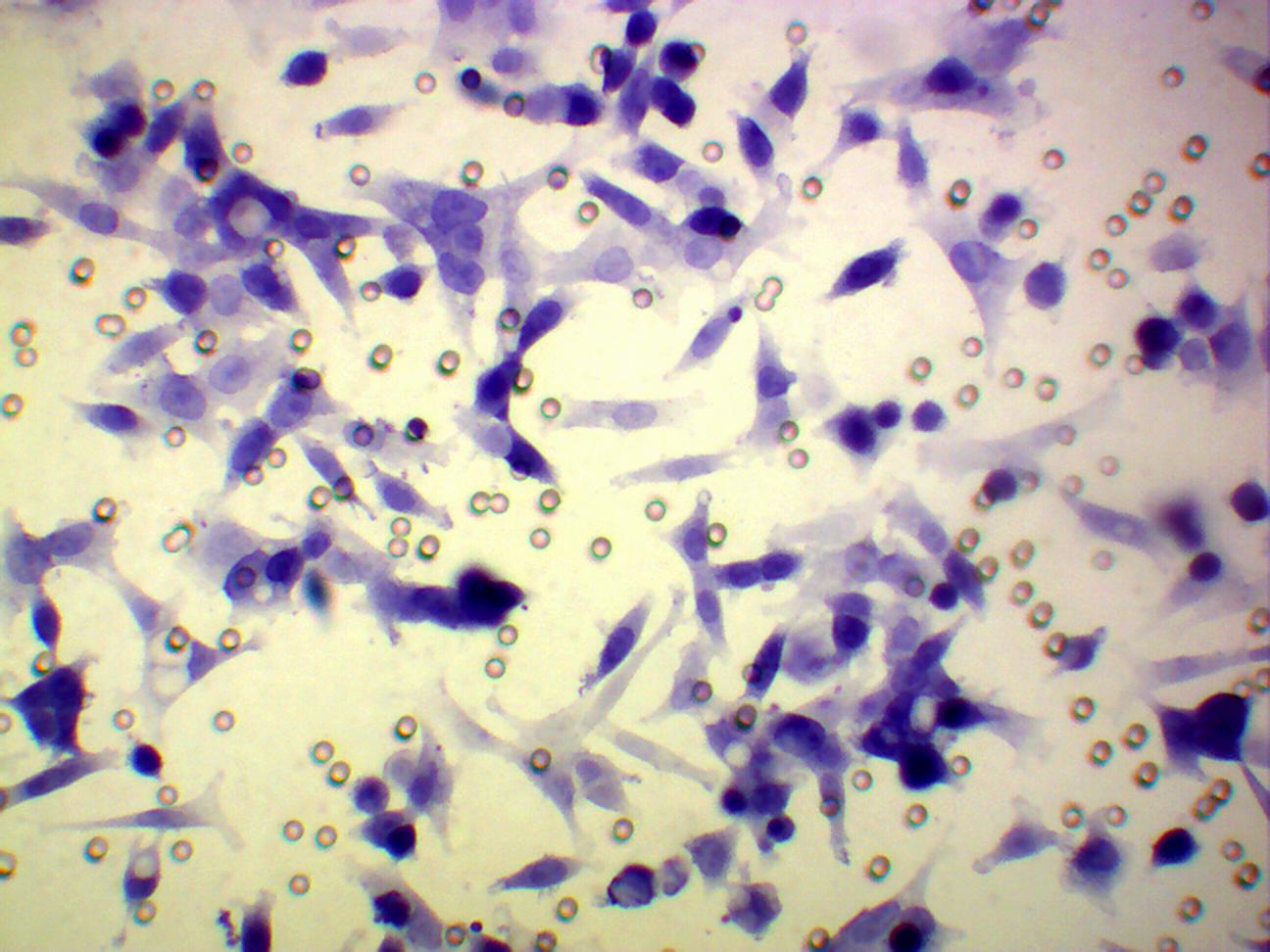

Supplement: Supplementary file 1 — Supplementary figures and tables. [file jcav14p2739s1.zip › supplementary/raw data/Figure 5/HCT116/E2F4+MNX1(2) .JPG]

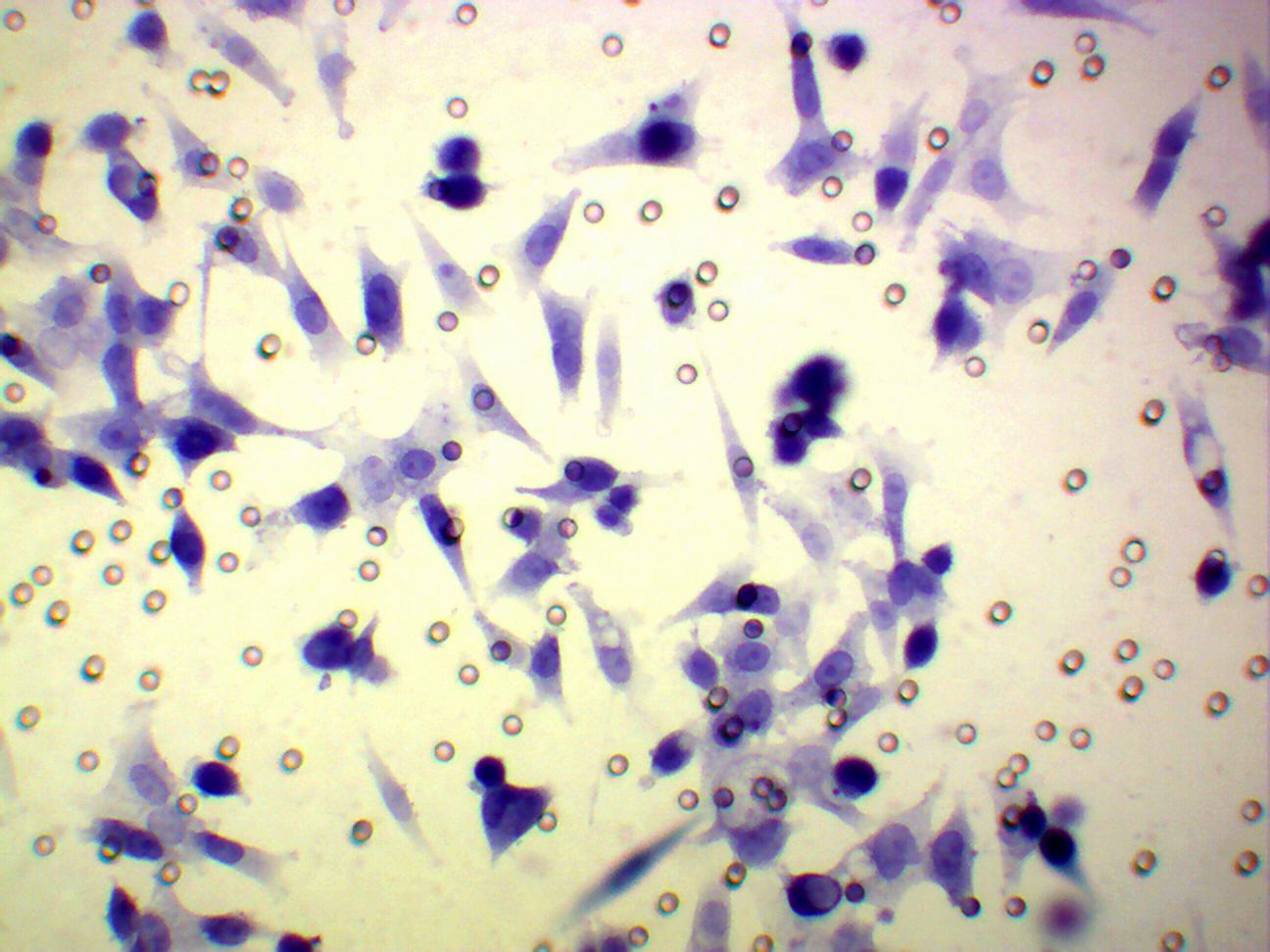

Supplement: Supplementary file 1 — Supplementary figures and tables. [file jcav14p2739s1.zip › supplementary/raw data/Figure 5/HCT116/E2F4+MNX1(2).JPG]

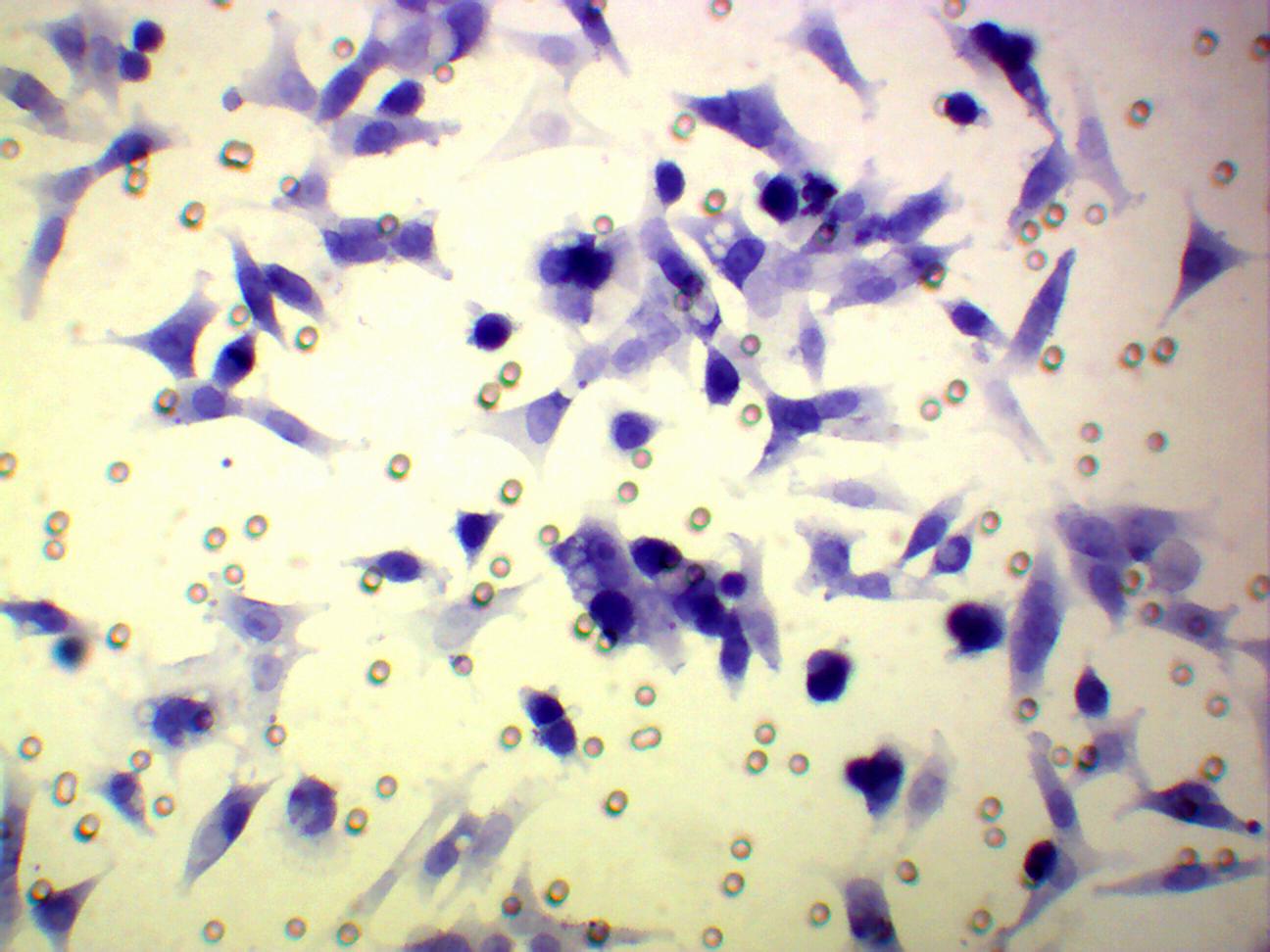

Supplement: Supplementary file 1 — Supplementary figures and tables. [file jcav14p2739s1.zip › supplementary/raw data/Figure 5/HCT116/E2F4(1).JPG]

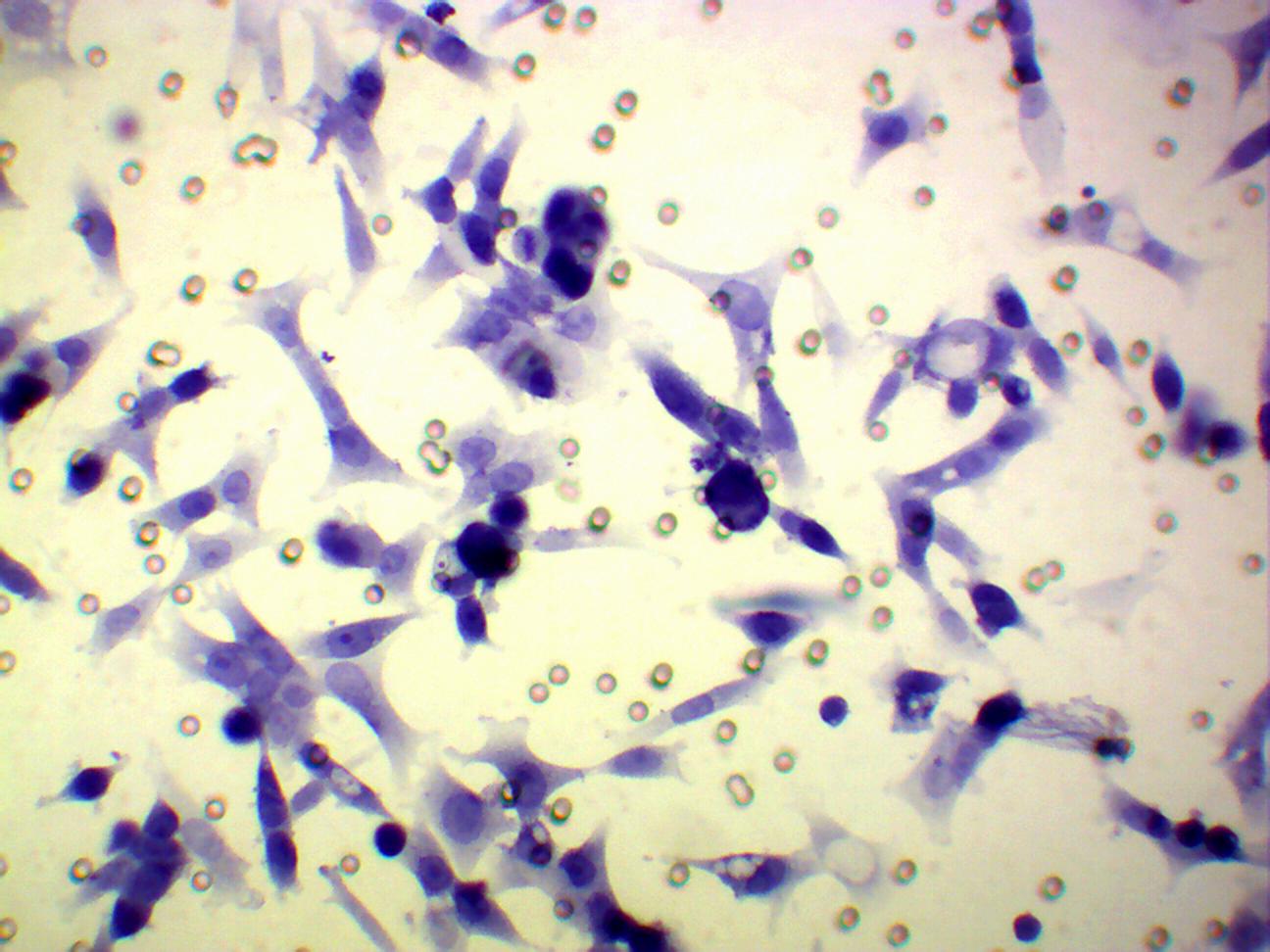

Supplement: Supplementary file 1 — Supplementary figures and tables. [file jcav14p2739s1.zip › supplementary/raw data/Figure 5/HCT116/E2F4(2).JPG]

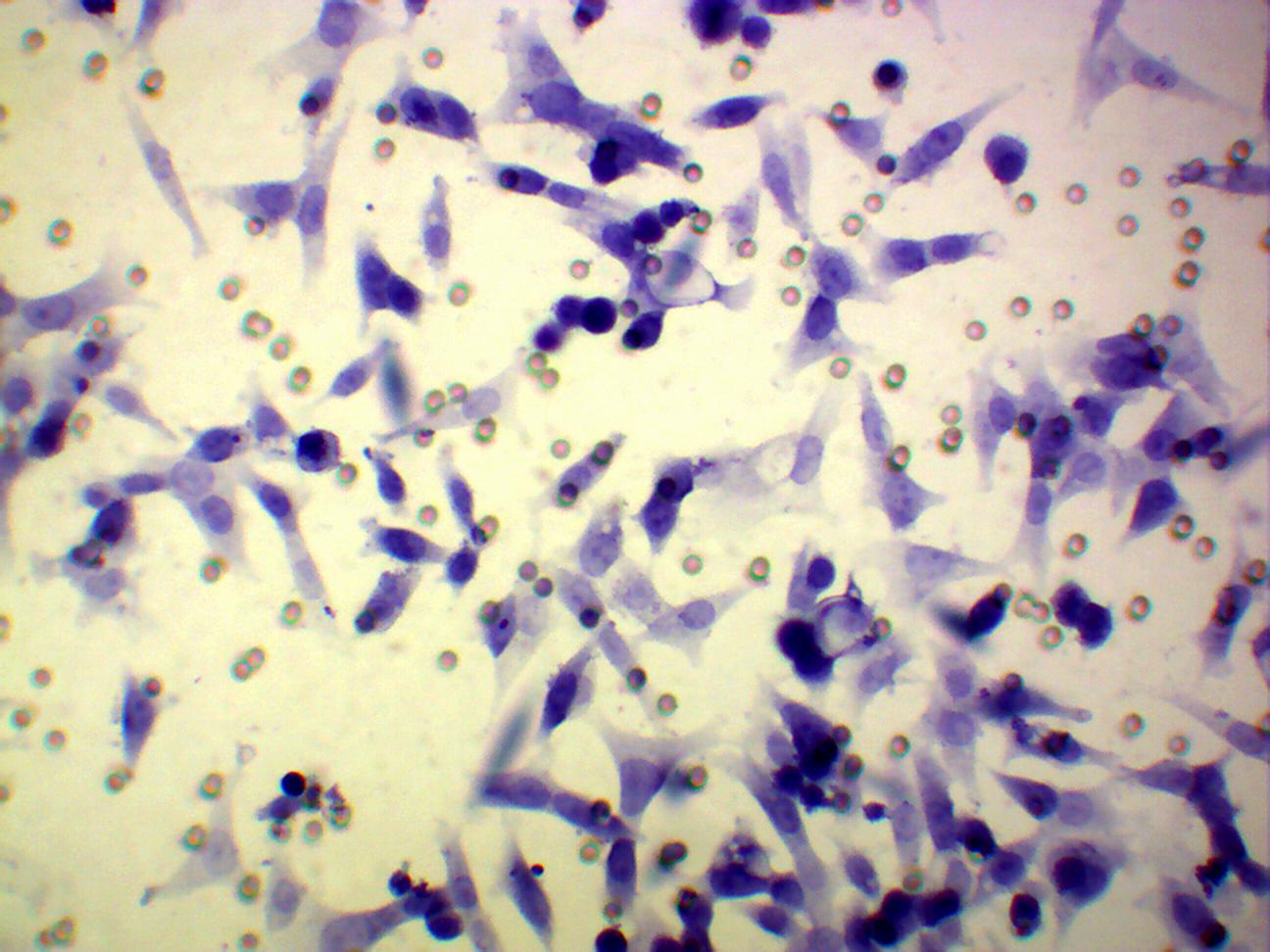

Supplement: Supplementary file 1 — Supplementary figures and tables. [file jcav14p2739s1.zip › supplementary/raw data/Figure 5/HCT116/NC (2).JPG]

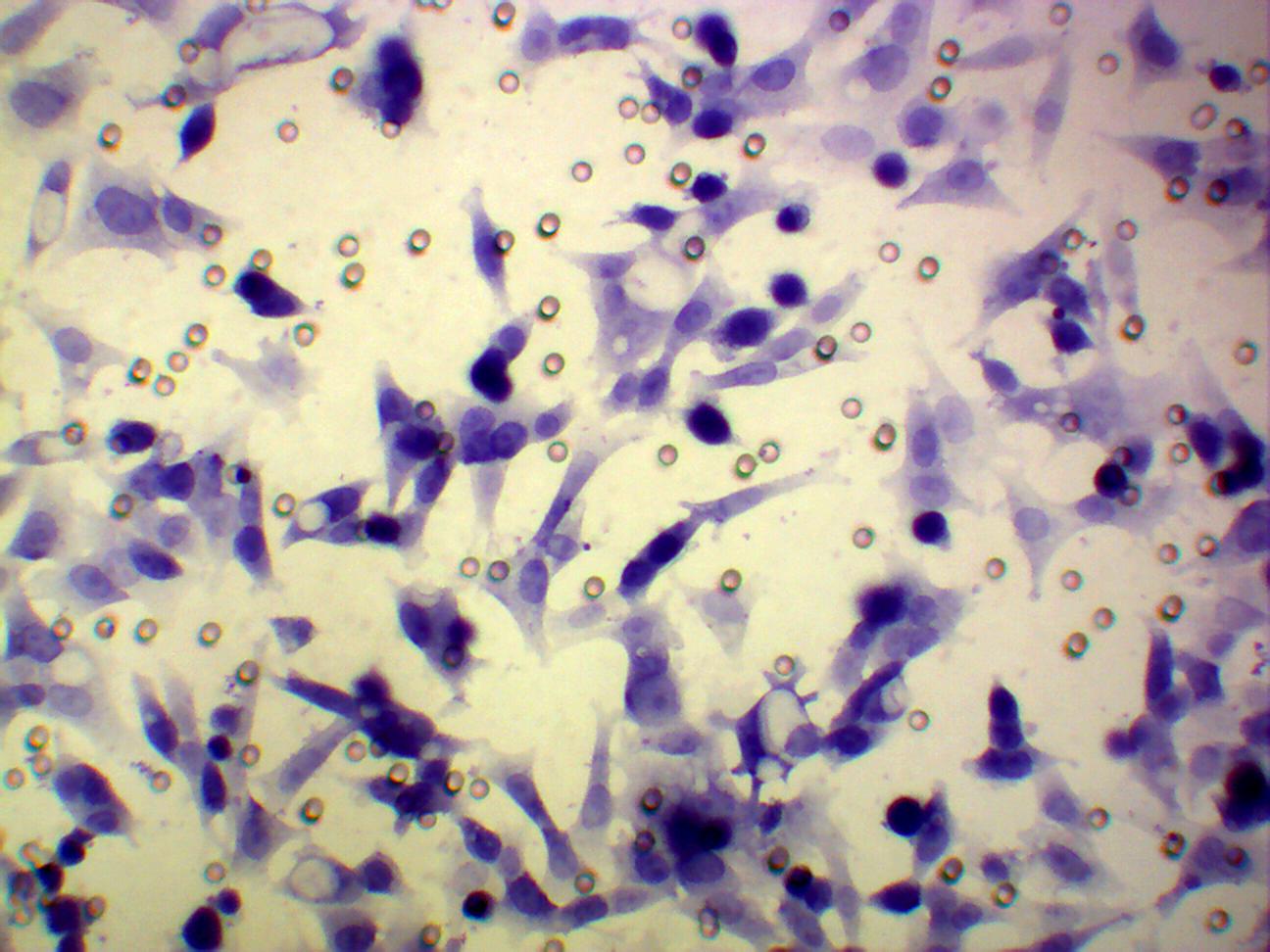

Supplement: Supplementary file 1 — Supplementary figures and tables. [file jcav14p2739s1.zip › supplementary/raw data/Figure 5/HCT116/NC(1).JPG]

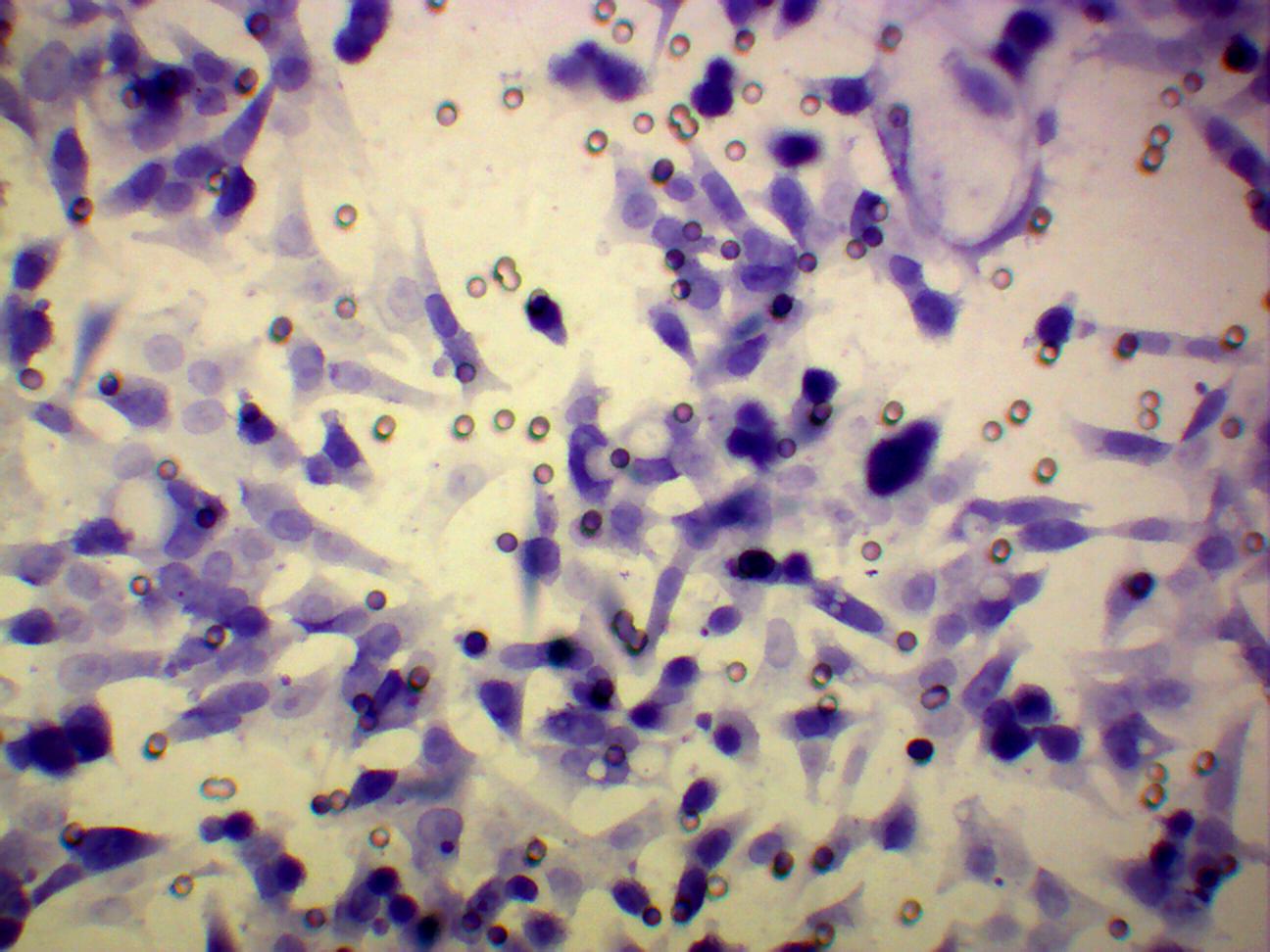

Supplement: Supplementary file 1 — Supplementary figures and tables. [file jcav14p2739s1.zip › supplementary/raw data/Figure 5/HCT116/NC(1).JPG]

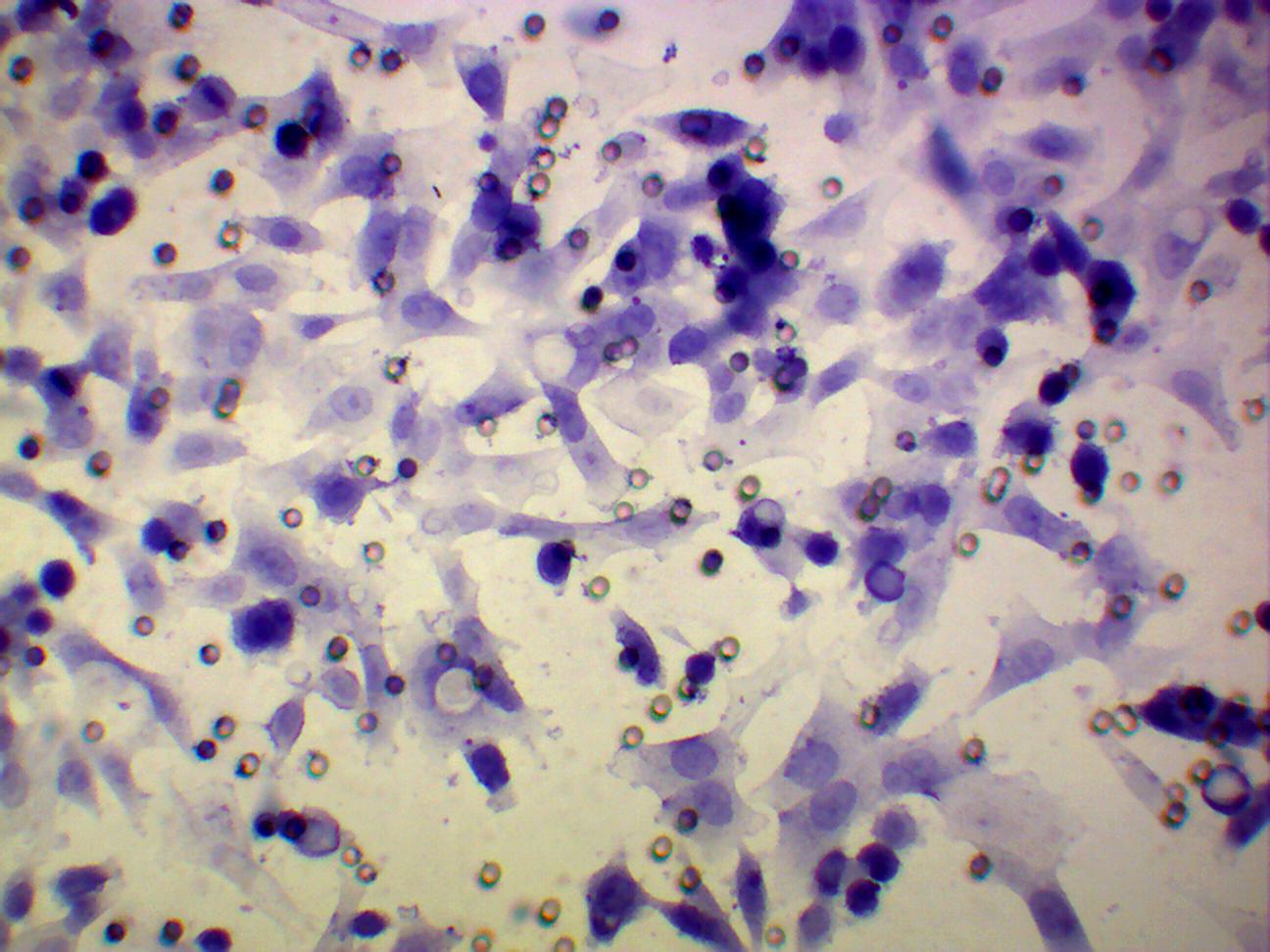

Supplement: Supplementary file 1 — Supplementary figures and tables. [file jcav14p2739s1.zip › supplementary/raw data/Figure 5/HCT116/NC(2).JPG]

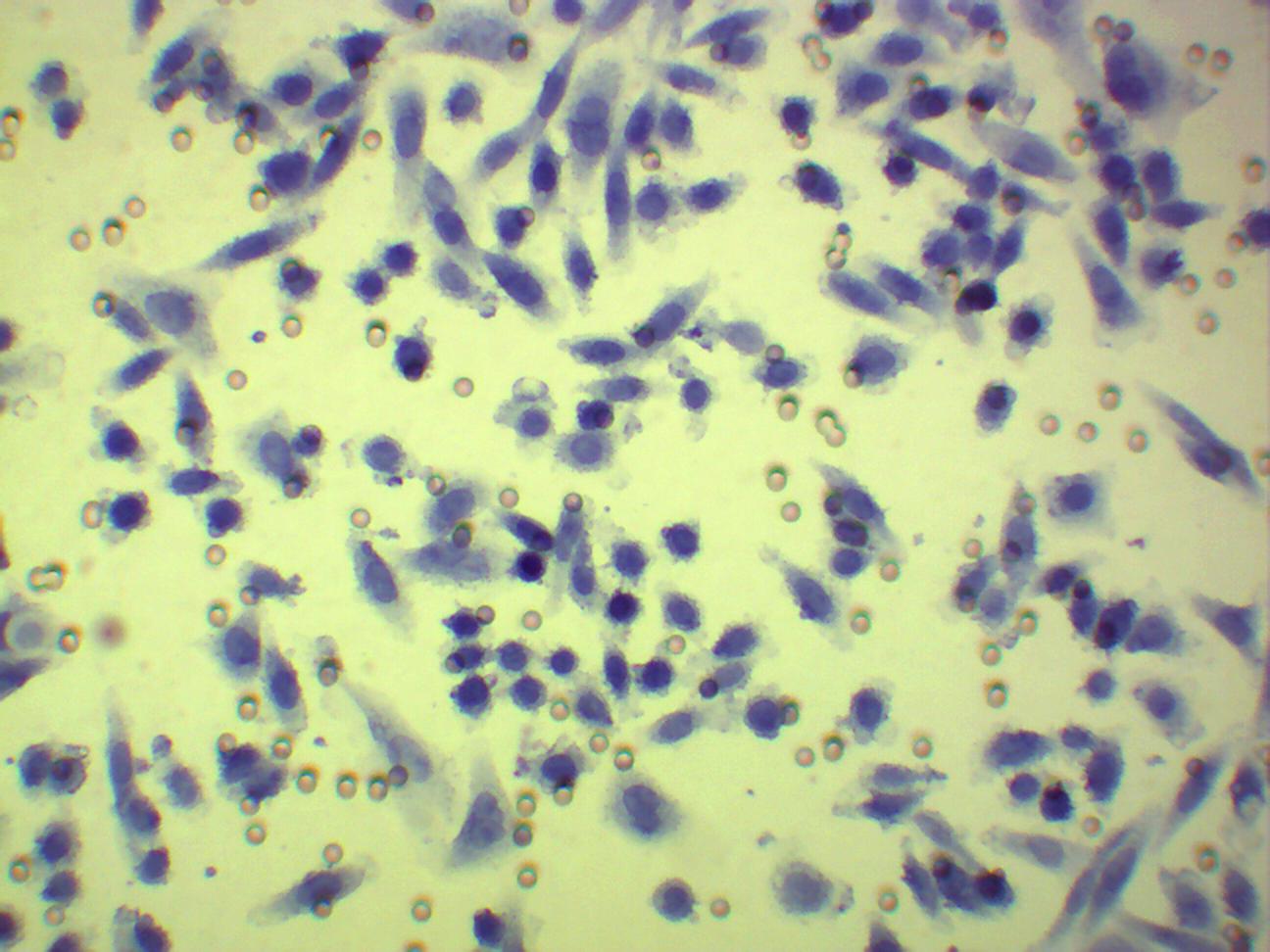

Supplement: Supplementary file 1 — Supplementary figures and tables. [file jcav14p2739s1.zip › supplementary/raw data/Figure 5/SW620/E2F4+MNX1 (1).JPG]

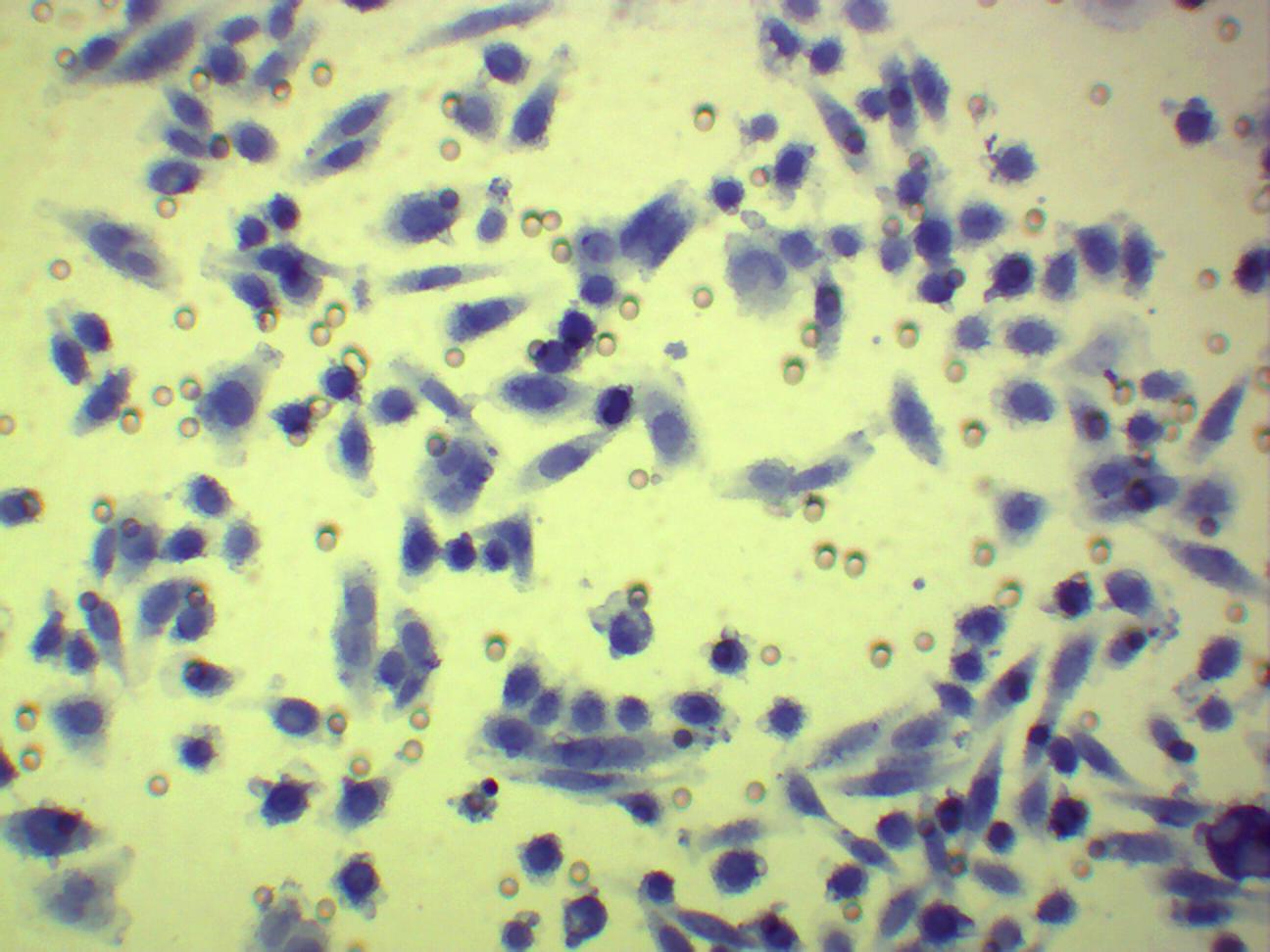

Supplement: Supplementary file 1 — Supplementary figures and tables. [file jcav14p2739s1.zip › supplementary/raw data/Figure 5/SW620/E2F4+MNX1 (2).JPG]

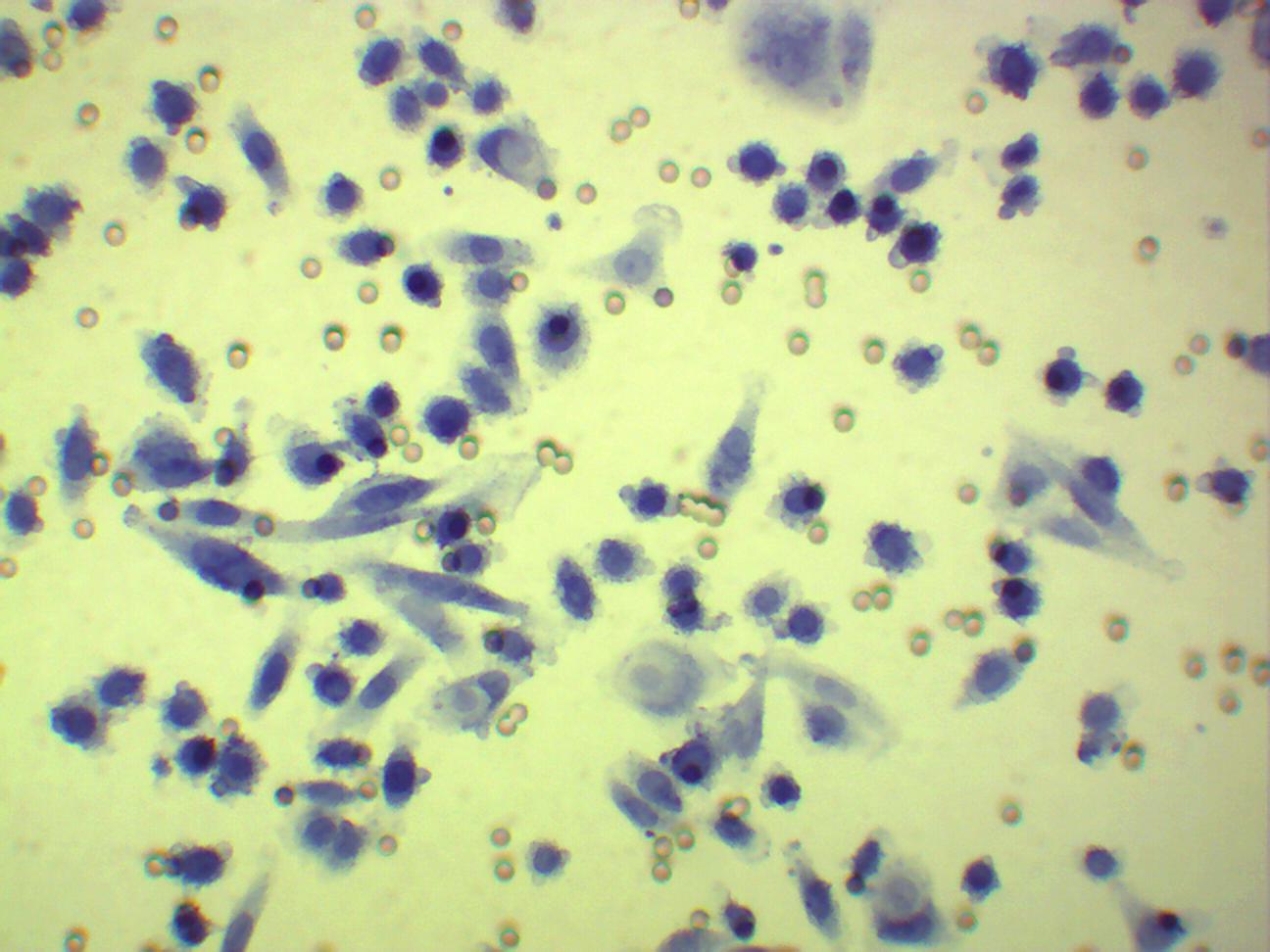

Supplement: Supplementary file 1 — Supplementary figures and tables. [file jcav14p2739s1.zip › supplementary/raw data/Figure 5/SW620/E2F4+MNX1(1).JPG]

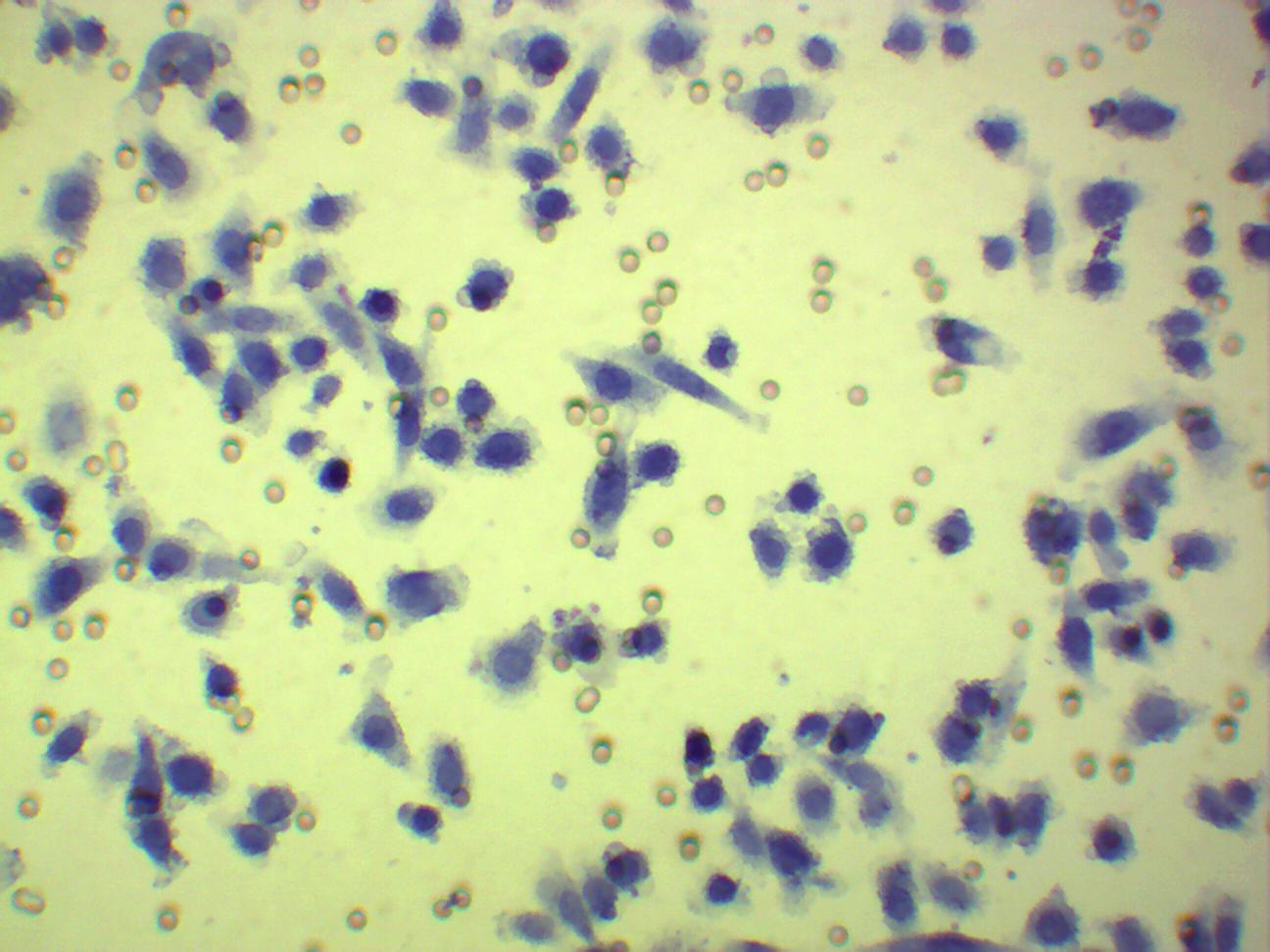

Supplement: Supplementary file 1 — Supplementary figures and tables. [file jcav14p2739s1.zip › supplementary/raw data/Figure 5/SW620/E2F4+MNX1(2).JPG]

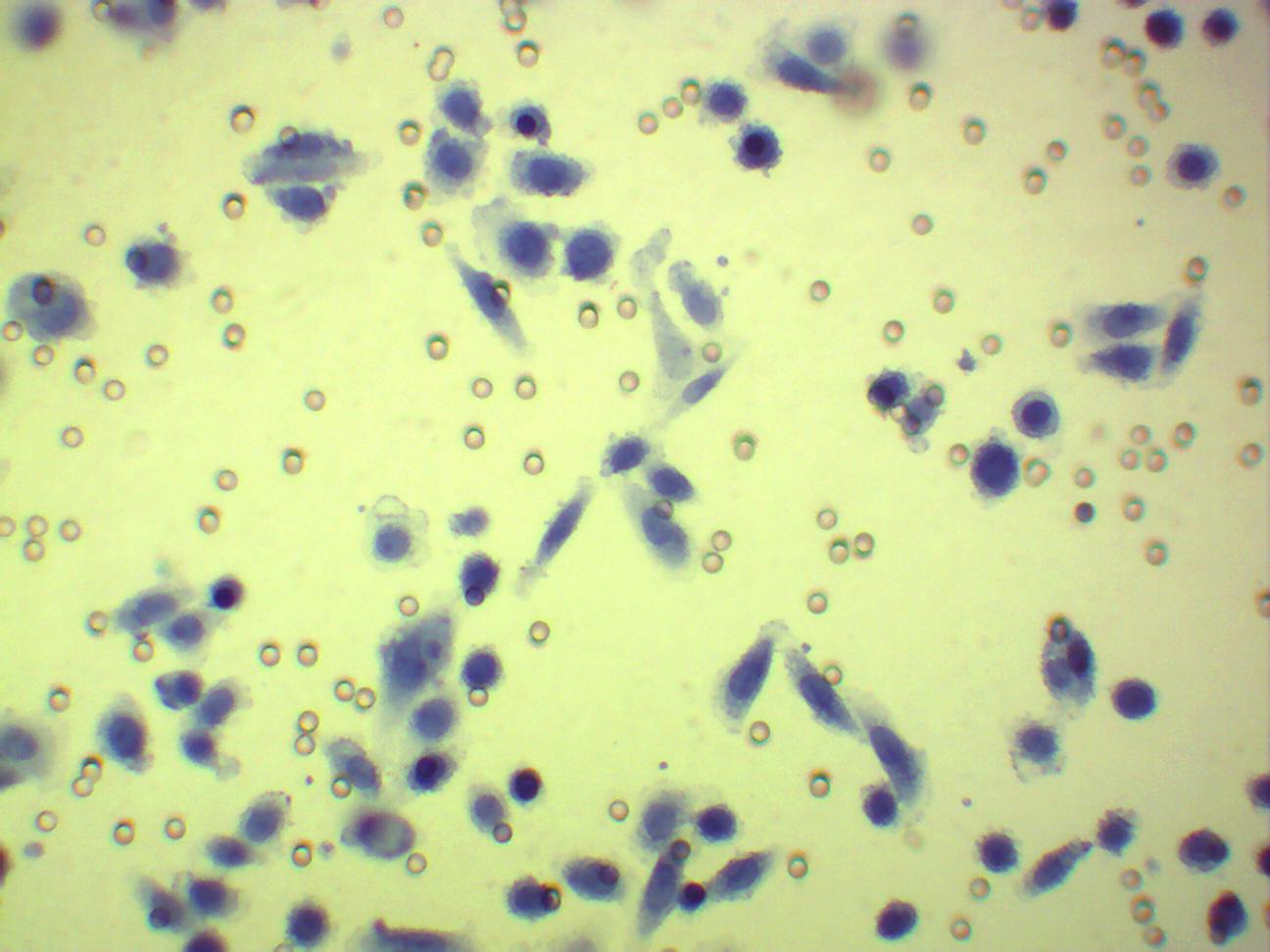

Supplement: Supplementary file 1 — Supplementary figures and tables. [file jcav14p2739s1.zip › supplementary/raw data/Figure 5/SW620/E2F4(1) .JPG]

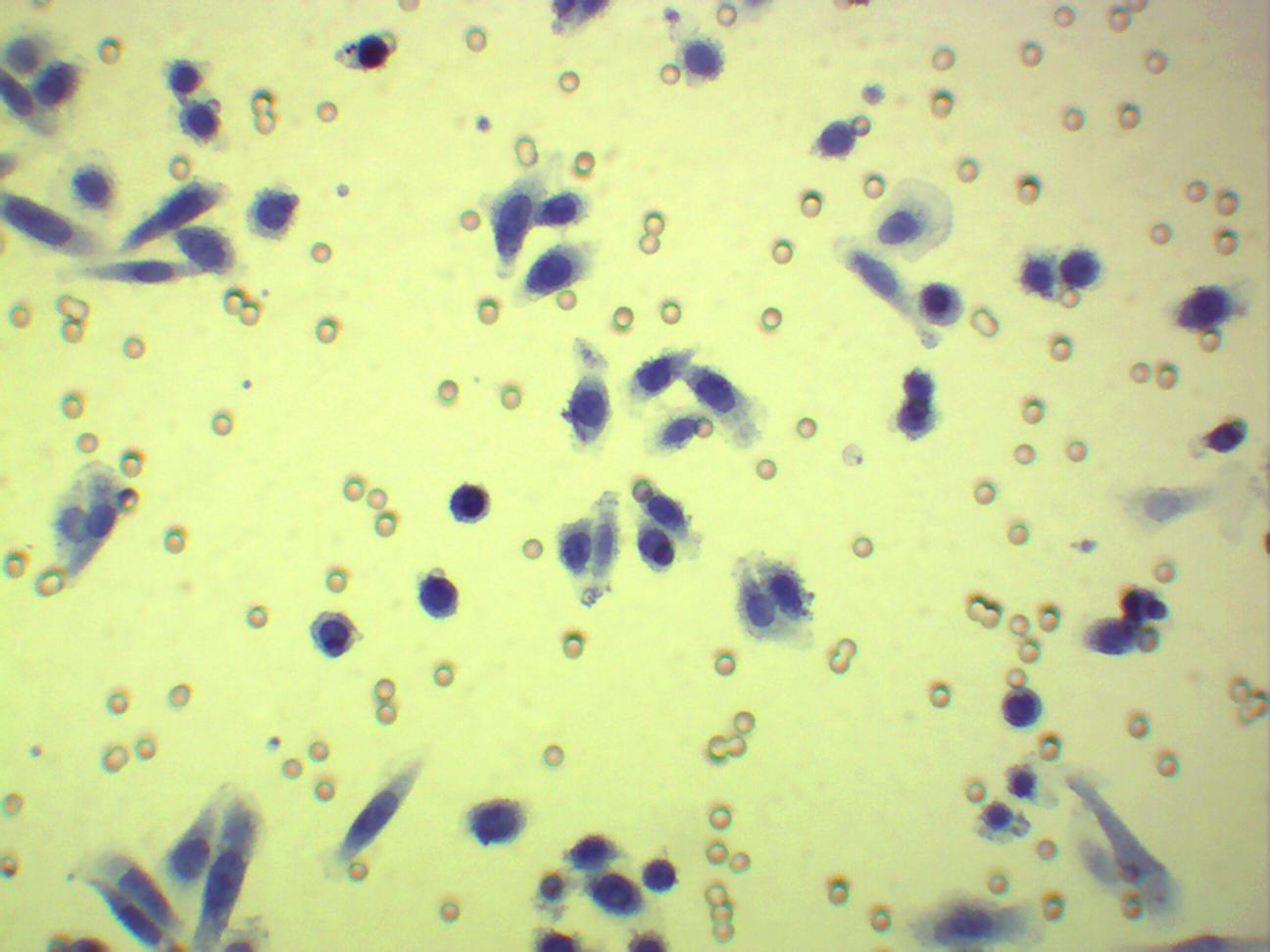

Supplement: Supplementary file 1 — Supplementary figures and tables. [file jcav14p2739s1.zip › supplementary/raw data/Figure 5/SW620/E2F4(1).JPG]

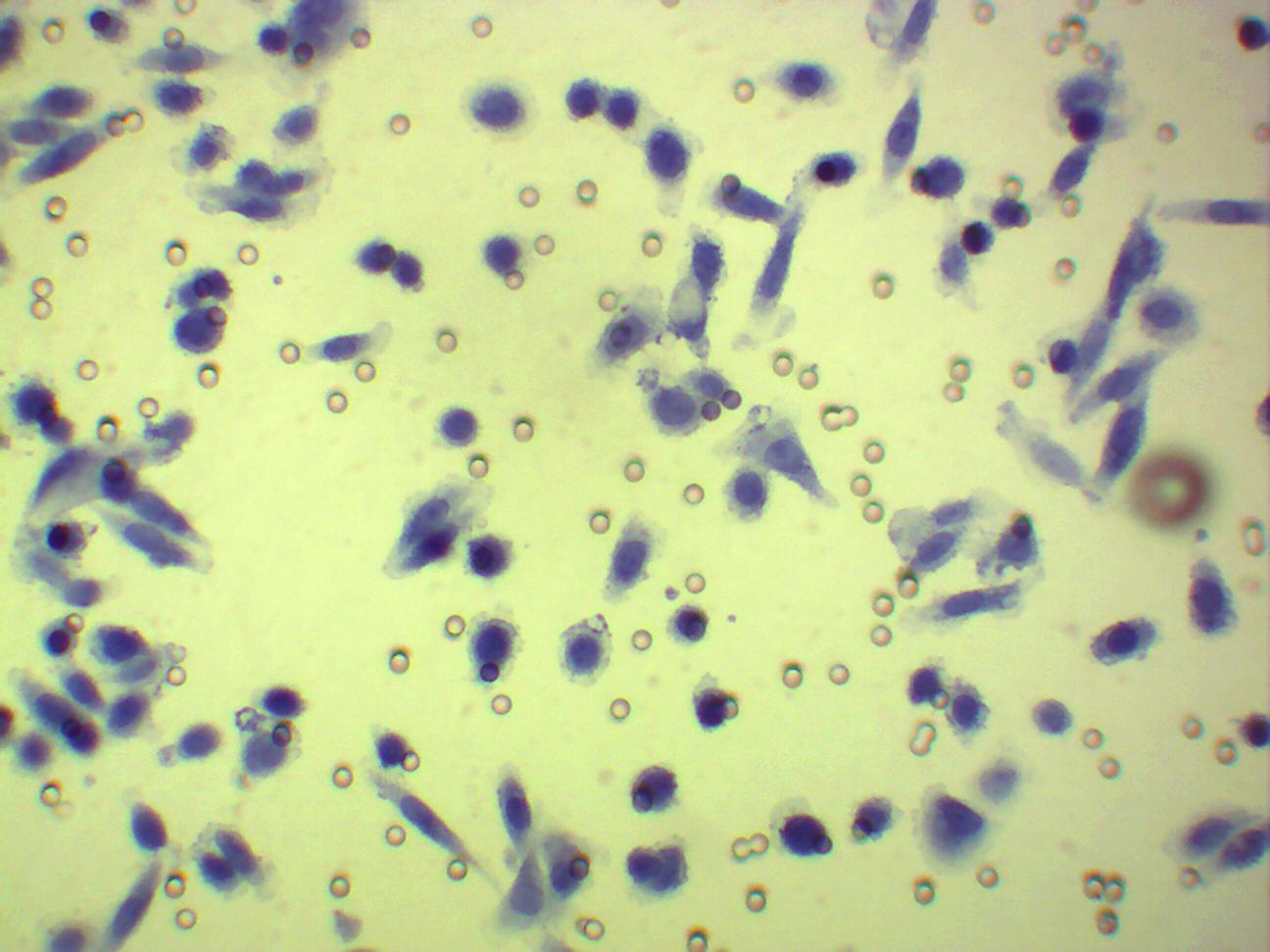

Supplement: Supplementary file 1 — Supplementary figures and tables. [file jcav14p2739s1.zip › supplementary/raw data/Figure 5/SW620/E2F4(2) .JPG]

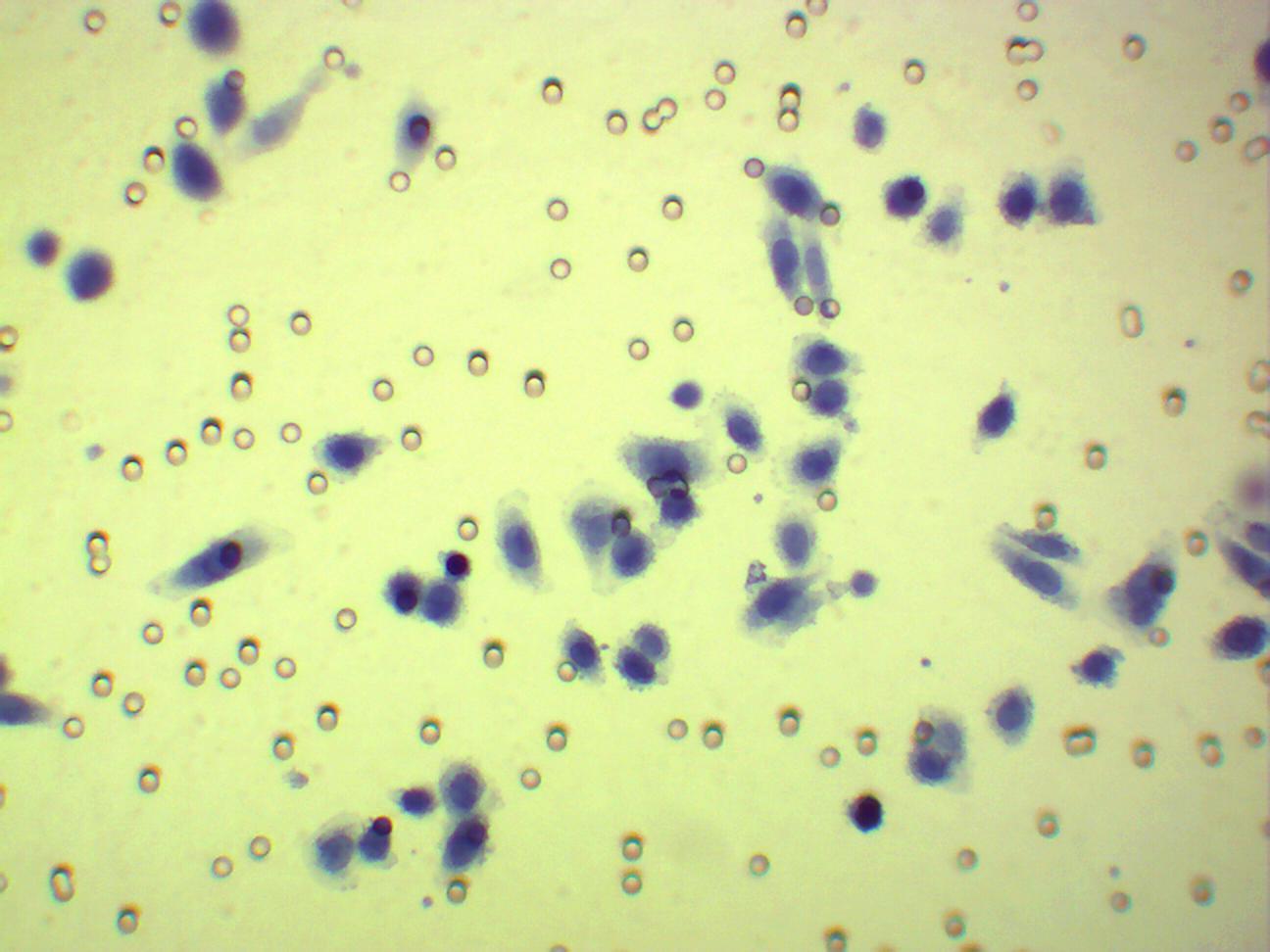

Supplement: Supplementary file 1 — Supplementary figures and tables. [file jcav14p2739s1.zip › supplementary/raw data/Figure 5/SW620/E2F4(2).JPG]

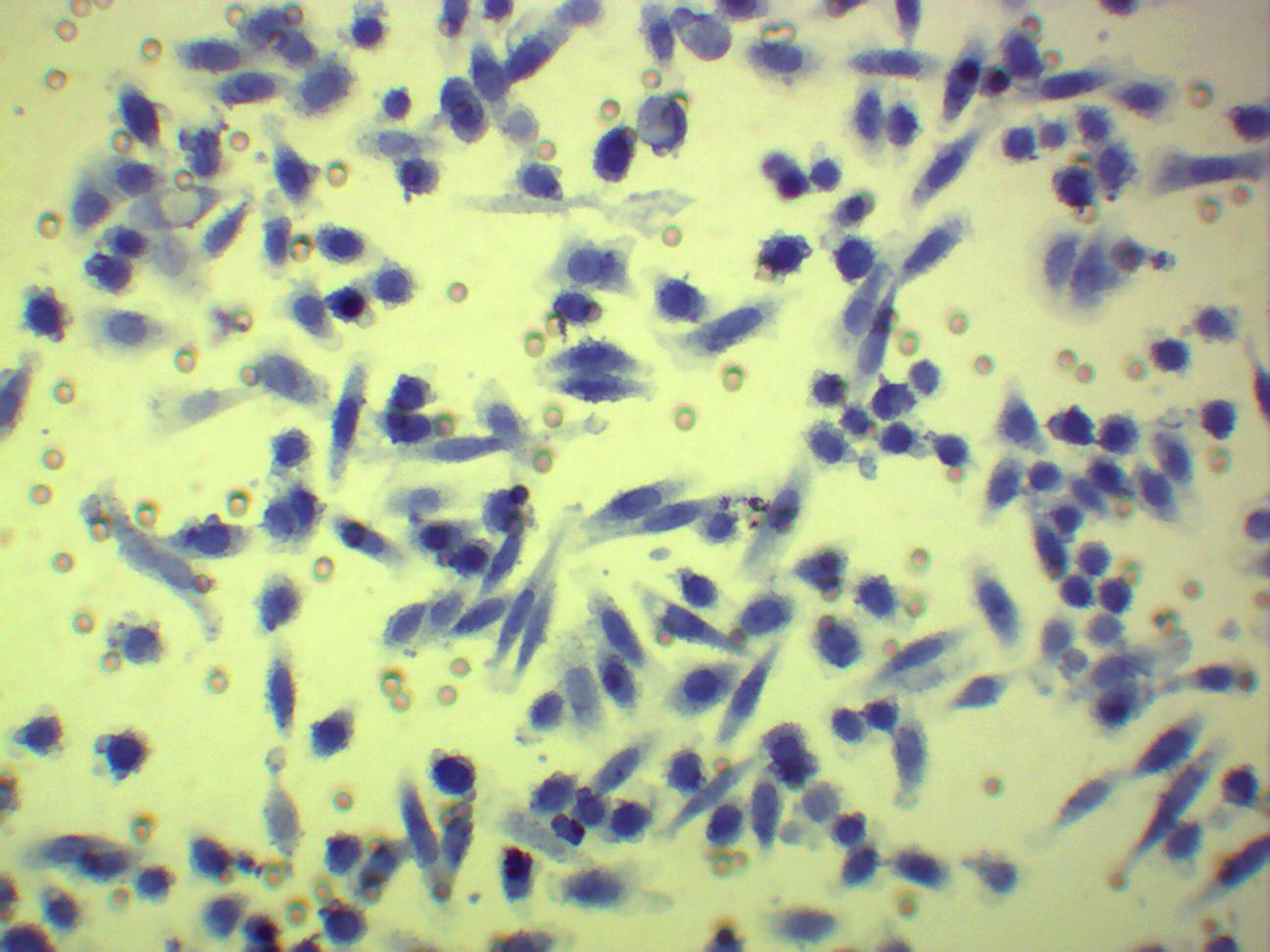

Supplement: Supplementary file 1 — Supplementary figures and tables. [file jcav14p2739s1.zip › supplementary/raw data/Figure 5/SW620/NC (1).JPG]

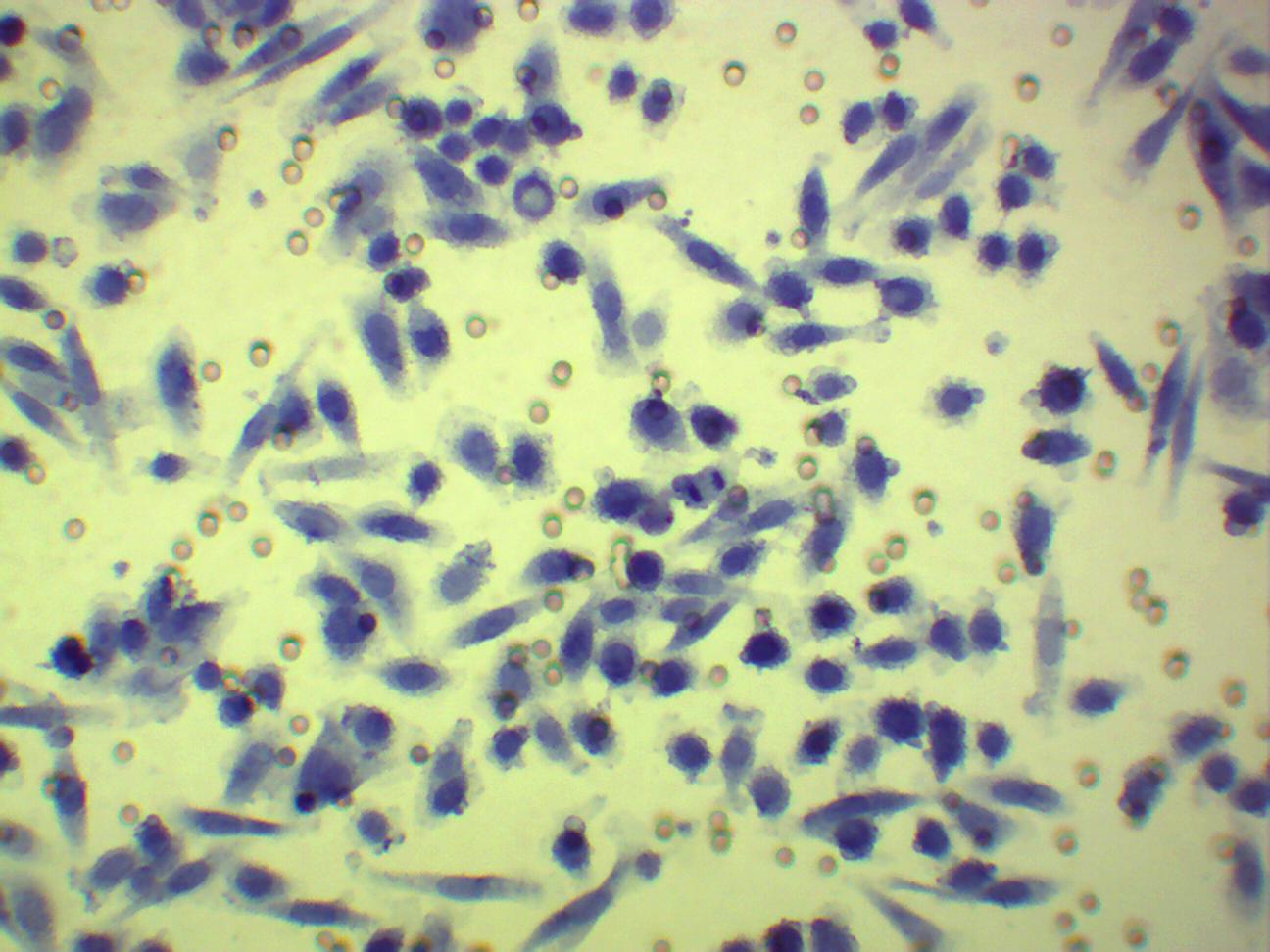

Supplement: Supplementary file 1 — Supplementary figures and tables. [file jcav14p2739s1.zip › supplementary/raw data/Figure 5/SW620/NC (2).JPG]

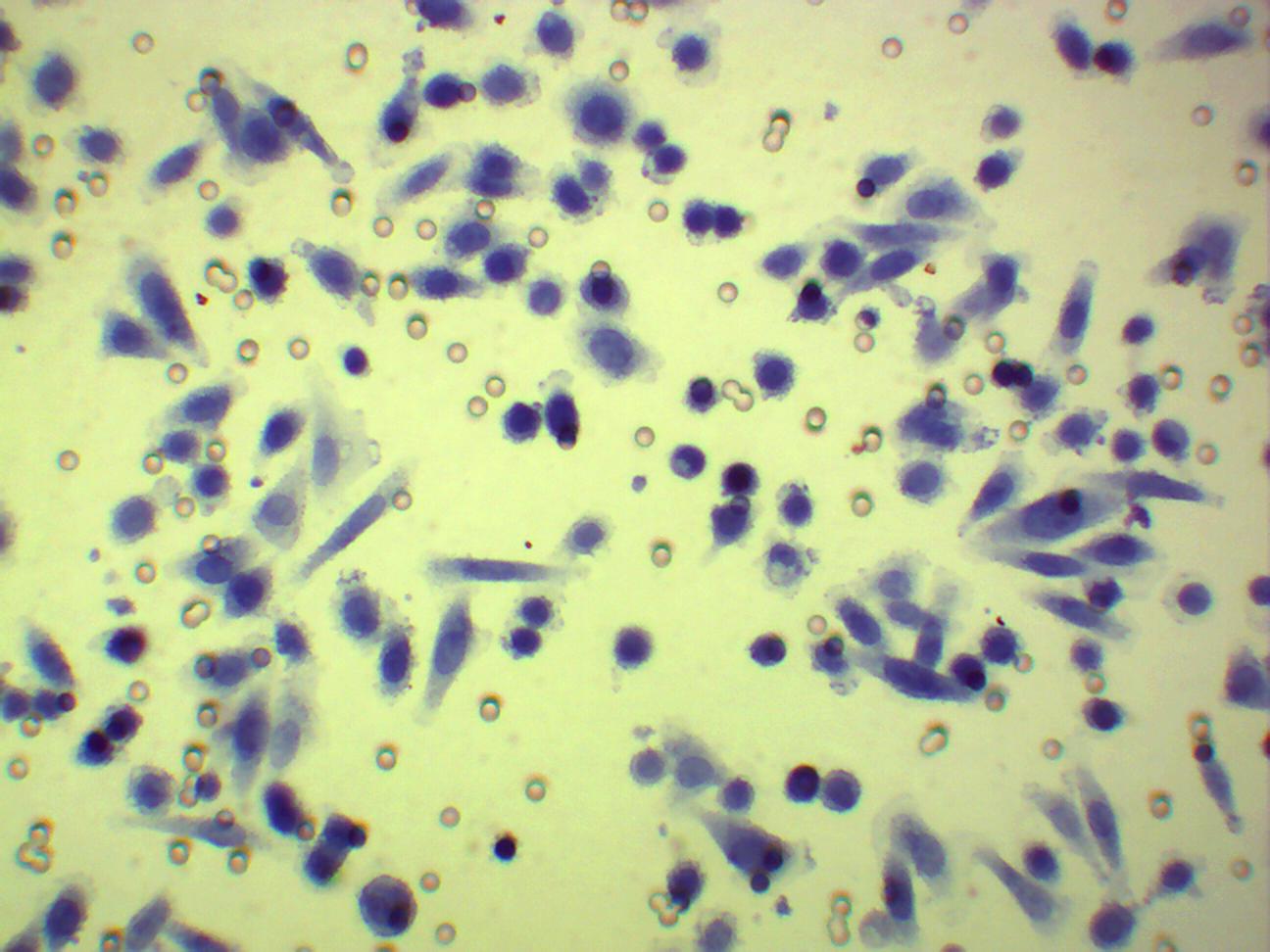

Supplement: Supplementary file 1 — Supplementary figures and tables. [file jcav14p2739s1.zip › supplementary/raw data/Figure 5/SW620/NC(1).JPG]

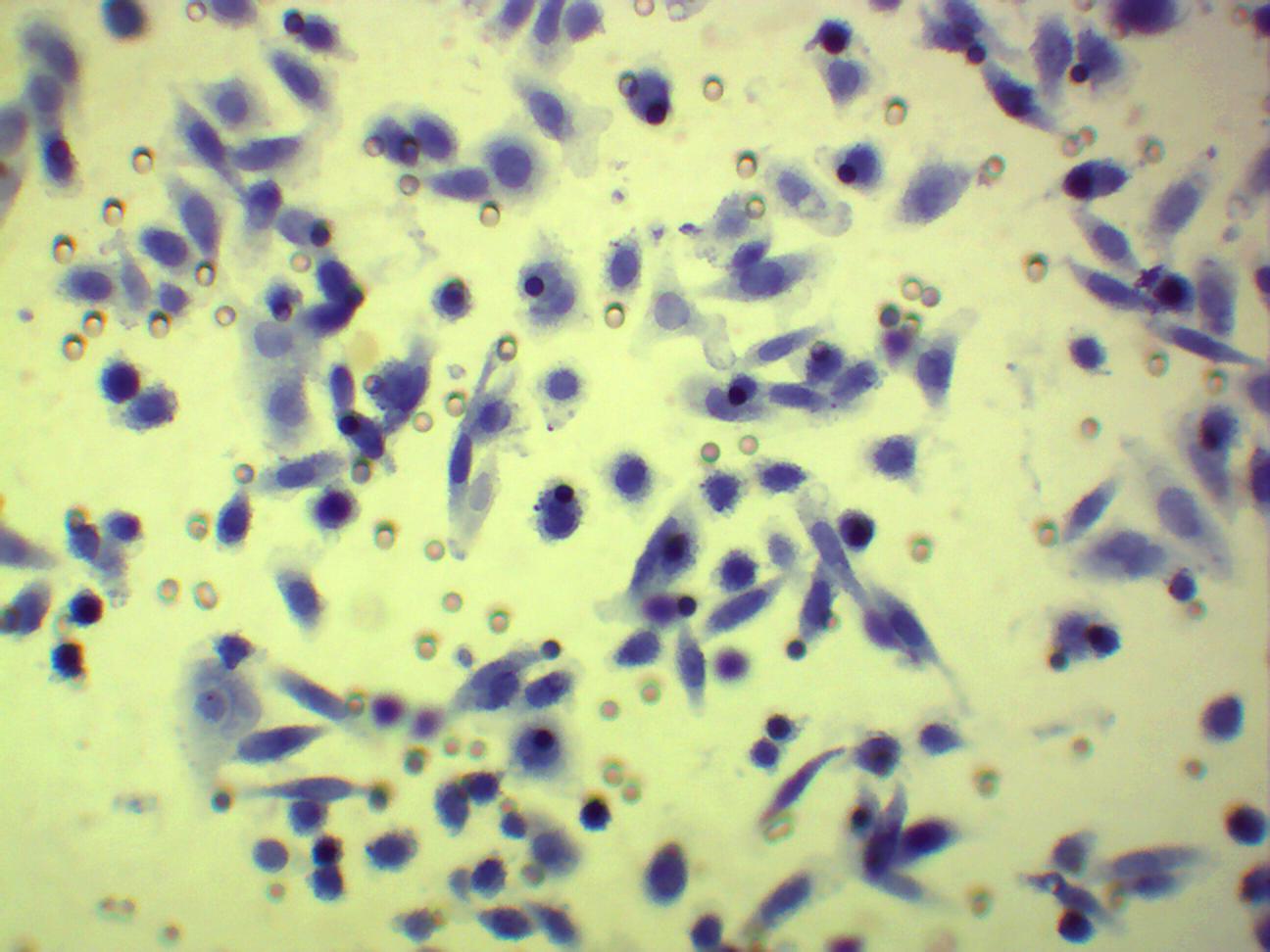

Supplement: Supplementary file 1 — Supplementary figures and tables. [file jcav14p2739s1.zip › supplementary/raw data/Figure 5/SW620/NC(2).JPG]

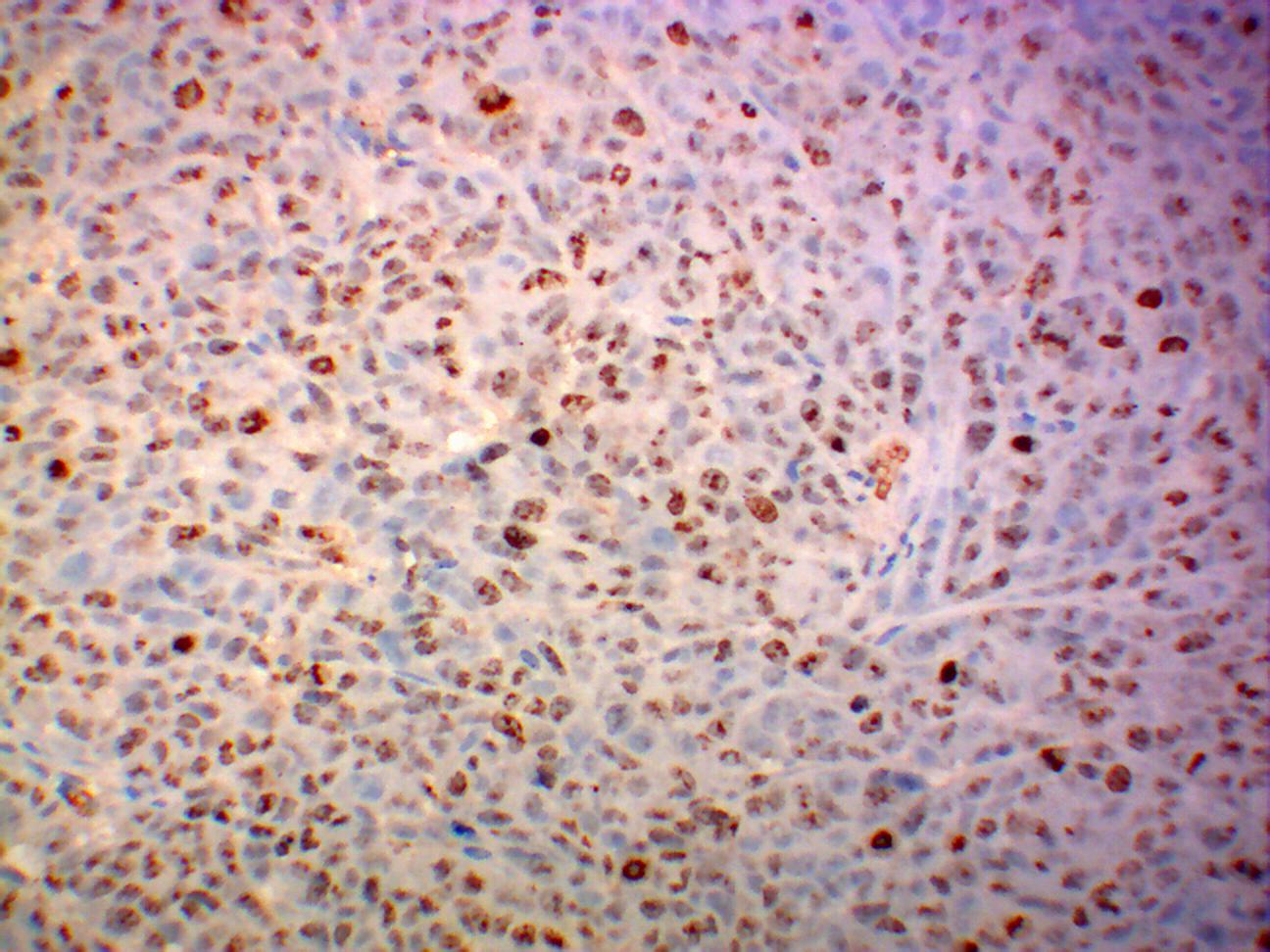

Supplement: Supplementary file 1 — Supplementary figures and tables. [file jcav14p2739s1.zip › supplementary/raw data/Figure 6/E2F4+MNX1(1-1).JPG]

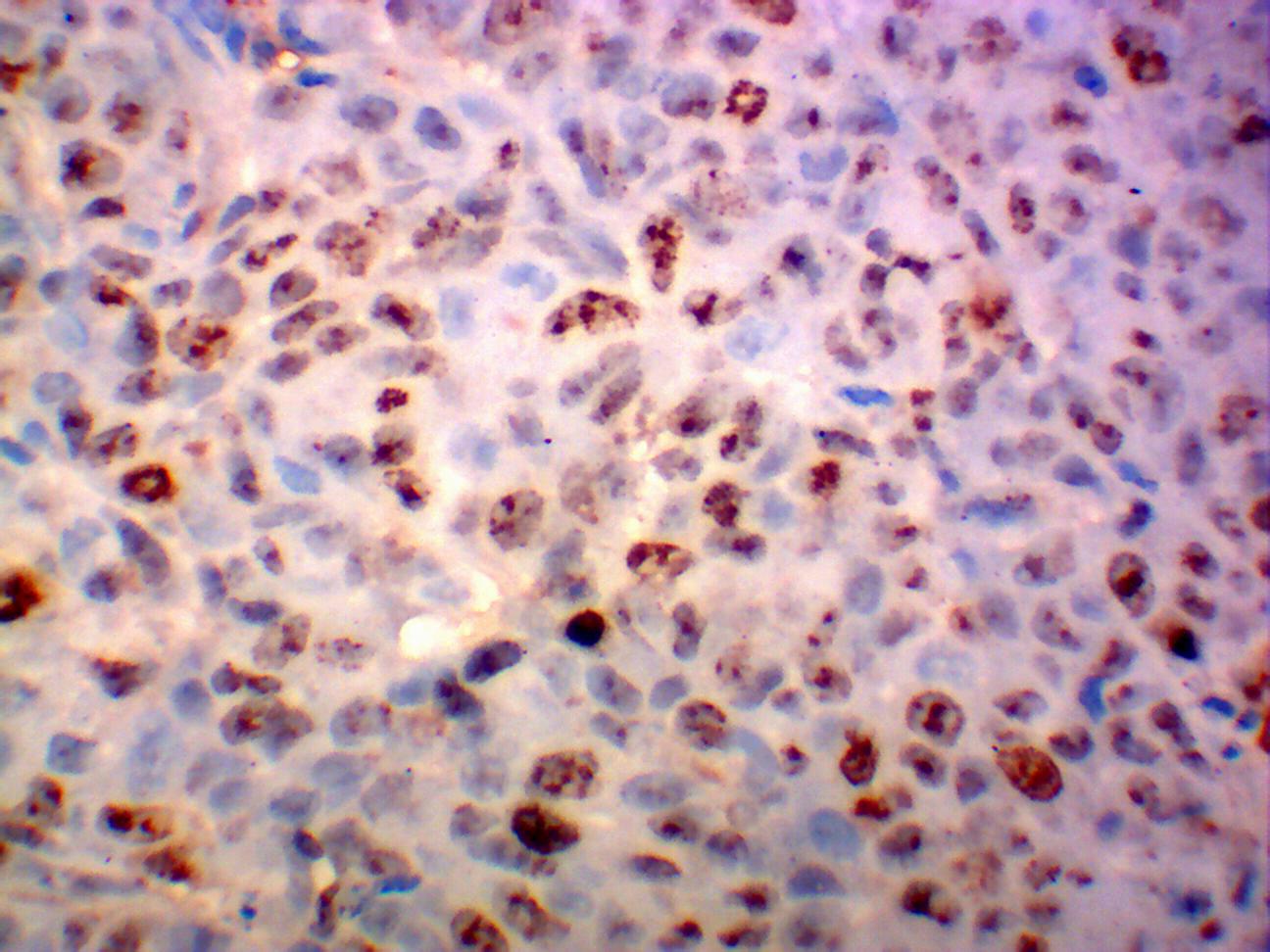

Supplement: Supplementary file 1 — Supplementary figures and tables. [file jcav14p2739s1.zip › supplementary/raw data/Figure 6/E2F4+MNX1(1-2).JPG]

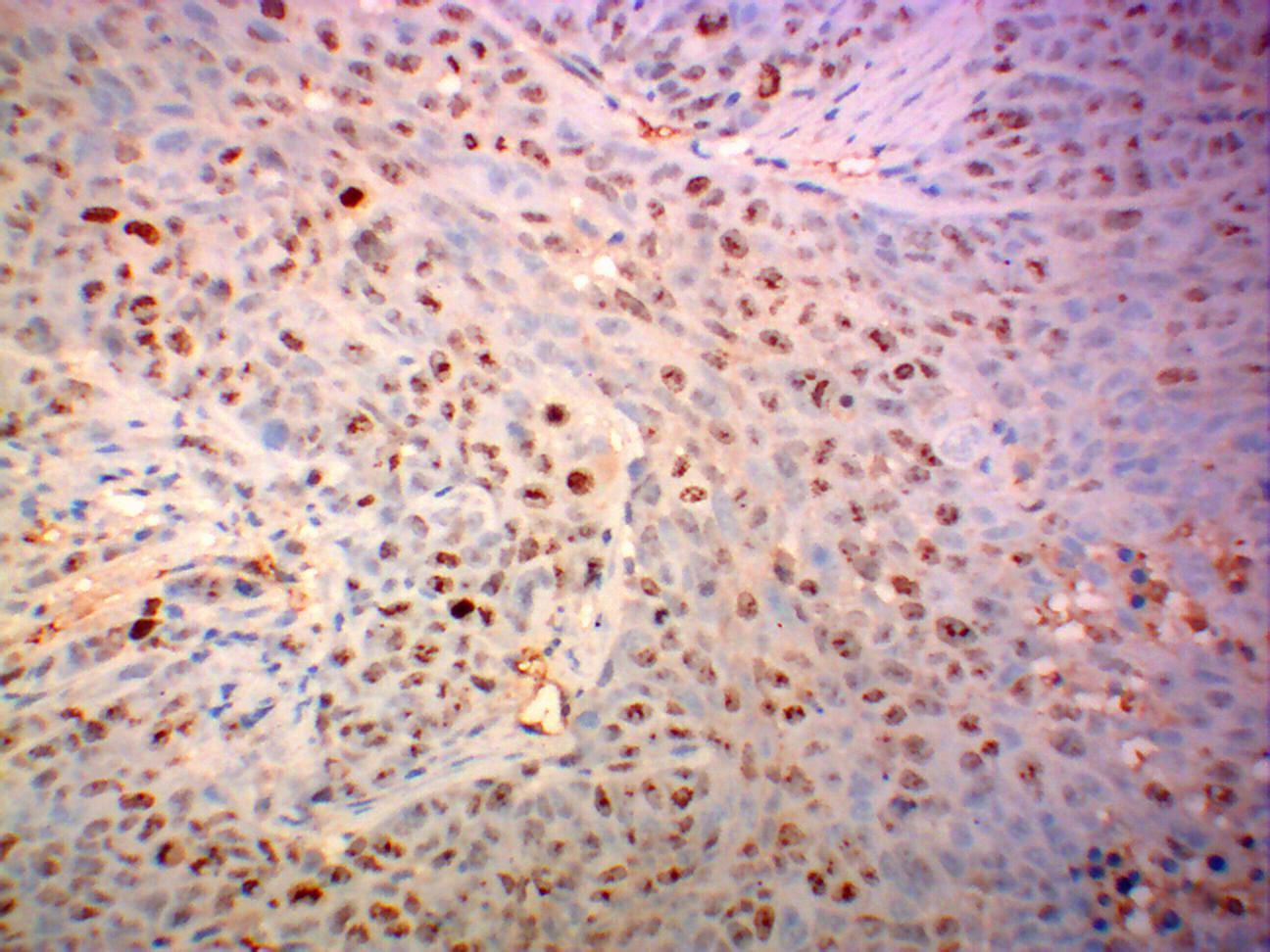

Supplement: Supplementary file 1 — Supplementary figures and tables. [file jcav14p2739s1.zip › supplementary/raw data/Figure 6/E2F4+MNX1(2-1).JPG]

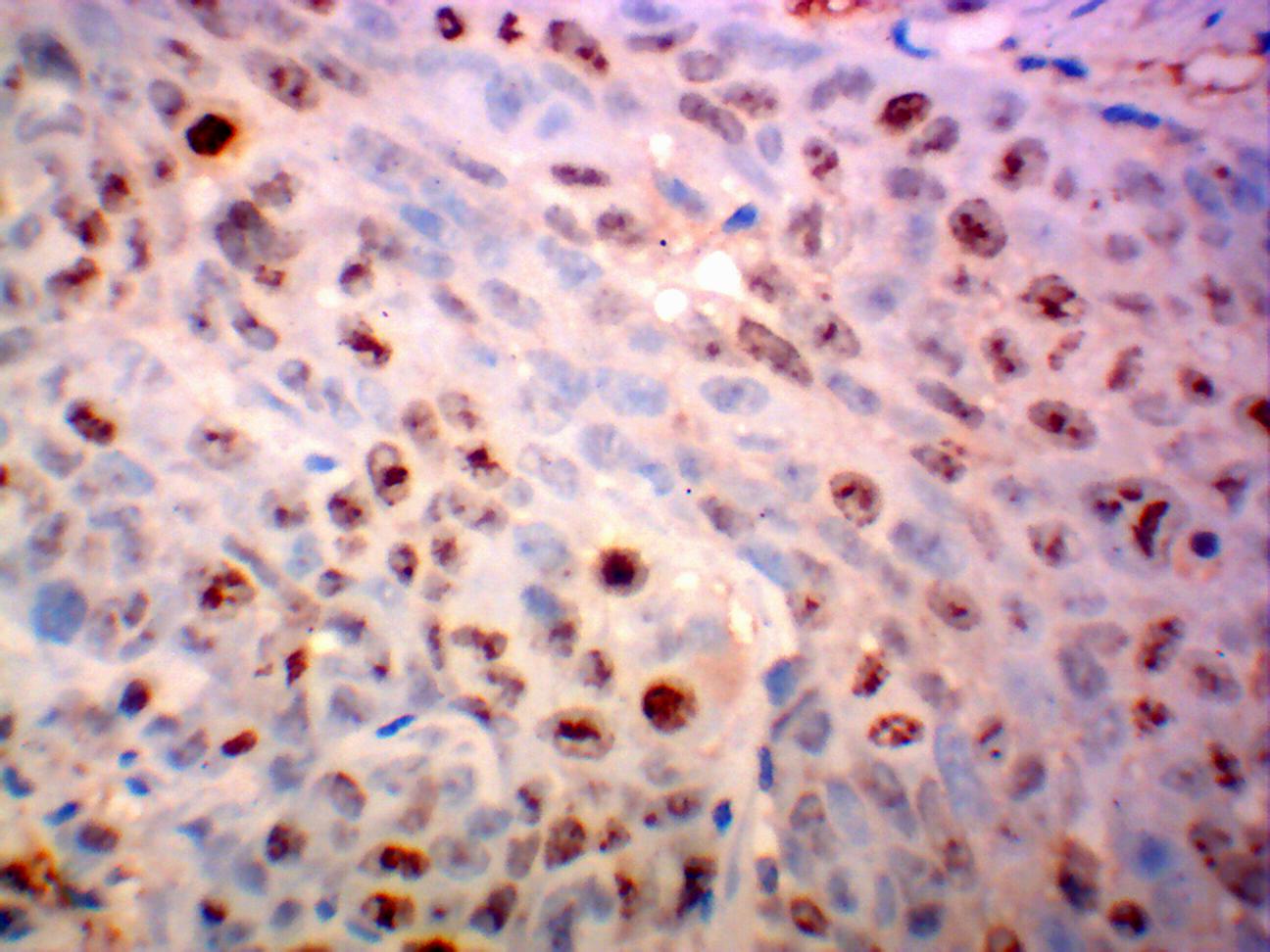

Supplement: Supplementary file 1 — Supplementary figures and tables. [file jcav14p2739s1.zip › supplementary/raw data/Figure 6/E2F4+MNX1(2-2).JPG]

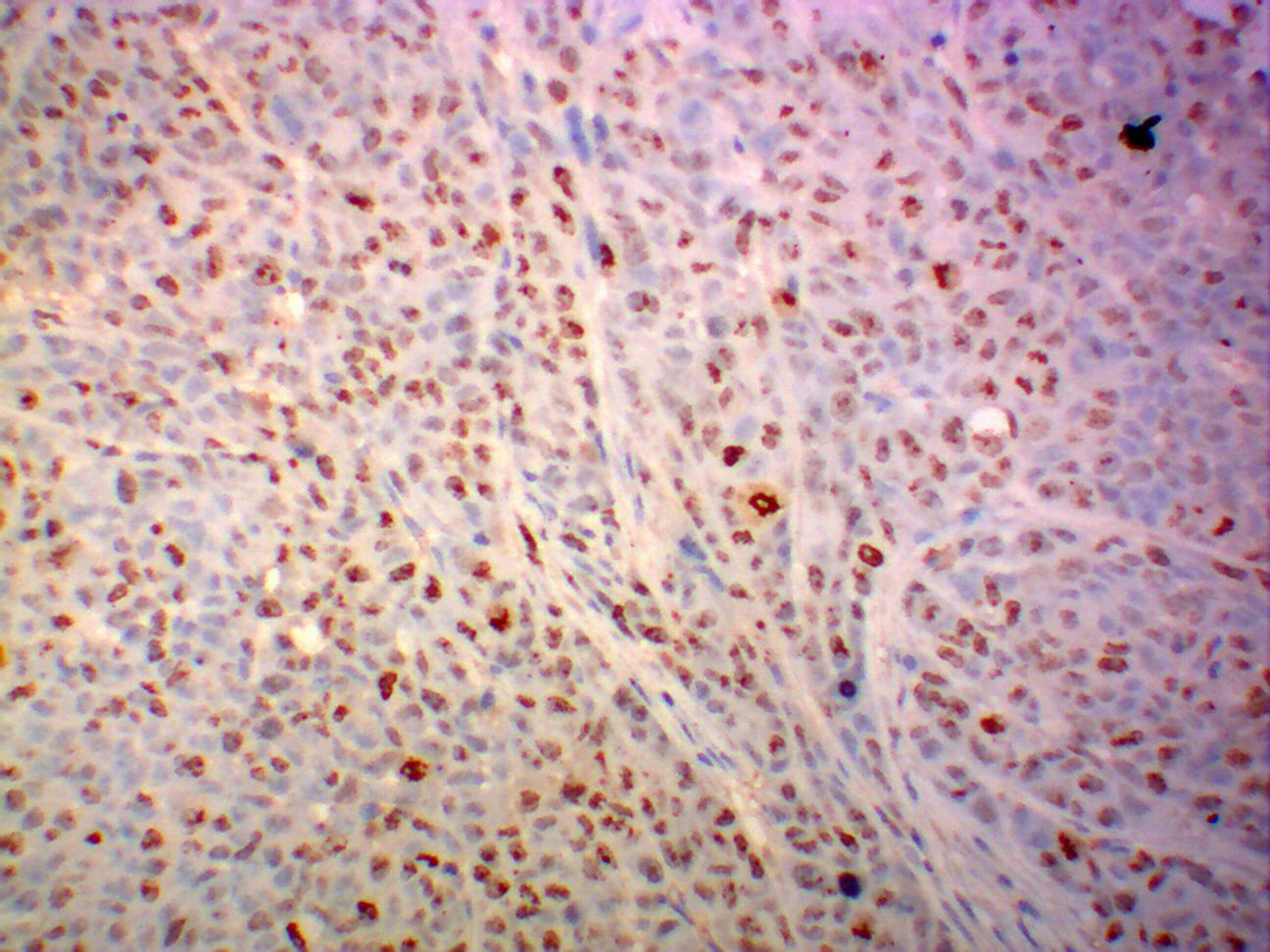

Supplement: Supplementary file 1 — Supplementary figures and tables. [file jcav14p2739s1.zip › supplementary/raw data/Figure 6/E2F4+MNX1(3-1).JPG]

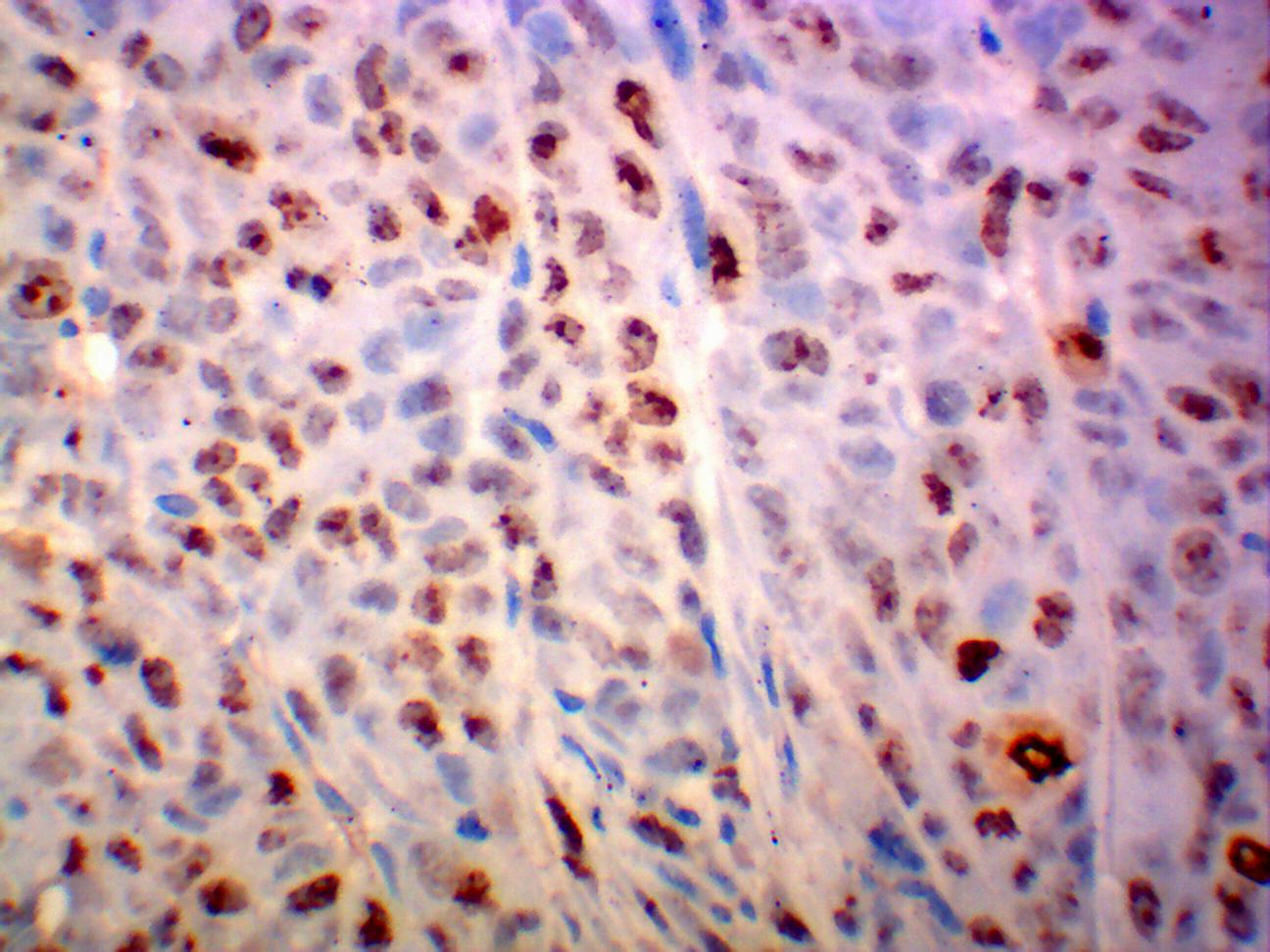

Supplement: Supplementary file 1 — Supplementary figures and tables. [file jcav14p2739s1.zip › supplementary/raw data/Figure 6/E2F4+MNX1(3-2).JPG]

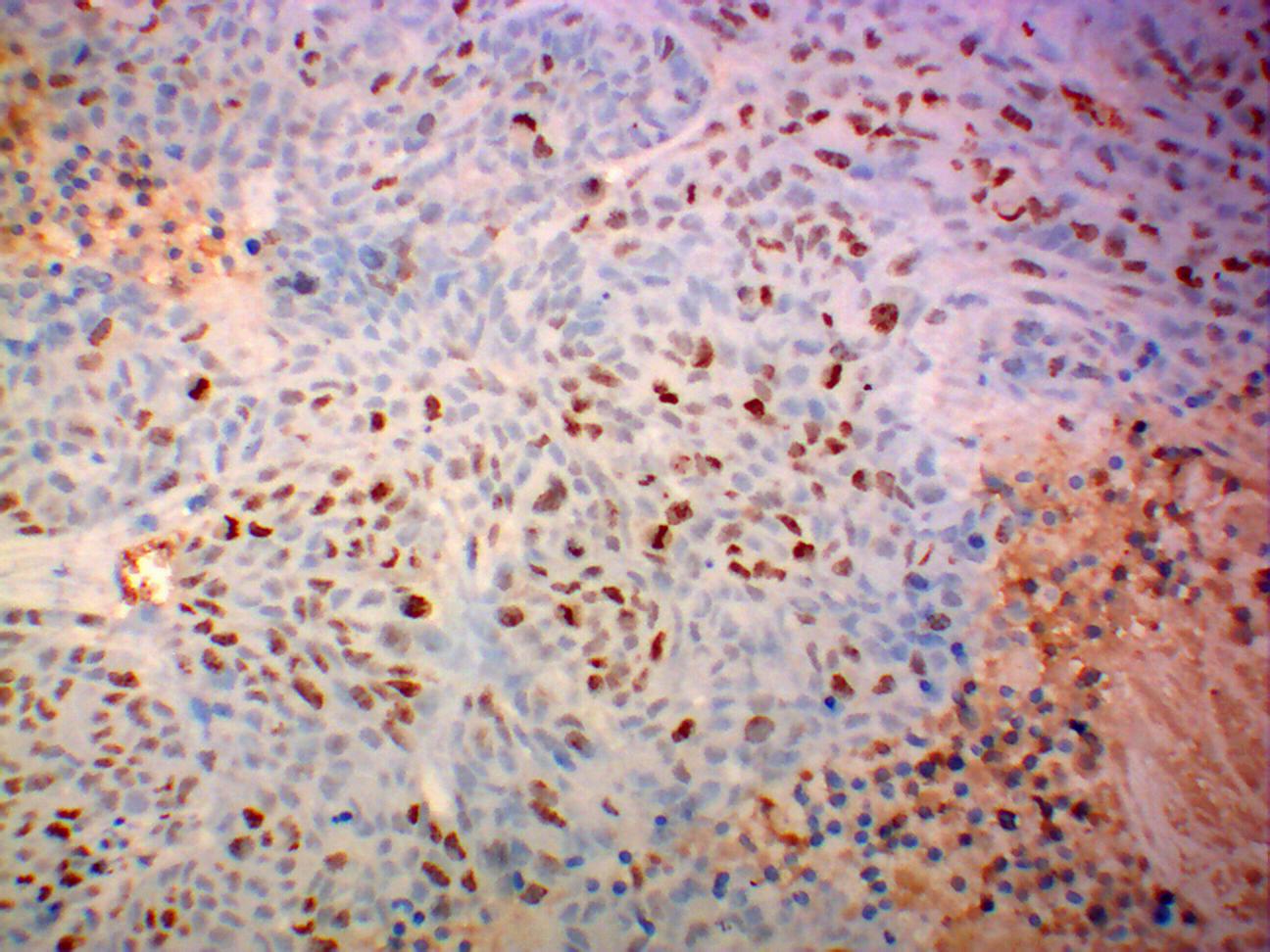

Supplement: Supplementary file 1 — Supplementary figures and tables. [file jcav14p2739s1.zip › supplementary/raw data/Figure 6/E2F4(1-1).JPG]

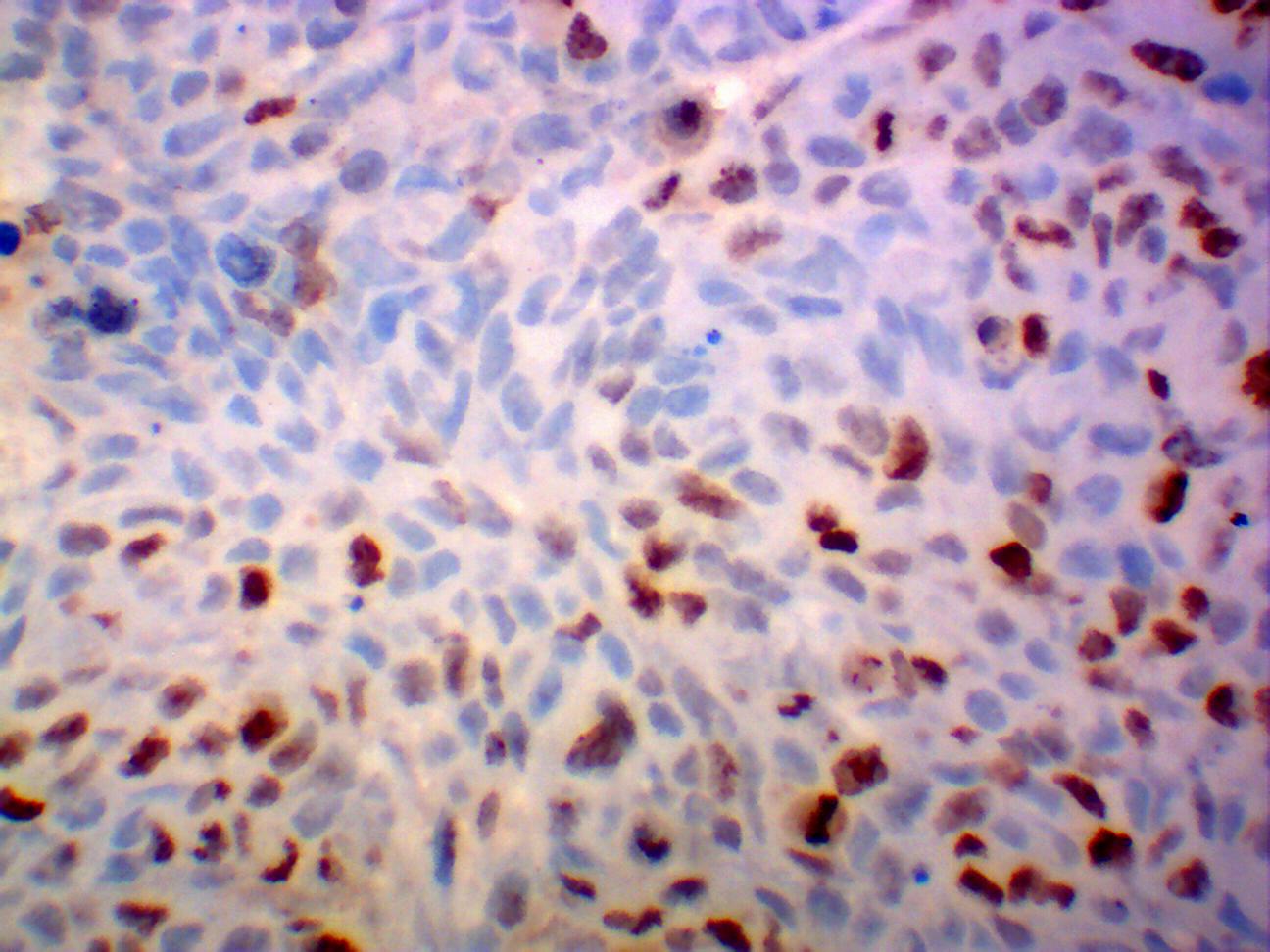

Supplement: Supplementary file 1 — Supplementary figures and tables. [file jcav14p2739s1.zip › supplementary/raw data/Figure 6/E2F4(1-2).JPG]

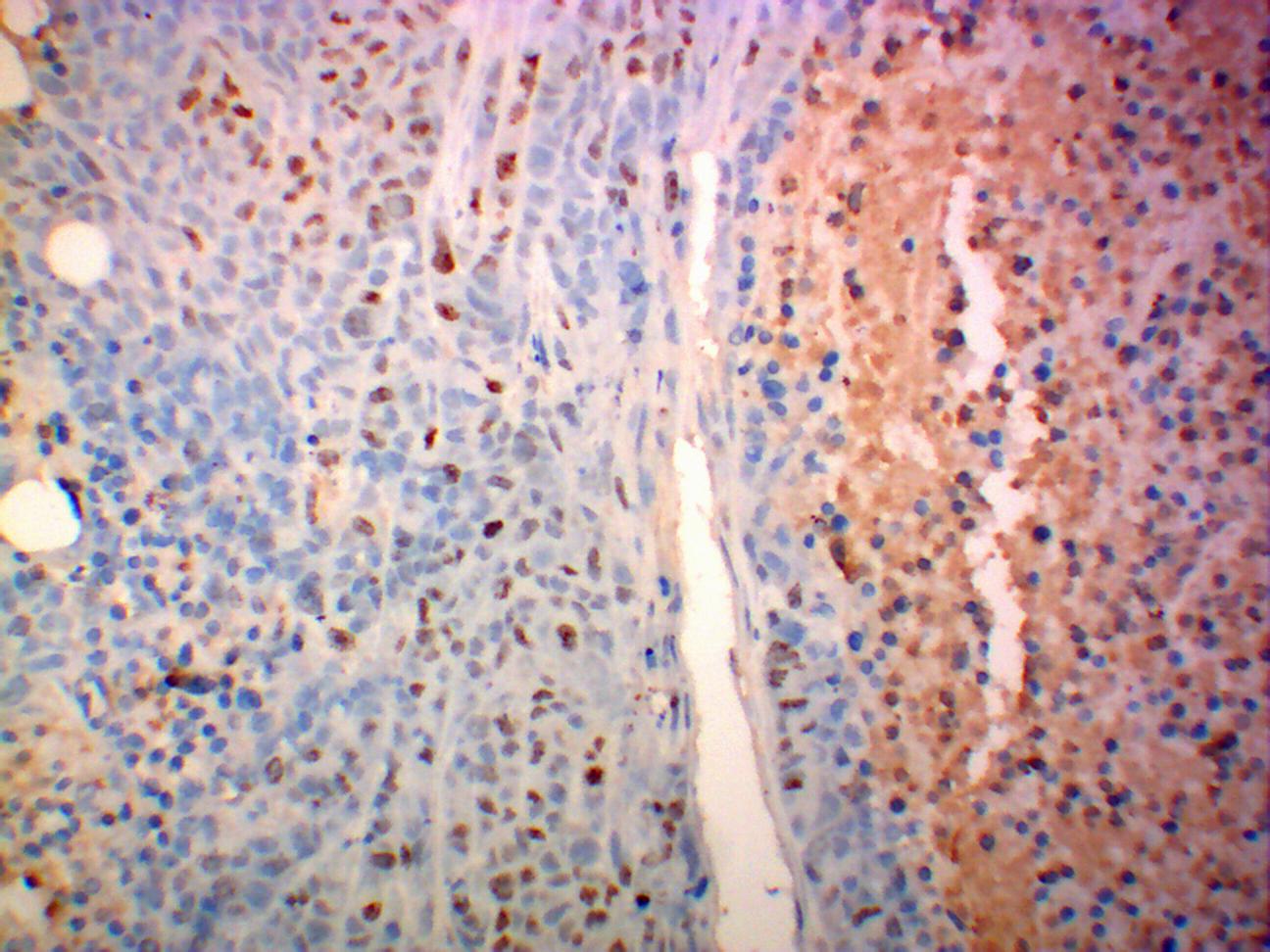

Supplement: Supplementary file 1 — Supplementary figures and tables. [file jcav14p2739s1.zip › supplementary/raw data/Figure 6/E2F4(2-1).JPG]

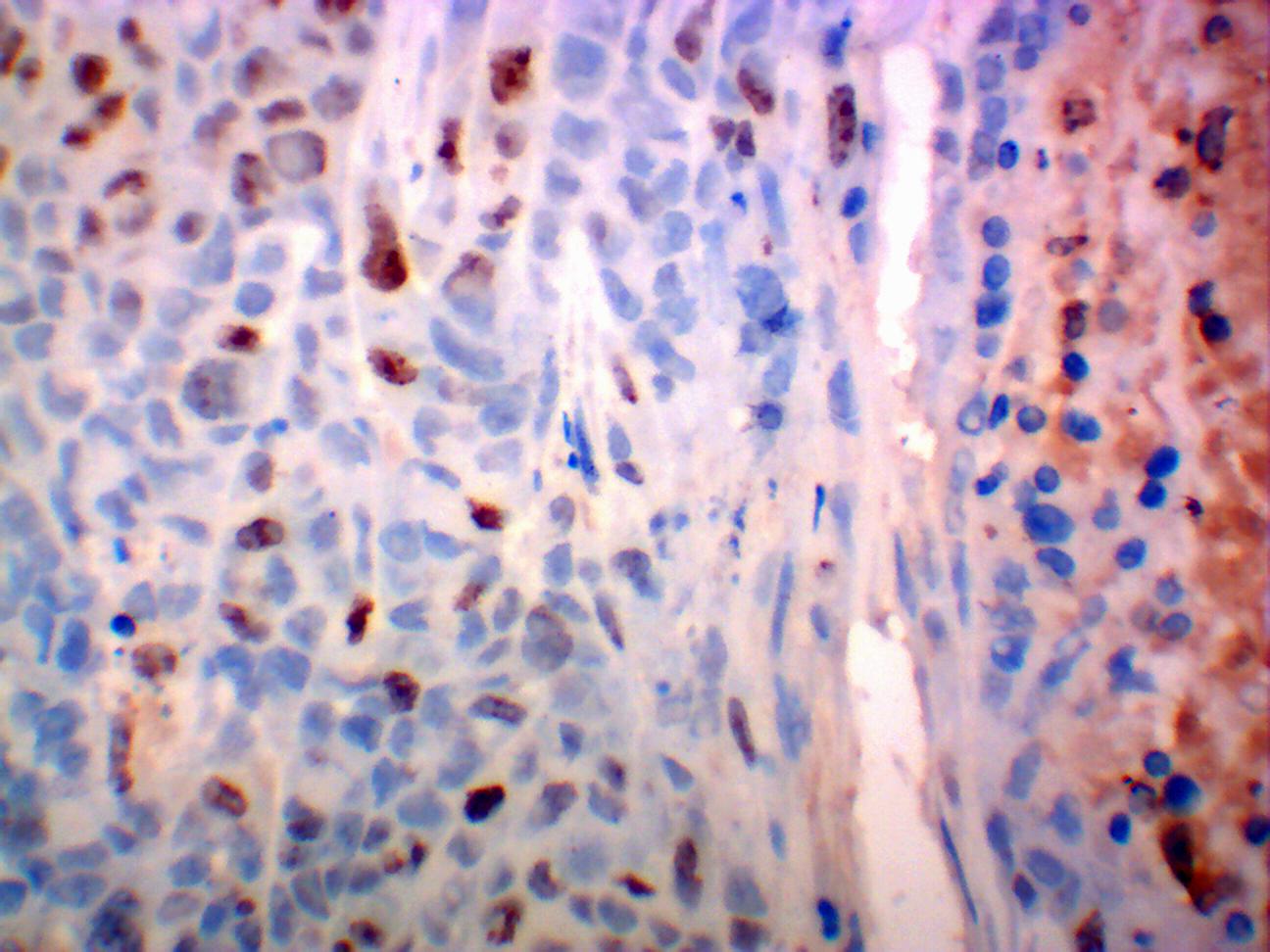

Supplement: Supplementary file 1 — Supplementary figures and tables. [file jcav14p2739s1.zip › supplementary/raw data/Figure 6/E2F4(2-2).JPG]

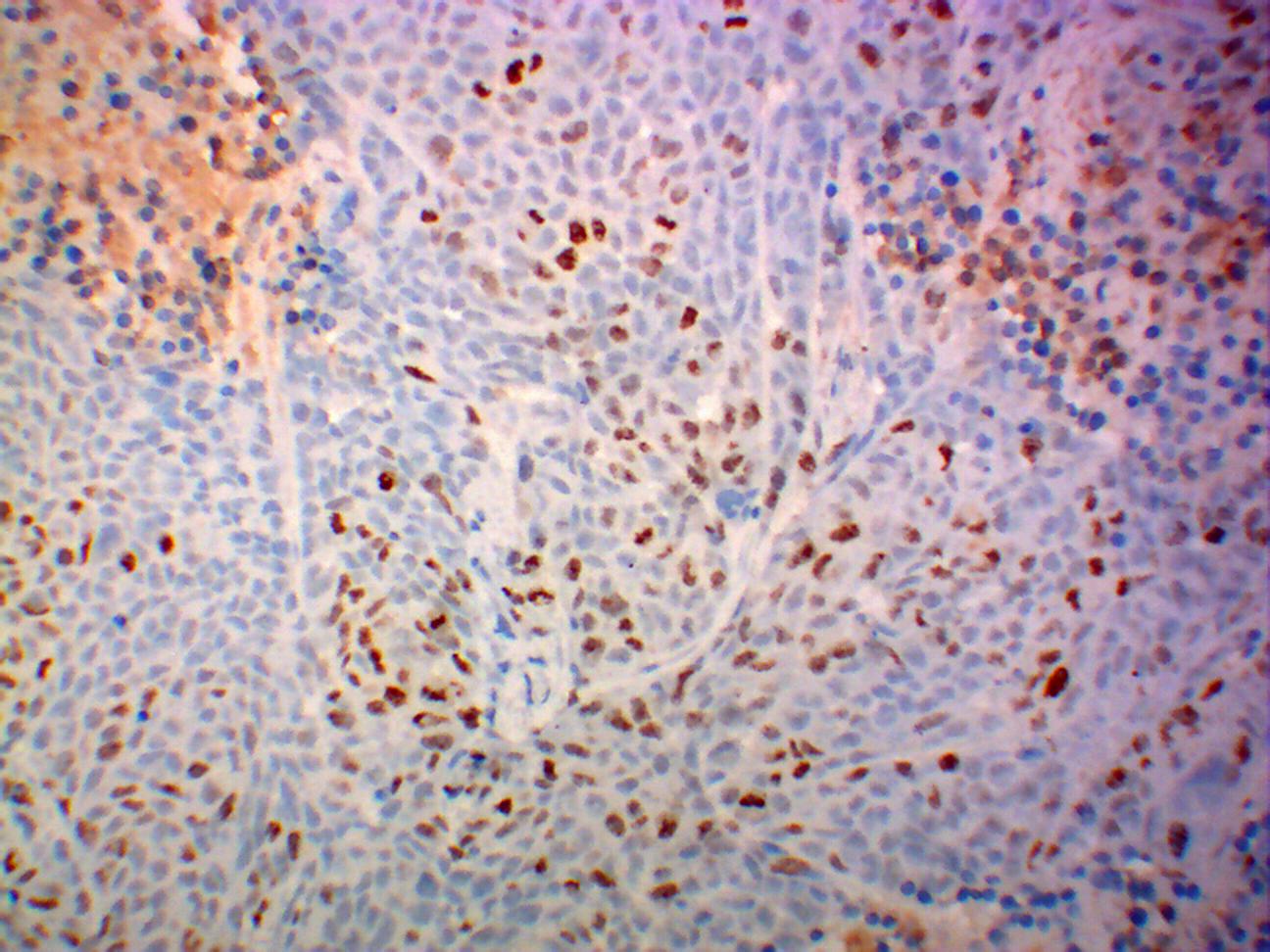

Supplement: Supplementary file 1 — Supplementary figures and tables. [file jcav14p2739s1.zip › supplementary/raw data/Figure 6/E2F4(3-1).JPG]

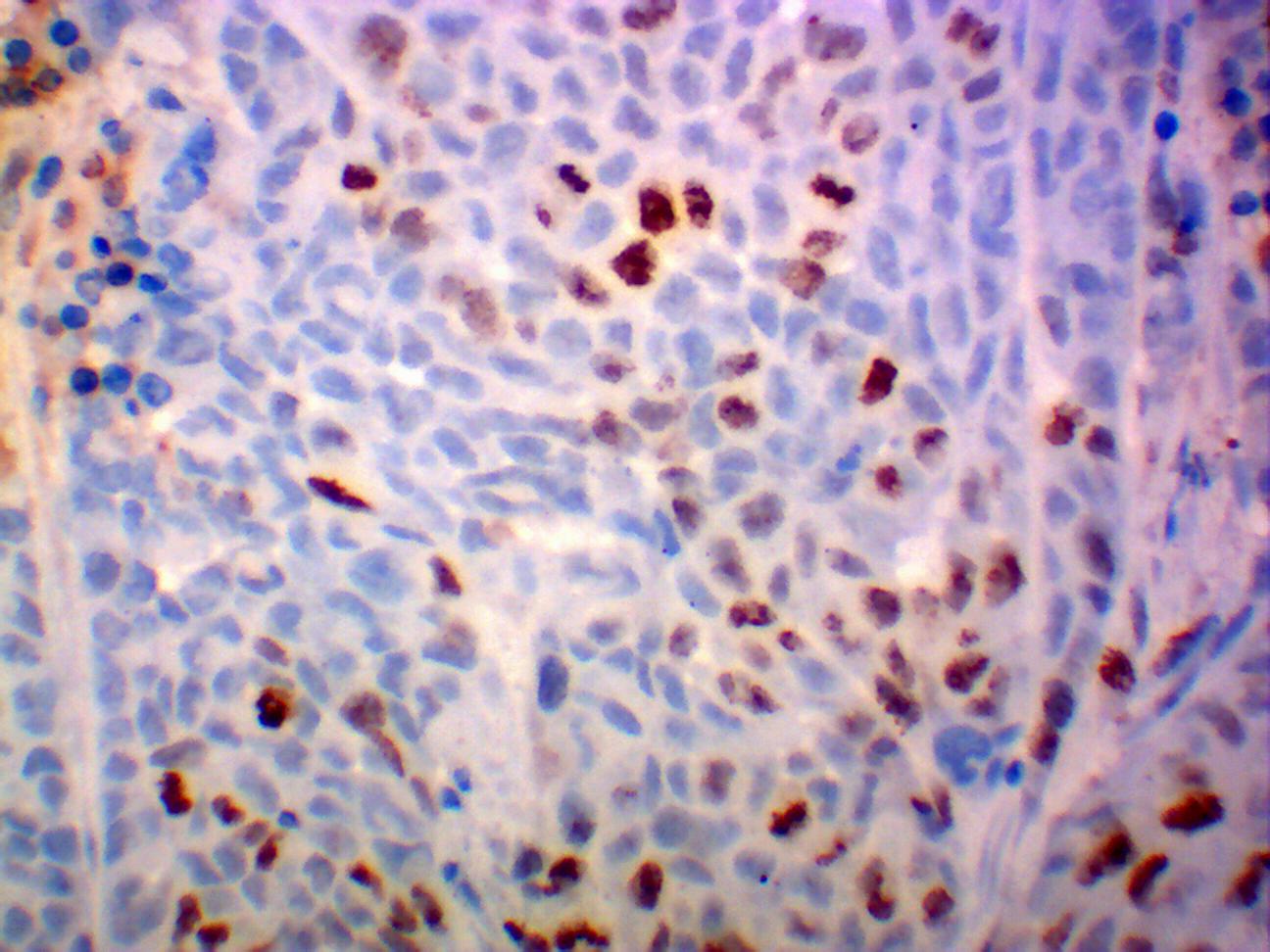

Supplement: Supplementary file 1 — Supplementary figures and tables. [file jcav14p2739s1.zip › supplementary/raw data/Figure 6/E2F4(3-2).JPG]
